# Mapping Physical Fitness Assessments in Interventional Research Among Breast Cancer Survivors: A Scoping Review

**DOI:** 10.3390/cancers18101642

**Published:** 2026-05-19

**Authors:** Minsuk Oh, Joonho Kong, Sihyeon Kim, Ji Won Kang, Yoon Jung Chang, Myung Ha Kim, Jihee Min

**Affiliations:** 1 Exercise Medicine Center for Diabetes and Cancer Patients, ICONS, Yonsei University, Seoul 03722, Republic of Korea; minsuk_oh@yonsei.ac.kr; 2 Department of Sports Industry, Yonsei University, Seoul 03722, Republic of Korea; 3 Department of Physical Education, Seoul National University of Education, Seoul 06639, Republic of Korea; 4 National Cancer Survivorship Center, National Cancer Control Institute, National Cancer Center, Goyang-si 10408, Republic of Korea; gasese1@yonsei.ac.kr (J.K.); ksh900731@dankook.ac.kr (S.K.); wldnjs6321@ncc.re.kr (J.W.K.); eunicemd@ncc.re.kr (Y.J.C.); 5 Medical Library, Yonsei University Wonju College of Medicine, Wonju 26426, Republic of Korea; xmankmh@yonsei.ac.kr; 6 National Cancer Center Graduate School of Cancer Science and Policy, National Cancer Center, Goyang-si 10408, Republic of Korea

**Keywords:** breast cancer survivors, physical fitness, cardiorespiratory fitness, muscular strength, exercise interventions, scoping review, PRISMA-ScR, rehabilitation, physical function, survivorship care

## Abstract

**Background/Objectives**: Breast cancer survivors differ from non-cancer populations in that cancer treatment commonly induces declines in physical fitness (PF) that are relevant to treatment tolerance, recovery, and survivorship outcomes. However, PF assessment methods vary widely across interventional studies. This scoping review aimed to map and characterize objective PF assessments used in interventional research among breast cancer survivors. **Methods**: Following PRISMA-ScR guidelines, PubMed, MEDLINE, EMBASE, Web of Science, KoreaMed, KCI, ClinicalTrials.gov, and Google Scholar were searched through November 2024. Interventional studies enrolling adult female breast cancer survivors and reporting at least one objectively measured PF outcome were included. **Results**: Of 9697 records identified, 316 studies met the inclusion criteria. Most interventions were exercise-based (83.2%) and conducted in the post-treatment phase (56.4%). Across the 316 studies, 557 PF domain records were identified because a single study could report more than one PF domain. Muscular strength accounted for the largest proportion of PF domain records (*n* = 222, 39.9%), followed by cardiorespiratory fitness (*n* = 190, 34.1%), flexibility (*n* = 100, 18.0%) and physical function (*n* = 45, 8.0%). Assessment method records showed that field-based walking test, strength test, shoulder range of motion test and balance tests were commonly used within their respective domain. Substantial heterogeneity in protocols, timing, and reporting limited comparability across studies. **Conclusions**: PF assessments are widely used in interventional breast cancer survivorship research but remain unevenly distributed across domains and treatment phases. More standardized and clearly reported PF assessment methods are needed to improve comparability and support future survivorship research.

## Simple Summary

Breast cancer survivors differ from women without cancer because surgery, chemotherapy, radiation, and endocrine therapy can lead to persistent declines in cardiorespiratory fitness, muscular strength, upper-extremity mobility, and physical function, along with fatigue and reduced quality of life. Because these impairments are clinically relevant to treatment tolerance, recovery, rehabilitation needs, and long-term survivorship outcomes, physical fitness assessment is particularly important in this population. However, intervention studies use widely varying fitness assessment methods, making it difficult to compare findings and identify clinically meaningful approaches. This scoping review mapped how physical fitness has been objectively assessed in interventional research among breast cancer survivors. We found substantial heterogeneity in assessment methods and underuse of flexibility and physical function outcomes, highlighting the need for more consistent reporting and context-appropriate, phase-specific fitness assessment strategies, as well as future evaluation of measurement properties where relevant, to improve survivorship care.

## 1. Introduction

Breast cancer is the most common malignancy among women worldwide and a leading cause of cancer-related morbidity and mortality [1]. Advances in early detection and treatment have substantially improved survival, resulting in a rapidly growing population of breast cancer survivors [2,3]. However, survivorship is often accompanied by persistent physical and psychological complications, including fatigue, impaired physical function, muscle atrophy, weight gain, and reduced quality of life [4,5,6]. Consequently, optimizing post-treatment recovery and long-term health outcomes has become a critical priority.

Compared with women without cancer, breast cancer survivors experience unique and often persistent treatment-related impairments that directly affect physical fitness (PF). Surgery and axillary procedures may lead to shoulder dysfunction, pain, and lymphedema risk; chemotherapy and radiation may reduce cardiorespiratory capacity and increase fatigue; and endocrine and systemic therapies may contribute to adverse changes in body composition, muscle strength, and physical functioning [4,5,6]. Thus, in breast cancer survivorship, PF is not merely a general wellness construct but a cancer-relevant clinical indicator linked to functional recovery, rehabilitation needs, and survivorship prognosis.

PF has emerged as a key physiological indicator in breast cancer populations, serving both as a measure of intervention efficacy and an independent prognostic factor [7,8,9]. Higher levels of cardiorespiratory fitness and muscular strength are consistently associated with improved treatment tolerance, reduced cancer-related fatigue, and better quality of life [7,10,11,12]. In addition, PF predicts long-term outcomes, including lower risks of recurrence and mortality, beyond the effects of physical activity alone [8,13]. Although physical activity and physical fitness are closely related, they represent distinct constructs; therefore, caution is needed when interpreting evidence derived from physical activity in the context of physical fitness assessment.

Emerging evidence suggests that interventions specifically targeting PF may yield more consistent and clinically meaningful benefits than those focused solely on physical activity behavior, particularly given the physiological impairments induced by cancer treatment [14,15]. PF reflects functional capacity and is directly affected by surgery, chemotherapy, and radiation, which can lead to declines in muscular strength, cardiorespiratory function, and body composition [16,17,18]. Accordingly, interventions designed to improve PF are well positioned to mitigate these deficits and support recovery [19,20].

Despite this growing body of evidence, methods used to assess PF in interventional studies remain heterogeneous and fragmented. Substantial variability exists in test selection, measurement protocols, and the timing of assessments relative to treatment phases, limiting comparability across studies and hindering clinical interpretation. Although prior reviews have examined the effects of exercise and rehabilitation interventions, few have systematically characterized PF assessment methods, particularly in relation to treatment phase and testing approaches [21,22,23]. This lack of standardization represents a key methodological gap and an area of ongoing debate within the field.

Given the prognostic importance of PF and the variability in its assessment, a comprehensive mapping of PF measurement approaches is warranted. A scoping review is well suited to this objective, as it enables systematic identification and characterization of methodological diversity rather than evaluation of intervention efficacy. Such an approach can clarify current practices, identify inconsistencies, and inform the development of standardized outcome measures.

Therefore, this study aimed to comprehensively map objective PF assessments used in interventional studies involving breast cancer survivors. Specifically, we sought to identify the PF components evaluated, summarize the types and characteristics of assessment tools, and highlight methodological gaps. We further aimed to inform the development of standardized, feasible, and clinically relevant PF assessment strategies to improve comparability across studies and support evidence-based survivorship care.

## 2. Methods

This scoping review was conducted in accordance with the Preferred Reporting Items for Systematic Review and Meta-Analyses extension for Scoping Reviews (PRISMA-ScR) guidelines (Supplementary Table S1 [24]) to ensure methodological rigor, transparency, and reproducibility. Registration information is not applicable.

### 2.1. Search Strategy and Eligibility Criteria

A comprehensive literature search was performed across primary bibliographic databases and supplementary sources. The primary bibliographic databases included PubMed, MEDLINE, EMBASE, Web of Science, KoreaMed, and KCI. Google Scholar and ClinicalTrials.gov were additionally searched as supplementary sources to identify potentially relevant articles, registered studies, or linked publications not captured through the primary databases. Studies published up to 30 November 2024 were considered. The search strategy combined controlled vocabulary and free-text terms related to breast cancer survivorship, physical fitness, and intervention studies. Search syntax was adapted to the indexing system and interface of each database, and database-specific search strategies for the primary bibliographic database, including searched fields and search structures, are provided in Supplementary Tables S2 and S3. Where applicable, controlled vocabulary terms were combined with title-, abstract-, and keyword-based searching. Language restrictions were applied at the eligibility stage, and only full-text articles published in English or Korean were considered for inclusion.

Because of its limited reproducibility, Google Scholar was used only as a supplementary relevance-based search source to identify potentially relevant articles not captured through the primary bibliographic databases; it was not treated as a primary reproducible database search. ClinicalTrials.gov was used only as a supplementary registry source to identify potentially relevant registered interventional studies and, where possible, corresponding peer-reviewed full-text journal publications; registry records themselves were not eligible for final inclusion unless a related eligible full-text journal article could be identified. Forward and backward citation tracking was not performed as a separate search step. Duplicate records were removed using EndNote-assisted deduplication, followed by manual verification based on citation details such as title, author, publication year, and DOI before title and abstract screening in Covidence.

Studies were included if they met the following criteria: (1) involved adult female breast cancer survivors (aged ≥19 years) who were either undergoing active treatment or had completed primary treatment (e.g., surgery, chemotherapy, or radiation therapy); (2) evaluated any type of intervention, including structured exercise programs, physical rehabilitation, or nutrition-related interventions; (3) reported at least one objectively measured PF outcome (e.g., cardiorespiratory fitness, grip strength, muscular strength, and flexibility tests); and (4) were available as full-text articles published in English or Korean. Because this scoping review synthesized peer-reviewed full-text journal articles, trial registry records were not eligible for final inclusion unless a corresponding eligible journal article could be identified. Studies were excluded if they: (1) were not original research (e.g., review articles, study protocols, conference abstracts, commentaries, or editorials); (2) assessed PF outcomes solely using self-reported methods (e.g., questionnaires) without objective measurements; or (3) were available only as abstracts and lacked sufficient methodological detail for evaluation.

Eligible interventions included any non-pharmacological intervention intended to modify physical fitness, functional capacity, body composition, or lymphatic function, encompassing (a) structured exercise programs (aerobic, resistance, combined, flexibility, balance, or high-intensity interval training), (b) physical rehabilitation interventions (including aqua lymphatic therapy, kinesiology taping, and gardening-based rehabilitation), and (c) lifestyle/nutrition interventions incorporating an objective PF outcome. Eligible study designs included randomized controlled trials, non-randomized controlled trials, quasi-experimental studies, controlled pre–post studies, and single-arm pre–post/feasibility/pilot studies reporting at least one objectively measured PF outcome before and after the intervention.

In this review, the term ‘breast cancer survivor’ is used in accordance with the National Cancer Institute Office of Cancer Survivorship definition, which considers an individual a cancer survivor from the time of diagnosis through the balance of life. This inclusive definition captures the full trajectory of physical fitness across pre-treatment, active-treatment, and post-treatment phases. During-treatment studies were retained because physical fitness during active therapy is clinically relevant to treatment tolerance, dose adherence, and short-term recovery, and is therefore a legitimate target of intervention research.

### 2.2. Unit of Analysis and Denominator Definition

**Figure 1:**
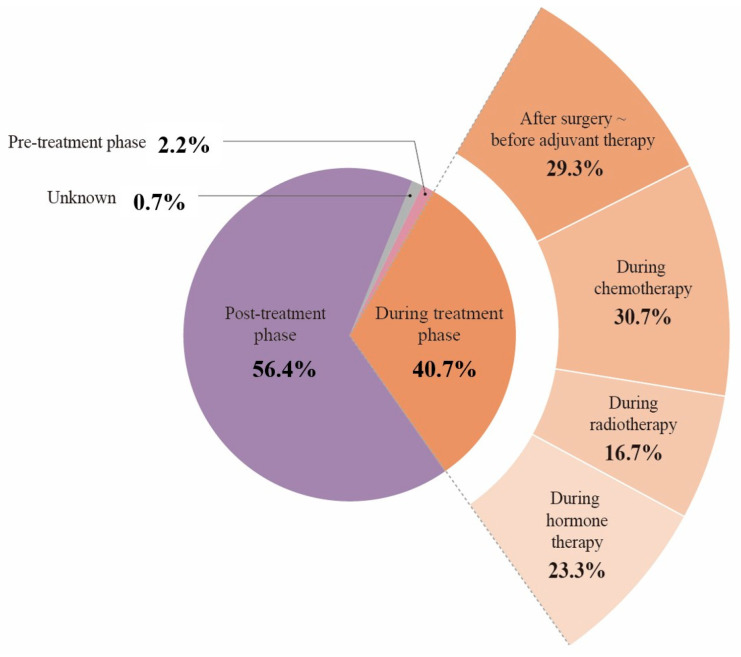
Distribution of treatment phase and treatment-context records represented in the included studies. The unit of analysis was the treatment phase/context record, not the unique study. The 316 included studies contributed 369 records because some studies included more than one treatment phase or treatment context. Percentages in the main chart were calculated using 369 records as the denominator: pre-treatment phase, *n* = 8; during treatment phase, *n* = 150; post-treatment phase, *n* = 208, and unknown, *n* = 3. The expanded panel shows the distribution of 150 during-treatment context records: post-surgical pre-adjuvant period, *n* = 44, chemotherapy, *n* = 46, radiation therapy, *n* = 25, and hormone/endocrine therapy, *n* = 35.

The primary unit of analysis for study selection and general study characteristics was the unique study. Thus, the final review included 316 unique studies. For the treatment phase analysis shown in Figure 1, the unit of analysis was the treatment-phase/context record. A treatment phase/context record was defined as one treatment phase or treatment context represented within one included study. A single study could contribute more than one record when it included participants across multiple treatment phases or treatment contexts, such as chemotherapy and radiotherapy. For example, a study including participants during both chemotherapy and radiotherapy contributed one record to the chemotherapy category and one record to the radiotherapy category. Accordingly, the 316 included studies contributed 369 treatment-phase/context records. Percentages in Figure 1 were calculated using these 369 records as the denominator, whereas the “expanded during treatment” panel used 150 during-treatment context records as the denominator.

For the supplementary study-level analysis, a study by domain binary matrix was constructed, in which each unique study was coded as 1 if a given physical fitness domain was assessed at least once and 0 otherwise. A single study could contribute to more than one domain; the resulting study-level counts and percentages are presented with Supplementary Table S10.

For analysis of PF domain, the unit of analysis was the PF domain record. A PF domain record was defined as one objectively measured PF domain reported within one included study. Because a single study could report more than one PF domain, the total number of PF domain records exceeded the number of included studies. Accordingly, the 316 included studies contributed 557 PF domain records, and percentages for PF domain distribution were calculated using these 557 records as the denominator.

For analyses of assessment method within each PF domain, each distinct test or protocol reported within a study was counted as an assessment method record. Therefore, denominators differed across domain-specific assessment method analyses and were explicitly stated in the corresponding figure caption.

### 2.3. Data Extraction and Charting

**Figure 2:**
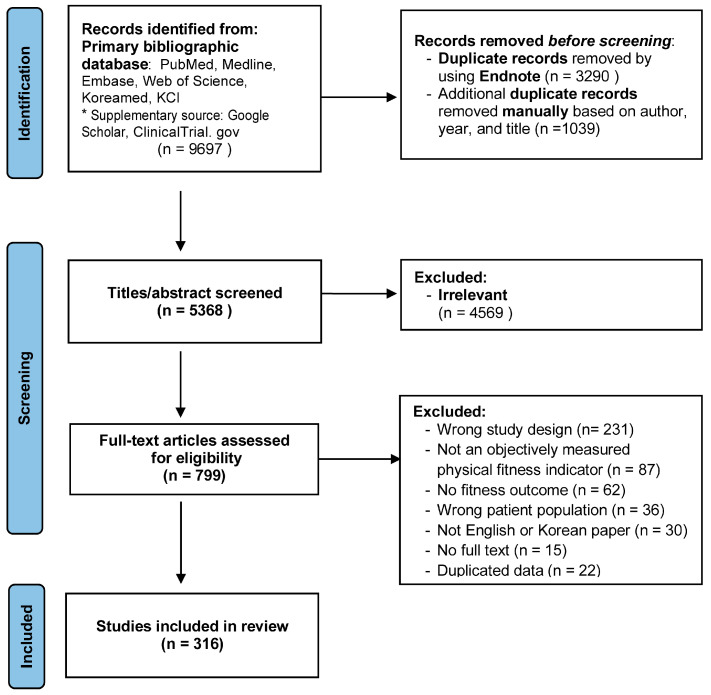
PRISMA flow diagram of study selection. * Supplementary source, including Google Scholar, ClinicalTrial.gov supported the identification of the literature search.

All identified records were imported into Covidence systematic review software (Veritas Health Innovation, Melbourne, Australia) for screening and management. Two reviewers (MO and JM) independently screened titles and abstracts, followed by full-text assessment based on the predefined eligibility criteria. Discrepancies were resolved through discussion until consensus was reached. The study selection process is illustrated in the PRISMA flow diagram (Figure 2). Data were extracted using a standardized form, including: (1) general study characteristics (author, publication year); (2) participant characteristics (sample size, mean age, mean body mass index, and treatment phase); (3) geographic distribution (country), which was classified based on the country affiliation of the first author, as reported in each publication; (4) intervention characteristics (type, frequency, duration, and delivery method); and (5) PF outcomes (fitness domains assessed, measurement methods, and assessment tools), along with key findings related to PF. No a priori classification of assessment methods based on measurement quality (e.g., “gold standard”) was applied during data extraction or analysis. The data extraction focused on identifying the types and characteristics of PF assessment methods and did not include detailed evaluation of measurement properties such as validity or reliability. Data extraction was conducted independently by two reviewers, and all entries were cross-checked for accuracy and completeness. Data synthesis and charting were performed by two co-authors using Microsoft Excel (Microsoft Corporation, Redmond, WA, USA).

### 2.4. Conceptual Framework and Classification of PF Domains

In this review, physical fitness is conceptualized following the American College of Sports Medicine taxonomy [25] as a set of attributes relating to the ability to perform physical activity, comprising (i) health-related components (cardiorespiratory fitness, muscular strength, muscular endurance, flexibility, body composition) and (ii) performance-related/functional components (balance, agility, gait speed, and composite functional performance tests such as the Short Physical Performance Battery and the Timed Up and Go). Physical function is therefore reported as a distinct domain.

#### Classification Rules for Ambiguous PF Tests

Ambiguous tests were classified according to their primary physiological construct and reported usage, and each test was assigned to a single category to avoid overlap. The 6-min walk test was classified as a walking test within cardiorespiratory fitness; sit-to-stand tests were classified within muscular strength (functional/performance subcategory), except when reported as part of the Short Physical Performance Battery; Timed Up and Go and Short Physical Performance Battery were classified within physical function.

## 3. Results

### 3.1. Study Selection

A total of 9697 records were identified, of which 4329 duplicates were removed. The remaining 5368 records underwent title and abstract screening, and 799 full-text articles were assessed for eligibility. Ultimately, 316 studies met the inclusion criteria (Figure 2). Detailed study characteristics are presented in Supplementary Tables S4–S8. Supplementary Tables S4–S8 present the classification of included studies according to breast cancer treatment phase (pretreatment, during treatment, post-treatment, unknown, and mixed phases) and summarize the corresponding study distributions within each category. Additionally, a detailed breakdown of the 231 full-text articles excluded due to ineligible study design is provided in Supplementary Table S9.

### 3.2. Characteristics of Included Studies

The descriptive characteristics of the included studies are summarized in Table 1. The 316 studies spanned a broad publication period, with over half (52.5%) published between 2011 and 2020 and nearly one-third published more recently, reflecting increasing research interest in this field. Small and moderately sized studies predominated, with 26.9% enrolling fewer than 30 participants and 33.5% enrolling 30–50 participants. By the region of the first author’s country affiliation, North America accounted for the largest proportion of studies (36.6%), followed by Europe (29.8%) and East Asia (13.0%). Most studies included participants in their fifties (39.9% aged 50–55 years and 27.2% aged over 55 years), although 17.7% did not report mean age.

Exercise-based interventions dominated the literature (83.2%), whereas non-exercise approaches, such as nutritional or lifestyle interventions, were less frequent (15.5%). Intervention delivery formats varied, with most conducted in home-based settings (57.8%), followed by supervised (17.8%) and combined delivery approaches (21.1%). Intervention duration most frequently ranged from 12 to 15 weeks (36.4%), although shorter (<6 weeks; 8.9%) and longer (>24 weeks; 8.2%) interventions were also represented. Collectively, these findings indicate a predominance of structured, moderate-duration interventions.

### 3.3. Distribution of Treatment Phase and Treatment Context Records

As shown in Figure 1, treatment phase was analyzed using treatment phase/context records rather than unique studies. The 316 included studies contributed 369 treatment-phase/context records because some studies included participants across more than one treatment-phase or treatment context. Mixed-phase studies (*n* = 9) contributed one record to each applicable treatment phase or context; therefore, Figure 1 denominator represents treatment phase/context records rather than mutually exclusive unique studies. Among these records, post-treatment phase records were most frequent (*n* = 208, 56.4%), followed by during treatment-phase/context records (*n* = 150, 40.7%), pre-treatment phase records (*n* = 8, 2.2%), and records with unknown treatment phase (*n* = 3, 0.8%).

Among the 150 during-treatment context records, interventions were most often delivered alongside chemotherapy (*n* = 46, 30.7%) or during the immediate post-surgical, pre-adjuvant period (*n* = 44, 29.3%), followed by hormone/endocrine therapy (*n* = 35, 23.3%), and radiotherapy (*n* = 25, 16.7%). Of the 316 included studies, 307 were categorized into a single treatment phase, whereas 9 studies (2.8%) were classified as mixed-phase and analyzed separately. These findings indicate that PF assessments were most represented in post-treatment and active treatment contexts.

Detailed classifications and study-level information are provided in Supplementary Tables S4–S8 and Figures S1–S3. Across the included studies, the distribution of intervention phases was as follows: 5 (1.6%) studies in pretreatment phase, 101 (32.0%) studies in during treatment phase, 199 (63.0%) studies in post-treatment phase, 2 studies did not specify the treatment phase, and 9 (2.8%) studies in mixed phases (Supplementary Tables S4–S8). To facilitate interpretation, Supplementary Table S11 presents the study-level distribution based on 316 mutually exclusive studies alongside the record-level phase/context distribution based on 369 treatment phase/context records. Supplementary Figures S1–S3 further illustrate the distribution of studies across treatment phases, including stratifications by study characteristics (e.g., intervention type, intervention duration, or outcome domain), providing a visual overview of how phase-specific patterns vary across the included literature.

### 3.4. Distribution of PF Domain Records and Assessment Methods

**Figure 3:**
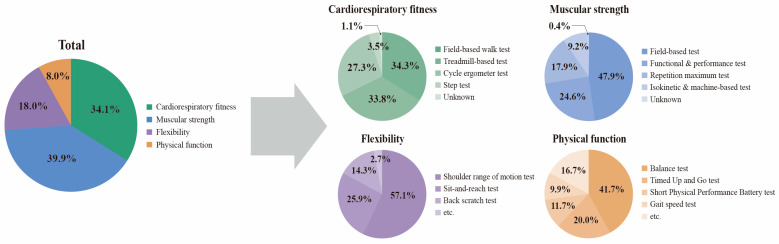
Distribution of physical fitness domain records and assessment methods. The “Total” pie chart presents PF domain records. A PF domain record is defined as one objectively measured PF domain reported within one included study; therefore, a single study could contribute more than one PF domain record. Across the 316 included studies, 557 PF domain records were identified. Percentages in the “Total” pie chart were calculated using 557 PF domain records as the denominator. The four pie charts on the right present assessment-method records within each PF domain. Percentages in each PF domain were calculated using domain-specific assessment method denominators: cardiorespiratory fitness, *n* = 198; muscular strength, *n* = 357; flexibility, *n* = 112; and physical function, *n* = 60. A single study could contribute more than one assessment-method record when multiple tests or protocols were reported within the same domain. Field-based tests were defined as practical assessments, such as walking test, grip strength, handheld dynamometry, or the arm curl test. The “etc.” (other) category for flexibility includes the trunk rotation test, side bend test, and hand-behind-back position, whereas the “other” category for physical function includes the Figure-3 turning test, Senior Fitness Test, 10-m shuttle run test, and whole-body reaction time test.

As illustrated in Figure 3, PF domain distribution was analyzed at the PF domain record level. Across the 316 included studies, 557 PF domain records were identified because a single study could report more than one objectively measured PF domain. Muscular strength accounted for the largest proportion of PF domain records (*n* = 222, 39.9%), followed by cardiorespiratory fitness (*n* = 190, 34.1%), flexibility (*n* = 100, 18.0%) and physical function (*n* = 45, 8.0%). To complement this record-level analysis, Supplementary Table S10 presents the number and percentage of unique studies (*n* = 316) that assessed each physical fitness do-main. In this dataset, the domain-specific study counts were numerically identical to the corresponding PF domain record counts, but percentages differed because study-level prevalence used 316 unique studies as the denominator, whereas record-level frequency used 557 PF domain records.

Assessment methods were analyzed separately within each PF domain using assessment method records as the denominator (Figure 3). For cardiorespiratory fitness, 198 assessment method records were identified. Field-based walking test (*n* = 68, 34.3%), treadmill-based test (*n* = 67, 33.8%), and cycle ergometer tests (*n* = 54, 27.3%) were the most common methods, followed by step test (*n* = 7, 3.5%), and unknown methods (*n* = 2, 1.0%).

For muscular strength, 357 assessment method records were identified. Field-based tests were the most frequent (*n* = 171, 47.9%), followed by functional or performance tests (*n* = 88, 24.6%), repetition maximum tests (*n* = 64, 17.9%), and isokinetic or machine-based protocols (*n* = 33, 9.2%), and unknown (*n* = 1, 0.3%). Flexibility assessments predominantly targeted the shoulder region, with shoulder range of motion (*n* = 64 of 112 records, 57.1%), followed by sit and reach test (*n* = 29, 25.9%), back stretch tests (*n* = 16, 14.3%), and other flexibility test (*n* = 3, 2.7%). Physical function outcomes were primarily measured using balance tests (*n* = 25 of 60 records, 41.7%), followed by the Timed Up and Go test (*n* = 12, 20.0%), other physical function test (*n* = 10, 16.7%), the Short Physical Performance Battery test (*n* = 7, 11.7%), and gait speed assessments (*n* = 6, 10.0%). Collectively, these findings indicate that muscular and cardiorespiratory domains have been prioritized, while physical function outcomes, which are relevant to daily living, remain comparatively underrepresented.

### 3.5. Distribution of Intervention Types by Breast Cancer Treatment Phase

Across all treatment phases, exercise-based interventions were more common than lifestyle or non-exercise programs (Supplementary Figure S1). Combined exercise programs (66.7%) were the predominant modality in pre-, during-, and post-treatment phases, followed by steady-state aerobic and resistance training interventions.

### 3.6. Distribution of Intervention Duration by Breast Cancer Treatment Phase

Across all treatment phases, intervention durations most commonly ranged from 12 to 15 weeks, comprising 25.0% of pre-treatment, 42.7% during-treatment, and 41.0% of post-treatment interventions (Supplementary Figure S2).

### 3.7. Distributions of Fitness Components by Breast Cancer Treatment Phase

Muscular strength was the most frequently assessed PF outcome across all treatment phases (Supplementary Figure S3). Cardiorespiratory fitness was the second most frequent, particularly during treatment (37.0%) and post-treatment (33.2%) phases. Conversely, flexibility assessments were relatively more frequent in pre-treatment (26.3%) compared to during-treatment (15.8%) and post-treatment (18.5%). Physical function measures were consistently the least reported across all phases.

## 4. Discussion

This scoping review provides a comprehensive synthesis of the utilization of objective PF measures in interventional studies involving breast cancer survivors. Across 316 studies, we identified substantial heterogeneity in the timing, methods, and scope of PF assessments. Muscular strength and cardiorespiratory fitness were the most frequently evaluated domains, whereas flexibility and physical function were comparatively underrepresented. Additionally, most assessments were conducted after the completion of primary treatment, predominantly within moderate duration (12–15 weeks), exercise-based interventions.

The increasing emphasis on PF assessment reflects its recognized clinical importance in breast cancer survivorship. Survivors frequently experience persistent impairments as a result of surgery, chemotherapy, radiation, and hormone therapy, leading to declines in muscular strength, cardiorespiratory capacity, and functional mobility [16,17,18,26]. These impairments are associated with reduced quality of life, lower treatment tolerance, increased fatigue, and poorer survival outcomes [27,28,29]. Our findings further indicate that PF assessments are unevenly distributed across treatment phases, with a predominant focus on the post-treatment period and minimal assessment prior to treatment initiation (only 2.2% identified). This imbalance likely reflects both clinical and practical considerations. During active treatment, adverse effects such as fatigue, nausea, neuropathy and immunosuppression may limit the feasibility and prior PF testing. Clinical attention is often directed toward treatment safety and tolerance, reducing emphasis on fitness assessment. Similarly, pre-treatment assessments are underutilized, likely due to the limited time between diagnosis and treatment initiation, and the psychological burden experienced by patients during this period [30,31,32]. The limited number of pretreatment studies identified in this review highlights an important gap in the current evidence base. Future research is warranted to determine whether PF assessment during the pre-treatment phase may have value for risk stratification, prehabilitation planning, and longitudinal monitoring.

Although an inclusive definition of survivorship was adopted in this review, it is important to recognize that individuals undergoing active treatment and those in the post-treatment phase may differ in their PF profiles, symptom burden, and functional capacity. These differences may influence both the selection and feasibility of PF assessments and should be considered when interpreting the observed heterogeneity across studies.

In contrast, the post-treatment phase is more conducive to PF assessment, as patients are generally more stable and able to participate in testing, and clinicians may prioritize rehabilitation planning. However, this timing is not merely logistical; PF levels and their relationship with clinical outcomes vary across treatment phases. For instance, Irwin et al. [33] observed that physical activity declines abruptly during chemotherapy (~50%) and radiotherapy (~24%), suggesting that PF testing during treatment may not accurately represent survivors’ true physical capacity. A meta-analysis study by Juvet et al. [34] found that improvements in physical function and fatigue were more pronounced when exercise interventions were implemented after the completion of adjuvant therapy. Similarly, a randomized controlled trial by Møller et al. [16] reported that early post-treatment interventions could effectively restore cardiorespiratory fitness as well as alleviate fatigue and pain more effectively than interventions initiated during treatment. For breast cancer, this implies that PF measurements across treatment phases may reflect different physiological states; however, further research is required to determine optimal phase-specific assessment approaches.

Additionally, evidence from other types of cancer highlights that the physiological profile of cancer survivors, and the relationship between PF and health outcomes, can vary depending on the timing and sequencing of treatments. For instance, in a prostate cancer cohort [35], vigorous or increasing physical activity post-treatment conferred survival benefits among patients treated with surgery alone, but such benefits were attenuated or absent in those who had also received adjuvant therapies. The distinction between physical activity and PF should be considered when interpreting these findings, as evidence derived from physical activity may not directly reflect changes in PF. Although declines in physical activity during treatment have been reported, these findings should not be interpreted as evidence of reduced validity of PF assessment. Rather, PF performance during treatment may fluctuate substantially, which may influence the timing, feasibility, and interpretation of assessments.

The importance of incorporating objective PF measures into interventional research lies in their ability to capture significant changes in physical capacity that may not be reflected in self-reported outcomes alone. Unlike physical activity behavior, which is influenced by motivation, environment, and self-perception, PF reflects biological and functional adaptations to exercise stimuli [36,37]. For instance, increases in cardiorespiratory fitness or muscular strength represent clinically relevant improvements that may reduce the risk of cancer recurrence, delay disease progression, or mitigate treatment-related adverse effects [37,38,39,40]. Furthermore, evidence suggests that higher levels of PF at diagnosis or during treatment are associated with better outcomes, including fewer complications and reduced mortality [37,41].

Despite these advantages, this review identified substantial variability in the types and characteristics of PF assessment methods used across studies. However, any interpretation regarding measurement properties, such as validity, reliability, and sensitivity, should be understood as being informed by prior measurement literature rather than directly derived from the present descriptive mapping. While some studies employed laboratory-based methods, such as cardiopulmonary exercise testing with direct gas exchange analysis for cardiorespiratory fitness [16,42,43,44,45,46] or isokinetic dynamometry for muscular strength [47,48,49,50,51], many relied on field-based or submaximal methods, including walk tests [52,53,54,55,56], the Short Physical Performance Battery [53,54,56], or submaximal cycle ergometry without oxygen uptake measurement [57,58,59,60,61]. Although these tools are practical and widely used, prior measurement literature suggests that some may be subject to ceiling effects [62,63], measurement error [64], and reduced sensitivity [65] when detecting small but clinically meaningful changes. It should be noted that measurement properties such as validity, reliability, and sensitivity were not systematically extracted in this review, and the above considerations are based on existing literature rather than findings from the present analysis. In breast cancer studies, the minimal clinically important difference for the 6-min walk test indicates that small improvements may fall within measurement error, suggesting that selection of assessment methods should consider study objectives and measurement context when detecting subtle yet clinically meaningful changes is a priority [65]. Similarly, muscular strength was often assessed using handgrip dynamometry or one-repetition maximum testing, which have demonstrated moderate-to-high reliability but may capture different aspects of physical performance compared with multi-joint or isokinetic measures [66,67,68]. Flexibility assessments also varied, with some using standardized goniometric measurements while others employed less standardized approaches, such as functional reach or non-validated movement screens, potentially reducing comparability [53,69,70,71,72,73,74]. This variability in assessment approaches echoes findings in other cancer populations, such as pediatric cancer survivors [75,76] and patients with metastatic breast cancer [77,78], where variability in PF measures has been identified as a barrier to quantitative synthesis and guideline development. Several commonly used tools, including the 6-min walk test, Short Physical Performance Battery, handgrip dynamometry, and the Senior Fitness Test, have demonstrated strong reliability and validity, along with established clinical cut-points for predicting morbidity, disability, and mortality [53,79,80,81,82]. When applied appropriately, these measures offer practicality and clinical relevance. Future research should aim to evaluate and identify practical and context-appropriate PF measures, including their measurement properties and potential applicability in cancer populations. Moreover, flexibility and physical function domains remain underrepresented among PF outcomes in the included studies, despite their clear relevance to daily living and rehabilitation. Flexibility assessments, such as shoulder range of motion and sit-and-reach tests, may be particularly important for survivors who have undergone mastectomy or axillary surgery, given the associated risk of shoulder dysfunction and lymphedema [83,84]. Similarly, assessments of functional mobility, such as the Timed Up and Go or balance tests, are especially relevant for older survivors or those with comorbidities, and may be considered for inclusion in future study protocols [85,86,87].

This review also highlights variability in intervention approaches, including the use of home-based and combined exercise interventions; however, temporal trends were not formally analyzed and should be interpreted with caution. This is particularly relevant in the context of expanding digital health tools and the growing need for accessible, scalable rehabilitation strategies. However, as remote and unsupervised assessments become more common, future studies should explicitly examine and report their validity and reliability, and feasibility, particularly for PF measures that require technical precision or standardized equipment.

Most interventional studies examined midlife patients, with relatively few studies enrolling younger (<50 years) or older (e.g., ≥65–70 years) survivors, and age-stratified analyses were limited. Therefore, findings from the present review do not allow for conclusions regarding age-specific differences in PF assessment. However, age-related considerations may be important and warrant further investigation in future studies. Given that PF trajectories, safety considerations, and test responsiveness may differ across age groups, future research could explore age-specific PF assessment approaches and report age-stratified outcomes to improve clinical relevance. A life-course approach is therefore warranted: leveraging pre-/early post-treatment windows in younger cancer survivors to prevent long-term deconditioning [88], while prioritizing functional independence, prognosis, and quality of life-relevant endpoints (along with longer follow-up) in older survivors [89].

A key implication of this review is that, in breast cancer survivorship, PF assessment should not be viewed as a generic exercise research outcome alone. Unlike non-cancer populations, breast cancer survivors present with treatment-specific impairments, including upper-extremity dysfunction, lymphedema risk, cancer-related fatigue, cardiorespiratory decline, and functional vulnerability. Therefore, inconsistent PF assessment may limit cross-study comparability and may affect the interpretation of intervention relevance.

This scoping review has several strengths, including a comprehensive multi-database search with dual independent screening, adherence to PRISMA-ScR guidelines, and the largest phase-stratified mapping (*n* = 316) of objective PF measures in breast cancer interventions to date. Nevertheless, some limitations should be acknowledged. First, heterogeneity in PF measures and assessment procedures across studies constrains comparability and, as observed in other oncology populations, hinders the development of standardized guidance. Second, external validity may be limited by the predominance of single-center studies and inconsistent reporting or validation of measures in specific subgroups. Third, consistent with scoping review methodology, no formal study-level risk-of-bias assessment was conducted; the emphasis was on breadth and gap identification rather than critical appraisal. Fourth, because only English and Korean full-text articles were included, studies published in other languages may have been underrepresented, particularly from settings in which local-language publication is more common. Fifth, another key limitation of this review is that detailed information on test protocols, measurement timing, device specifications, and validation status was not systematically extracted. Therefore, our findings do not allow for a comprehensive evaluation of the validity, reliability, or clinical applicability of the included assessment methods. While variability in assessment methods was observed across studies, these findings should be interpreted as descriptive rather than evaluative, as the present review was not designed to formally assess measurement quality or establish standardized protocols. Future research is warranted to systematically evaluate the measurement properties of commonly used PF assessments, including their validity, reliability, and clinical utility, and to support the development of standardized assessment frameworks. Sixth, another limitation of this review is that studies were not systematically analyzed according to contextual factors such as geographic region, healthcare setting, disease stage, or treatment regimen. Additionally, it should be noted that geographic classification was based on the first author’s institutional affiliation rather than the actual participant recruitment location. As such, this variable may not fully reflect the underlying healthcare context or study population, particularly in multicenter or international studies. As these factors may influence the selection and feasibility of PF assessment methods, the present findings should be interpreted as general patterns rather than context-specific trends. Future research is warranted to investigate how PF assessment approaches vary across different regions, treatment phases, and intervention types to better inform context-specific applications. Seventh, an additional source of heterogeneity that was not systematically examined in this review is study design. Although a range of eligible study designs was allowed, study design was not prospectively coded and harmonized as a stratification variable during data charting. Therefore, we were unable to provide a reliable design-stratified summary of PF assessment patterns across randomized controlled trials, pilot studies, feasibility studies, and single-arm studies, which may influence both outcome selection and measurement approaches. As a result, the present findings should be interpreted as general patterns across studies rather than design-specific trends. Future research is warranted to explore how PF assessment strategies vary according to study design. Finally, reliance on diverse, non-standardized PF assessments limits cross-study comparisons and meta-analysis, reinforcing the need for harmonized reporting and future evaluation of assessment protocols with documented measurement properties, as highlighted in prior scoping work.

## 5. Conclusions

PF is a central but inconsistently assessed domain in breast cancer survivorship research. This review provides an overview of the distribution and characteristics of objective PF assessment methods across studies. Objective assessment of PF may be important for helping future intervention studies capture physiological responses more directly than self-report alone. Future studies may consider employing clearly defined, standardized, and context-appropriate PF assessment methods, including participants across all treatment phases and age groups, and ensuring sufficient follow-up to evaluate sustained effects. Future intervention studies would benefit from more standardized reporting of PF assessments, including clear descriptions of test names, protocol details, timing relative to treatment phase, device specifications, assessor training, and repeated-measure schedules. More consistent reporting of these methodological elements may improve comparability across studies and facilitate future evidence synthesis. Establishing consensus on core PF outcomes and assessment approaches, alongside future evaluation and selection of tools with documented measurement properties, may improve comparability across studies and inform the development of more consistent assessment frameworks for breast cancer survivorship, including rehabilitation planning, symptom management, functional recovery, and long-term outcomes. Given that no formal study-level risk-of-bias assessment was conducted, the findings of this review should be interpreted as descriptive rather than evaluative.

## Figures and Tables

**Table 1:** Characteristics of included studies (*n* = 316).

Characteristics	*n*	%
**Publication year**		
<2000	1	0.3
2000–2010	53	16.8
2011–2020	166	52.5
>2020	96	30.4
**Sample size (N of study participants)**		
<30	85	26.9
30–50	106	33.5
>50	125	39.6
**Country**		
North America	116	36.6
Europe	94	29.8
East Asia	41	13.0
West/Middle Asia	22	6.9
Latin America/Caribbean	19	6.1
Oceania	12	3.8
Africa	12	3.8
**Mean age of study participants**		
<50 years	48	15.2
50–55 years	126	39.9
>55 years	86	27.2
Not reported	56	17.7
**Intervention type**		
Exercise intervention	263	83.2
Non-exercise intervention *	49	15.5
Unknown	4	1.3
**Intervention delivery type**		
Home-based intervention	183	57.8
Supervised intervention	56	17.8
Combined intervention	67	21.1
Unknown	10	3.3
**Intervention duration**		
<6 weeks	28	8.9
6–11 weeks	79	25.0
12–15 weeks	115	36.4
16–24 weeks	62	19.6
>24 weeks	26	8.2
Unknown	6	1.9

Note: Data are presented as numbers and percentages. * Aqua lymphatic therapy, nutritional support, gardening, or kinesiology taping. Geographic classification (country) is based on the first author’s institutional affiliation.

## Data Availability

The original contributions presented in this study are included in the article/Supplementary Materials. Further inquiries can be directed to the corresponding author.

## Supplementary Materials

The following supporting information can be downloaded at: https://www.mdpi.com/article/10.3390/cancers18101642/s1, Figure S1. Distribution of intervention type by breast cancer treatment phase; Figure S2. Distribution of intervention duration by breast cancer treatment phase; Figure S3. Distribution of physical fitness components by breast cancer treatment phase; Table S1. Preferred Reporting Items for Systematic reviews and Meta-Analyses extension for Scoping Reviews (PRISMA-ScR) Checklist; Table S2. Search Strategy; Table S3. Search Strategy; Table S4 Studies Categorized by Phase: Pretreatment Phase (N = 5); Table S5. Studies Categorized by Phase: During Phase (N = 101); Table S6. Studies Categorized by Phase: Post-treatment Phase (N = 199); Table S7. Studies Categorized by Phase: Unknown (N = 2); Table S8. Studies Categorized by Phase: Mixed Phases (N = 9); Table S9. Detailed breakdown of the 231 full-text articles excluded due to ineligible study design; Table S10. Study-level prevalence and record-level frequency of physical fitness domains; Table S11. Comparison of study-level phase distribution and record-level phase/context distribution.

## Abbreviations

The following abbreviations are used in this manuscript:

PF	Physical Fitness
PRISMA-ScR	Preferred Reporting Items for Systematic Reviews and Meta-Analyses Extension for Scoping Reviews
