# Supplementary material for: Mapping Physical Fitness Assessments in Interventional Research Among Breast Cancer Survivors: A Scoping Review
Source: Cancers (Basel). 2026 May 19;18(10):1642. doi: 10.3390/cancers18101642 (PMC13204595; doi:10.3390/cancers18101642)
Supplement: Supplementary file 1 [file cancers-18-01642-s001.zip › cancers-4272201-supplementary.pdf]

cancers MDPI

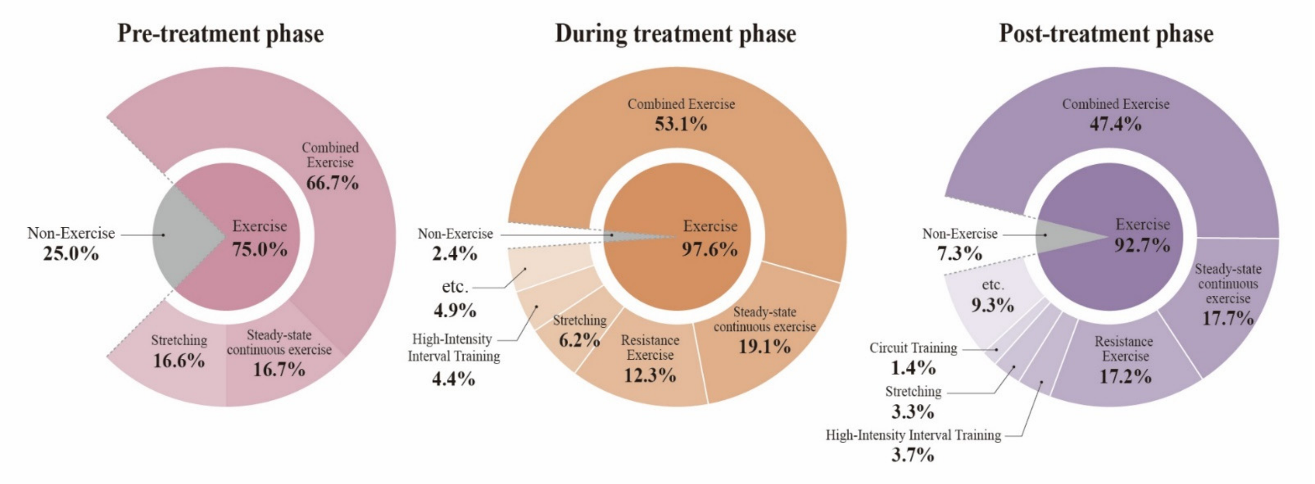

Figure S1. Distribution of intervention type by breast cancer treatment phase. Distribution of exercise and non-exercise interventions. Combined exercise intervention is defined as programs integrating two or more exercise modalities.

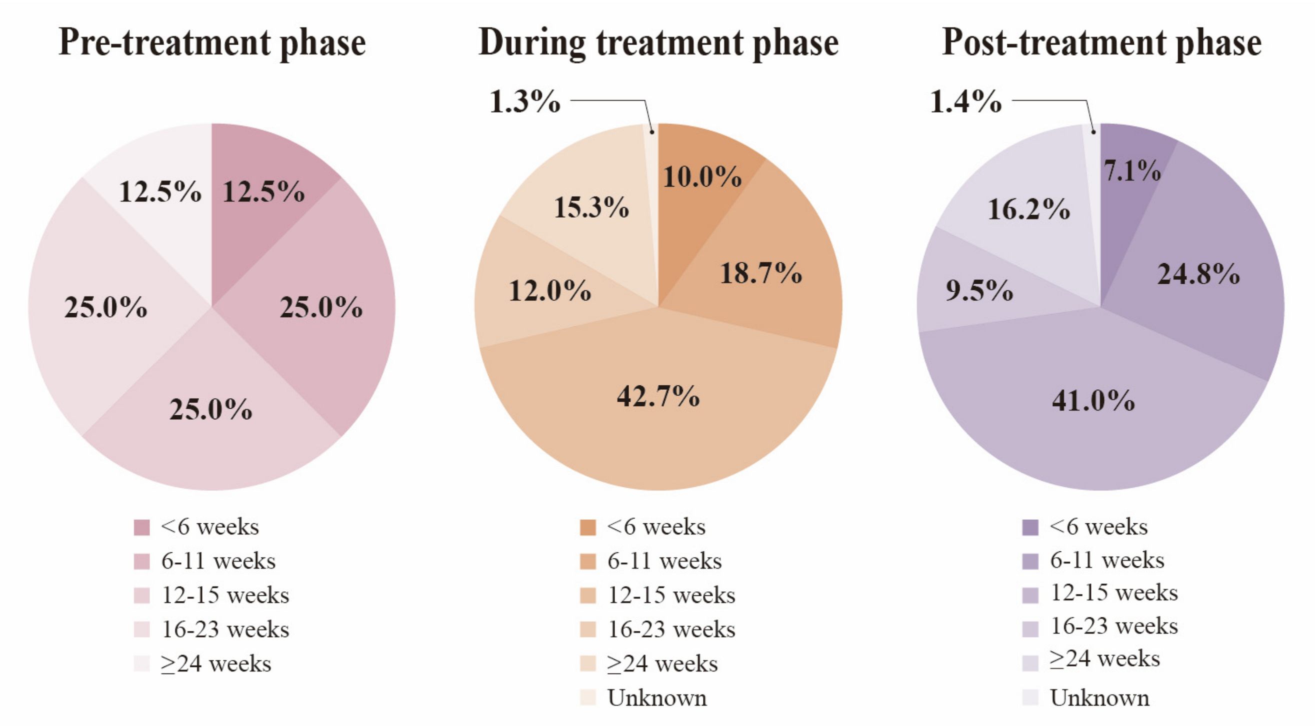

Figure S2. Distribution of intervention duration by breast cancer treatment phase.

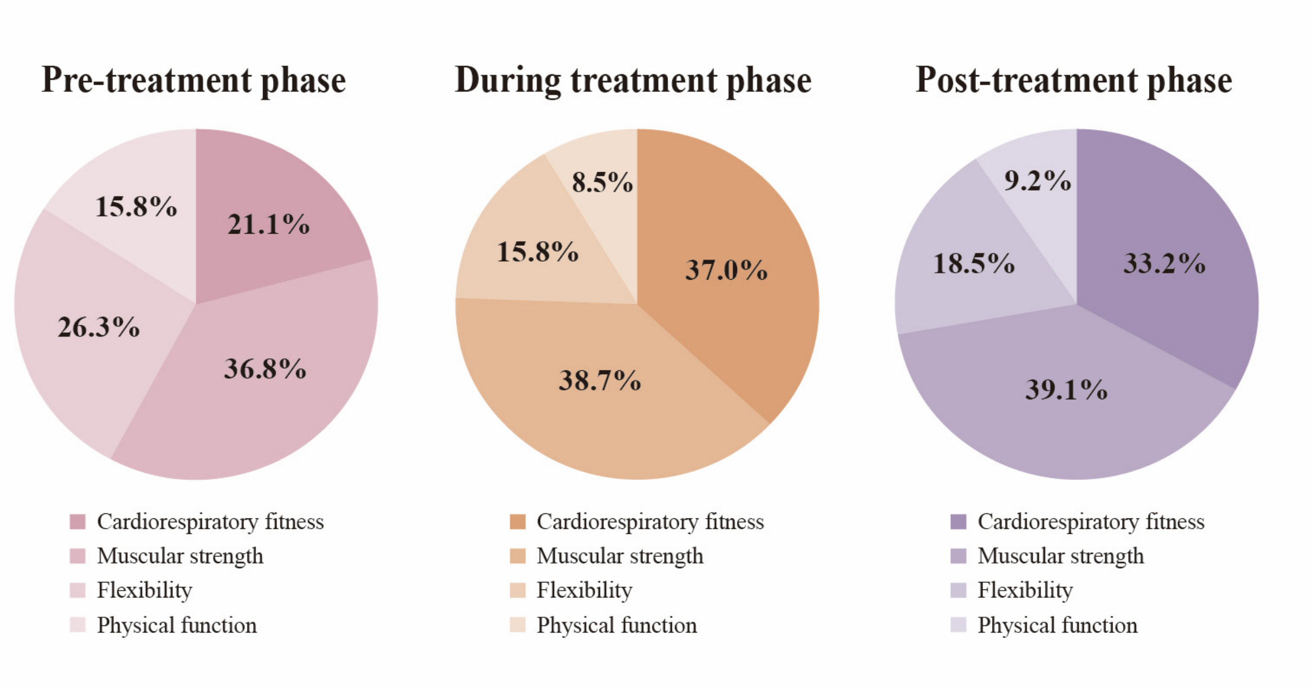

Figure S3. Distribution of physical fitness components by breast cancer treatment phase. Physical function tests were defined as measures of functional mobility and balance (e.g., balance test, timed Up and Go), reflecting performance-based indicators of stability and movement.

Table S1. Preferred Reporting Items for Systematic reviews and Meta-Analyses extension for Scoping Reviews (PRISMA-ScR) Checklist.

SECTION	ITEM	PRISMA-ScR CHECKLIST ITEM	REPORTED ON PAGE #
		**TITLE**	
Title	1	Identify the report as a scoping review.	1
		**ABSTRACT**	
Structured summary	2	Provide a structured summary that includes (as applicable): background, objectives, eligibility criteria, sources of evidence, charting methods, results, and conclusions that relate to the review questions and objectives.	1-2
		**INTRODUCTION**	
Rationale	3	Describe the rationale for the review in the context of what is already known. Explain why the review questions/objectives lend themselves to a scoping review approach.	2
Objectives	4	Provide an explicit statement of the questions and objectives being addressed with reference to their key elements (e.g., population or participants, concepts, and context) or other relevant key elements used to conceptualize the review questions and/or objectives.	2-3
		**METHODS**	
Protocol and registration	5	Indicate whether a review protocol exists; state if and where it can be accessed (e.g., a Web address); and if available, provide registration information, including the registration number.	3
Eligibility criteria	6	Specify characteristics of the sources of evidence used as eligibility criteria (e.g., years considered, language, and publication status), and provide a rationale.	3-4
Information sources*	7	Describe all information sources in the search (e.g., databases with dates of coverage and contact with authors to identify additional sources), as well as the date the most recent search was executed.	3-4
Search	8	Present the full electronic search strategy for at least 1 database, including any limits used, such that it could be repeated.	3-4

SECTION	ITEM	PRISMA-ScR CHECKLIST ITEM	REPORTED ON PAGE #
Selection of sources of evidence†	9	State the process for selecting sources of evidence (i.e., screening and eligibility) included in the scoping review.	3-4
Data charting process‡	10	Describe the methods of charting data from the included sources of evidence (e.g., calibrated forms or forms that have been tested by the team before their use, and whether data charting was done independently or in duplicate) and any processes for obtaining and confirming data from investigators.	4-5
Data items	11	List and define all variables for which data were sought and any assumptions and simplifications made.	4
Critical appraisal of individual sources of evidence§	12	If done, provide a rationale for conducting a critical appraisal of included sources of evidence; describe the methods used and how this information was used in any data synthesis (if appropriate).	n/a
Synthesis of results	13	Describe the methods of handling and summarizing the data that were charted.	4
**RESULTS**			
Selection of sources of evidence	14	Give numbers of sources of evidence screened, assessed for eligibility, and included in the review, with reasons for exclusions at each stage, ideally using a flow diagram.	5
Characteristics of sources of evidence	15	For each source of evidence, present characteristics for which data were charted and provide the citations.	5-7
Critical appraisal within sources of evidence	16	If done, present data on critical appraisal of included sources of evidence (see item 12).	n/a
Results of individual sources of evidence	17	For each included source of evidence, present the relevant data that were charted that relate to the review questions and objectives.	5-7
Synthesis of results	18	Summarize and/or present the charting results as they relate to the review questions and objectives.	5-7
**DISCUSSION**			
Summary of evidence	19	Summarize the main results (including an overview of concepts, themes, and types of evidence available), link to the review questions and objectives, and consider the relevance to key groups.	7-10
Limitations	20	Discuss the limitations of the scoping review process.	10
Conclusions	21	Provide a general interpretation of the results with respect to the review questions and objectives, as well as potential implications and/or next steps.	10
**FUNDING**			
Funding	22	Describe sources of funding for the included sources of evidence, as well as sources of funding for the scoping review. Describe the role of the funders of the scoping review.	11

JBI = Joanna Briggs Institute; PRISMA-ScR = Preferred Reporting Items for Systematic reviews and Meta-Analyses extension for Scoping Reviews. * Where *sources of evidence* (see second footnote) are compiled from, such as bibliographic databases, social media platforms, and Web sites. † A more inclusive/heterogeneous term used to account for the different types of evidence or data sources (e.g., quantitative and/or qualitative research, expert opinion, and policy documents) that may be eligible in a scoping review as opposed to only studies. This is not to be confused with *information sources* (see first footnote). ‡ The frameworks by Arksey and O'Malley (6) and Levac and colleagues (7) and the JBI guidance (4, 5) refer to the process of data extraction in a scoping review as data charting. § The process of systematically examining research evidence to assess its validity, results, and relevance before using it to inform a decision. This term is used for items 12 and 19 instead of "risk of bias" (which is more applicable to systematic reviews of interventions) to include and acknowledge the various sources of evidence that may be used in a scoping review (e.g., quantitative and/or qualitative research, expert opinion, and policy document). *From:* Tricco AC, Lillie E, Zarin W, O'Brien KK, Colquhoun H, Levac D, et al. PRISMA Extension for Scoping Reviews (PRISMAScR): Checklist and Explanation. Ann Intern Med. 2018;169:467–473. doi: 10.7326/M18-0850.

Table S2. Search Strategy.

Source	Last Searched Date	Search Fields	Search Strategy Summary	Role In Review
PubMed	November 30, 2024,	MeSH, title/abstract	Combined terms for breast cancer survivorship, physical fitness, and intervention studies using controlled vocabulary and free-text term	Primary bibliographic data base
Medline		MeSH/ multipurpose field (.mp.)	Combined terms for breast cancer survivorship, physical fitness, and intervention studies using controlled vocabulary and free-text terms adapted to Ovid MEDLINE syntax.	
EMBASE		Emtree, title, abstract, keywords	Combined controlled vocabulary and free-text term adapted to EMBASED syntax	
Web of Science		Topic field (author, title, abstract, keywords)	Topic based search using terms related to breast cancer survivors, physical fitness, and intervention studies	
KoreaMed		Title, abstract, keywords	Korean-language search using terms equivalent to breast cancer, survivors, fitness, and intervention	
KCI		Title, abstract, keywords	Korean-language search adapted for local indexing and interface	
Google Scholar		Full-text relevance-based search engine retrieval	Supplementary search using simplified combinations of core terms; results were screened in relevance order	Supplementary source
ClinicalTrials.gov		Registry fields, keywords	Supplementary search to identify potentially relevant registered interventional studies and linked pulications	

Table S3. Search Strategy.

Source		Search Terminology	Results
PUBMED	1	"Breast Neoplasms"[Mesh] OR breast cancer*[tiab] OR breast neoplas*[tiab] OR breast tumor*[tiab] OR breast tumour*[tiab] OR breast malignan*[tiab] OR breast carcinoma*[tiab]	1,897
	2	"Physical Fitness"[Mesh] OR "Exercise Test"[Mesh] OR "Muscle Strength"[Mesh] OR "Exercise Therapy"[Mesh] OR "Rehabilitation"[Mesh] OR "Diet Therapy"[Mesh] OR "Life Style"[Mesh] OR physical fitness[tiab] OR cardiorespiratory fitness[tiab] OR aerobic capacity[tiab] OR maximal oxygen uptake[tiab] OR VO2max[tiab] OR treadmill test[tiab] OR step test[tiab] OR cycle ergometer[tiab] OR six-minute walk[tiab] OR 6-minute walk[tiab] OR two-minute walk[tiab] OR 2-minute walk[tiab] OR muscular strength[tiab] OR muscle power[tiab] OR handgrip strength[tiab] OR grip strength[tiab] OR dynamometer[tiab] OR one-repetition maximum[tiab] OR one repetition maximum[tiab] OR 1RM[tiab] OR push-up[tiab] OR sit-up[tiab] OR chair stand[tiab] OR arm curl[tiab] OR muscle endurance[tiab] OR flexibility[tiab] OR sit-and-reach[tiab] OR joint flexibility[tiab] OR shoulder range of motion[tiab] OR balance test[tiab] OR exercise intervention[tiab] OR training program[tiab] OR physiotherapy[tiab] OR physical activity program[tiab] OR resistance training[tiab] OR aerobic exercise[tiab] OR yoga[tiab] OR Pilates[tiab] OR Tai Chi[tiab] OR Qigong[tiab] OR home-based exercise[tiab] OR supervised exercise[tiab] OR combined exercise[tiab] OR gardening[tiab] OR lifestyle intervention[tiab] OR lifestyle modification[tiab] OR diet intervention[tiab] OR nutritional support[tiab] OR behavioral counseling[tiab]	
	3	"Survivors"[Mesh] OR survivor*[tiab] OR survivorship[tiab]	
	4	intervention*[tiab] OR trial*[tiab] OR experiment*[tiab] OR program*[tiab]	
	5	1 AND 2 AND 3 AND 4	
	6	5 NOT (animals[mh] NOT humans[mh])	
Medline (ovid)	1	exp Breast Neoplasms/ OR (breast adj2 (cancer* OR neoplas* OR tumo?r* OR malignan* OR carcinoma*)).mp.	1,897
	2	exp Physical Fitness/ OR (physical fitness OR cardiorespiratory fitness OR aerobic capacity OR maximal oxygen uptake OR VO2max OR treadmill test OR step test OR cycle ergometer OR six-minute walk OR two-minute walk OR muscular strength OR muscle power OR handgrip strength OR grip strength OR dynamometer OR one-repetition maximum OR push-up OR sit-up OR chair stand OR arm curl OR muscle endurance OR flexibility OR sit-and-reach OR joint flexibility OR shoulder range of motion OR balance test).mp.	
	3	exp Exercise Therapy/ OR exp Rehabilitation/ OR exp Diet Therapy/ OR exp Life Style/ OR exp Behavior Therapy/ OR (exercise intervention OR training program OR rehabilitation OR physiotherapy OR physical activity program OR resistance training OR aerobic exercise OR yoga OR Pilates OR Tai Chi OR Qigong OR home-based exercise OR supervised exercise OR combined exercise OR gardening OR lifestyle intervention OR lifestyle modification OR diet intervention OR nutritional support OR behavioral counseling).mp.	
	4	2 OR 3	

	5	1 AND 4	
	6	exp Survivors/ OR (survivor* OR survival* OR survivorship).mp.	
	7	5 AND 6	
	8	exp Intervention Studies/ OR (intervention* OR trial* OR experiment* OR program*).mp.	
	9	7 AND 8	
	10	exp animals/ NOT humans.sh.	
	11	9 NOT 10	
EMBASE	1	'breast tumor'/exp	3,325
	2	(breast NEAR/3 (cancer* OR carcinom* OR tumor* OR tumour* OR neoplasm* OR adeno*)):ti,ab,kw	
	3	#1 OR #2	
	4	'fitness'/exp	
	5	'physical fitness':ti,ab,kw OR 'cardiorespiratory fitness':ti,ab,kw OR 'treadmill test*':ti,ab,kw OR 'step test*':ti,ab,kw OR 'maximal oxygen uptake':ti,ab,kw OR vo2max:ti,ab,kw OR 'ergometer test*':ti,ab,kw OR 'six-minute walk*':ti,ab,kw OR '6-minute walk*':ti,ab,kw OR 'two-minute walk*':ti,ab,kw OR '2-minute walk*':ti,ab,kw OR 'muscle strength':ti,ab,kw OR 'muscular strength':ti,ab,kw OR 'muscle power':ti,ab,kw OR 'body strength':ti,ab,kw OR 'handgrip strength':ti,ab,kw OR 'hand strength':ti,ab,kw OR 'grip strength':ti,ab,kw OR dynamometer:ti,ab,kw OR 'one repetition maximum':ti,ab,kw OR '1rm':ti,ab,kw OR 'chair stand':ti,ab,kw OR 'arm curl':ti,ab,kw OR 'physical endurance':ti,ab,kw OR 'muscle endurance':ti,ab,kw OR 'push up':ti,ab,kw OR 'sit up':ti,ab,kw OR 'joint flexibility':ti,ab,kw OR 'shoulder flexibility':ti,ab,kw OR 'sit and reach':ti,ab,kw OR 'reach test*':ti,ab,kw OR 'back stretch*':ti,ab,kw OR '8-foot up-and-go':ti,ab,kw OR 'balance test*':ti,ab,kw OR 'physical function*':ti,ab,kw OR gait:ti,ab,kw OR 'walking speed*':ti,ab,kw	
	6	#4 OR #5	
Web of Science		TS=((cancer* OR carcinoma* OR neoplasm* OR adenoma* OR adenocarcinom* OR tumour* OR tumor* OR malignan*) NEAR/3 breast) AND TS=("physical fitness" OR "cardiorespiratory fitness" OR "treadmill test*" OR "step test*" OR "maximal oxygen uptake" OR VO2max OR "ergometer test*" OR "six-minute walk*" OR "6-minute walk*" OR "two-minute walk*" OR "2-minute walk*" OR "muscle strength" OR "muscular strength" OR "muscle power" OR "body strength" OR "handgrip strength" OR "hand strength" OR "grip strength" OR dynamometer OR "one repetition maximum" OR "1RM" OR "chair stand" OR "arm curl" OR "physical endurance" OR "muscle endurance" OR "push up" OR "sit up" OR "joint flexibility" OR "shoulder flexibility" OR "sit and reach" OR "reach test*" OR "back stretch*" OR "sargent jump" OR "8-foot up-and-go" OR "balance test*" OR "physical function*" OR gait OR "walking speed*") AND TS=(intervention* OR trial* OR experiment* OR program* OR rehabilitation OR physiotherapy OR "physical activity program" OR "exercise intervention" OR "training program" OR "resistance training" OR "aerobic exercise" OR yoga OR Pilates OR "Tai Chi" OR Qigong OR "home-based exercise" OR "supervised exercise" OR "combined exercise" OR gardening OR "lifestyle intervention" OR "lifestyle modification" OR "diet intervention" OR "nutritional support" OR "behavioral counseling")	2,497
KoreaMed	1	유방암 OR 유방암 생존자 OR breast cancer	29
	2	체력 OR 심폐체력 OR 근력 OR 근지구력 OR 악력 OR 유연성 OR 신체기능 OR 보행속도 OR balance OR physical fitness OR cardiorespiratory fitness OR muscle strength OR grip strength OR flexibility OR physical function OR gait OR walking speed	
	3	생존자 OR 암생존자 OR survivorship OR survivor	
	4	운동 OR 운동중재 OR 운동프로그램 OR 재활 OR 신체활동 OR intervention OR rehabilitation OR exercise OR trial	
	5	1 AND 2 AND 3 AND 4	
KCI	1	유방암 OR 유방암 생존자 OR breast cancer OR breast tumor	52
	2	체력 OR 심폐체력 OR 근력 OR 근지구력 OR 악력 OR 유연성 OR 신체기능 OR 보행속도 OR balance OR physical fitness OR cardiorespiratory fitness OR muscle strength OR grip strength OR flexibility OR physical function OR gait OR walking speed	
	3	생존자 OR 암생존자 OR survivorship OR survivor	
	4	운동 OR 운동중재 OR 운동프로그램 OR 재활 OR 신체활동 OR intervention OR rehabilitation OR exercise OR trial	
	5	1 AND 2 AND 3 AND 4	

The asterisk* is a wildcard character used to capture all possible word endings (e.g., “cancer*” retrieves “cancer” and “cancers”).

Table S4. Studies Categorized by Phase: Pretreatment Phase (N = 5).

No.	Author (year)	Sample size	Participant Characteristics	Type of fitness outcome	Intervention duration	Intervention type	Key Results
1	Yuan R., et al. (2024)	N = 79	Mean Age (years):<br>- Mirror group: 52.68 ± 10.97<br>- Control group: 54.43 ± 11.9<br>Mean BMI (kg/m²):<br>- Mirror group: 22.17 ± 2.57<br>- Control group: 22.96 ± 1.83	Muscular Strength, Flexibility	8 weeks	Exercise intervention (Home based → Stretching)	8-week mirror therapy-based ROM exercise – Shoulder flexion, abduction, strength – Flexibility ↑, Strength no improvement
2	Charati F. G. et al (2022)	N = 76	Mean Age (years):<br>- Intervention group: 38.14 ± 10.70<br>- Control group: 42.63 ± 8.11	Cardiorespiratory fitness	5 weeks	Exercise intervention (Home based → Combined Exercise)	After 5 weeks stretching + aerobic exercise → Physical function ↑ (6-minute walk test [6MWT]) in breast cancer patients
3	de Souza, A. P. S., et al. (2021)	N = 34	Mean Age (years): 44.3 ± 9.2<br>Mean BMI (kg/m²):<br>- Intervention group: 26.9 ± 2.5<br>- Control group: 27.8 ± 6.1	Muscular Strength	8.4 weeks	Non-exercise intervention (Nutrition)	Control group → grip strength ↓ (p = 0.009) intervention group → no significant change (p = 0.125)
4	Rafn B. S., et al. (2018)	N = 41	Mean Age (years): 54.2 ± 8.3<br>Mean BMI (kg/m²): 27.2 ± 6.8	Muscular Strength, Flexibility	48 weeks	Non-exercise intervention (Targeted physiotherapy)	Prospective surveillance and targeted physiotherapy (PSTP) for breast cancer survivors (BCS) → Grip strength ↑ (vs. education), Shoulder external rotation strength: No change
5	Lee, J. H., et al. (2006)	N = 60	Mean Age (years): Not reported (most were in their 40s)	Muscular Strength	16 weeks	Exercise intervention (Home based → Combined Exercise)	Exercise program for breast cancer survivors (BCS) → Muscle flexibility ↑, Upper body endurance ↑, Shoulder function ↑

↑ indicates increased or improved; ↓ indicates decreased or deteriorated.

Table S5. Studies Categorized by Phase: During treatment Phase (N = 101).

No.	Author (year)	Sample size	Participant Characteristics	Type of fitness outcome	Intervention duration	Intervention type	Key Results
1	Brahmbhatt et al. (2024)	N = 72	Mean Age (years):<br>- Intervention group: 57.4 ± 11.94<br>- Control group: 54.0 ± 10.69<br><br>Mean BMI (kg/m²):<br>- Intervention group: 27.4<br>- Control group: 28.2	Cardiorespiratory fitness, Muscular Strength	Not reported	Exercise intervention (Home based → Combined Exercise)	Intervention group → fitness performance ↑ (6-minute walk test [6MWT], grip strength)
2	Huo M., et al. (2024)	N = 162	Mean Age (years): 50.5 ± 11.5<br>Mean BMI (kg/m²): 24.7 ± 3.7	Muscular Strength, Flexibility	0.57 weeks	Exercise intervention (Supervised → Stretching, Resistance Exercise)	Grip strength showed a statistically significant improvement within each group before and after the intervention (within-group pre-post), but no significant difference was observed between the groups.
3	Koevoets, E. W., et al. (2024)	N = 181	Mean Age (years):<br>- Exercise group: 51.7 ± 9.2<br>- Control group: 53.1 ± 8.9	Cardiorespiratory fitness	24 weeks	Exercise intervention (Supervised → Combined Exercise)	Exercise after chemotherapy -→ improve peak oxygen uptake (VO_2 peak) ↑ and High VO2peak ↔ CBF in whole brain
4	Morano, T. et al. (2024)	N = 160	Mean Age (years): 52.85 ± 7.26 (Total group)	Muscular Strength, Flexibility	12 weeks	Exercise intervention (Supervised → Combined Exercise, Stretching)	12-week exercise program → flexibility ↑, strength ↑, muscle quality index ↑ Physical fitness influenced by age, radiation therapy, and chemotherapy
5	Antunes P., et al. (2024)	N = 93	Mean Age (years): 50.33 ± 9.46<br>Mean BMI (kg/m²): 27.82 ± 5.73	Muscular Strength	20 weeks	Exercise intervention (Supervised → Combined Exercise)	Supervised Exercise Training Program (SETP) → mitigated decline in functional outcomes: handgrip strength and 30-second chair sit-to-stand
6	Borsati A. et al. (2024)	N = 47	Mean Age (years): 54.24 ± 9.21<br>Mean BMI (kg/m²): 27.3 ± 6.20	Cardiorespiratory fitness, Muscular Strength	12 weeks	Exercise intervention (Supervised, Home based → Combined Exercise)	12-week exercise program → cardiorespiratory fitness ↑ (6-minute walk test [6MWT]) handgrip & leg press strength: no significant change
7	Skouras A. Z., et al. (2024)	N = 19	Mean Age (years): 49.08<br>Mean BMI (kg/m²): 23.40	Cardiorespiratory fitness, Muscular Strength	12 weeks	Exercise intervention (Supervised → Combined Exercise)	Maintaining stable ACWR (Acute: Chronic Workload Ratio) 0.8–1.3 (from week 6) → Physical & health outcomes ↑ (Strength: no significant change)
7	Reis, A.D. et al. (2023)	N = 31	Mean Age (years):<br>- TG group: 46.9 ± 7.4<br>- CG group: 51.8 ± 12.5	Cardiorespiratory fitness, Muscular Strength	12 weeks	Exercise intervention (Supervised → Combined Exercise)	12-week combined training (CT) program in breast cancer survivors (BCS) → VO_2 max ↑, Handgrip strength ↑
8	Hiraoui, M, et al.(2023)	N = 39	Mean Age (years):<br>- Training group: 49.71 ± 5.41<br>- Control group: 48.93 ± 4.76	Muscular Strength	6 weeks	Exercise intervention (Combined → Combined Exercise)	6 weeks supervised muscle strength and aerobic training programs=muscle activity↑, muscular performance↑

			Mean BMI (kg/m²):<br>- Training group: 28.42 ± 2.55<br>- Control group: 27.55 ± 2.62				
10	Leung, A. K., et al. (2023)	N = 108	Mean Age (years):<br>- Experimental group: 57.4 ± 11.7<br>- Control group: 61.8 ± 12.7	Muscular Strength, Flexibility	6 weeks	Exercise intervention (Combined → Combined Exercise)	There was no significant difference in the affected hang grip strength groups ($p > 0.05$)
10	Moulton C., et al. (2023)	N = 20	Mean Age (years): 50.55 ± 5.69<br>Mean BMI (kg/m²):<br>- EG group: 23.10 ± 2.53<br>- CG group: 21.38 ± 0.50	Cardiorespiratory fitness, Muscular Strength, Flexibility	16 weeks	Exercise intervention (Home based → Combined Exercise)	16-week online exercise program significantly improved Physical functions in breast cancer patients, including 6-minute walk (6MW) capacity ↑, flexibility ↑, and fatigue ↓
12	Bertoli, J., et al. (2023)	N = 43	Mean Age (years):<br>- Pilates group: 55.2 ± 7.7<br>- Control group: 54.7 ± 6.7<br>Mean BMI (kg/m²):<br>- Pilates group: 29.1 ± 4.7<br>- Control group: 30.3 ± 4.7	Muscular Strength, Flexibility	12 weeks	Exercise intervention (Supervised → Stretching, Resistance Exercise)	Pilates exercise during hormone therapy → overall muscle strength ↑
13	Shi B., et al. (2023)	N=108	Mean Age (years): 50.32 ± 9.7<br>Mean BMI (kg/m²): 23.48 ± 2.75	Muscular Strength, Flexibility	18 weeks	Exercise intervention (Home based → Combined Exercise)	KAP (Knowledge–attitude–practice) theory-based lymphedema prevention program→ Arm function strength ↑
13	Antunes, P., et al. (2023)	N = 93	Mean Age (years): 50.33 ± 9.46<br>Mean BMI (kg/m²): 27.82 ± 5.73	Cardiorespiratory fitness	20 weeks	Exercise intervention (Supervised → Combined Exercise)	Exercise intervention (during chemotherapy aerobic + resistance) → Estimated peak VO_2 ↑ (end of chemotherapy & 3 months post-treatment)
15	Foulkes, S. J., et al. (2023)	N = 104	Mean Age (years):<br>- ExT group: 50.3 ± 7.7<br>- UC group: 51.2 ± 7.6<br>Mean BMI (kg/m²):<br>- ExT group: 27.5 ± 4.6<br>- UC group: 27.5 ± 5.6	Cardiorespiratory fitness	48 weeks	Exercise intervention (Combined → Combined Exercise)	12-month exercise training (ExT) for breast cancer survivors (BCS) → VO_2 peak ↑, % predicted VO_2 peak ↑, Oxygen pulse ↑
16	Scott, J. M., et al. (2023)	N = 158	Mean Age (years): 47 ± 11<br>Mean BMI (kg/m²): 27 ± 6	Cardiorespiratory fitness	32 weeks	Exercise intervention (Supervised → Steady-state Continuous Exercise)	Exercise therapy during or after chemotherapy for breast cancer survivors (BCS) → Cardiorespiratory fitness: No change (compared to usual care)
17	Qureshi A., et al. (2023)	N = 120	Mean Age (years): Not reported<br>Mean BMI (kg/m²):<br>- T_0: 25.26 ± 0.35<br>- T_1: 26.84 ± 0.28<br>- T_2: 26.48 ± 0.28	Muscular Strength	12 weeks	Non-exercise intervention (Nutrition)	Natural supplement (T_2) for breast cancer survivors (BCS) → Hand grip strength: No change

18	Isanejad, A., et al. (2023)	N = 30	Mean Age (years): 45.13 ± 6.86<br>Mean BMI (kg/m²): 27.51 ± 4.96	Cardiorespiratory fitness, Muscular Strength	12 weeks	Exercise intervention (Supervised → High-Intensity Interval Training, Steady-state Continuous Exercise)	12-week high-intensity interval training (HIIT) for breast cancer survivors (BCS) → VO_2 peak ↑ (no group difference)
19	Pirincci C. S., et al. (2023)	N = 32	Mean Age (years):<br>- CDP+SSE group: 54.38 ± 9.09<br>- CDP group: 55.25 ± 8.17<br>Mean BMI (kg/m²):<br>- CDP+SSE group: 27.04 ± 2.32<br>- CDP group: 26.63 ± 2.81	Muscular Strength	8 weeks	Exercise intervention (Supervised → Combined Exercise)	Combined decongestant Physical therapy (CDP)+Scapulothoracic stabilization exercise (SSE) in breast cancer patients with lymphedema after mastectomy > CDP → ↑ scapulothoracic strength, scapular endurance
19	Hiraouih M. et al. (2022)	N = 32	Mean Age (years):<br>- Training group: 49.71 ± 5.41<br>- Control group: 48.93 ± 4.76<br>Mean BMI (kg/m²):<br>- Training group: 28.42 ± 2.55<br>- Control group: 27.55 ± 2.62	Muscular Strength	6 weeks	Exercise intervention (Combined → Combined Exercise)	6-week training program → muscle oxygen utilization ↑, strength ↑, endurance ↑
20	Bjørke, A. C. H., et al. (2022)	N = 255	Mean Age (years):<br>- LMI group: 53 ± 10<br>- HI group: 55 ± 11	Cardiorespiratory fitness	24 weeks	Exercise intervention (Combined → Resistance Exercise)	Age and endurance training compliance significantly modulated the intervention effects of high-intensity (HI) exercise and low-to-moderate intensity (LMI) exercise on cardiorespiratory fitness (CRF), respectively.
21	Sturgeon, K. M., et al. (2022)	N = 19	Mean Age (years): 49.4 ± 10.5	Cardiorespiratory fitness	24 weeks	Exercise intervention (Home based → Combined Exercise)	24-week remote exercise (for BCP [Breast Cancer Patients] starting chemotherapy) →VO_2 max ↓ −1.2% (intervention) vs. ↓ −24.5% (control) $p = 0.04$
23	Feyzioğlu, Ö. Z. L. E. M., et al. (2022)	N = 15	Mean Age (years): 50.13 ± 8.79<br>Mean BMI (kg/m²): 29.6 ± 4.41	Muscular Strength, Flexibility	Not reported	Exercise intervention (Supervised → etc.)	Video game-based exercise for breast cancer survivors (BCS) → Shoulder muscle strength: No change
24	Myers S. A., et al. (2022)	N = 27	Mean Age (years): 55.5 ± 10.2	Muscular Strength, Physical function	6 weeks	Exercise intervention (Supervised → Combined Exercise)	6-week supervised virtual strength and aerobic exercise for breast cancer survivors (BCS) → Lower body strength ↑ (chair stand) Balance: No change
25	Muñoz-Fernández, M. J., et al. (2021)	N = 40	Mean Age (years):<br>- Experiment group: 59.25 ± 7.52<br>- Control group: 64.15 ± 11.78<br>Mean BMI (kg/m²):<br>- Experiment group: 25.73 ± 4.31<br>- Control group: 26.76 ± 3.17	Muscular Strength, Flexibility	4 weeks	Exercise intervention (Combined → Stretching)	The early Physical therapy intervention positively impacted the maintenance or improvement of grip strength.

26	Bøhn S, K. et al. (2021)	N = 55	Mean Age (years):<br>- Intervention group: 55.7 ± 7.8<br>- Control group: 54.3 ± 7.7<br>Mean BMI (kg/m²):<br>- Intervention group: 24.8 ± 3.1<br>- Control group: 25.0 ± 3.6	Cardiorespiratory fitness	48 weeks	Exercise intervention (Combined → Combined Exercise)	The exercise program in the intervention group effectively prevented the decline in maximal oxygen uptake (VO_2 max) (p = 0.005).
27	Vander Walde, N. A., et al. (2021)	N = 55	Median Age (years):<br>- HBGWP group: 69 (Range: 66–84)<br>- Fixed Rec. group: 68 (Range: 65–83)	Physical function	6 weeks	Exercise intervention (Home based → Steady-state Continuous Exercise)	Short Physical Performance Battery (SPPB) → no association with increased walking (↔)
27	de Jesus Leite, M. A. F., et al. (2021)	N = 14	Mean Age (years):<br>- Postmenopausal BCS group: 63.6 ± 7.2<br>- Healthy postmenopausal women group: 60.4 ± 7.4<br>Mean BMI (kg/m²):<br>- Postmenopausal BCS group: 29.2 ± 5.3<br>- Healthy postmenopausal women group: 29.7 ± 4.9	Muscular Strength	12 weeks	Exercise intervention (Supervised → Resistance Exercise)	12-week resistance exercise intervention → Muscle strength ↑ (all exercises) ↓ to pre-RT level after cessation
28	de Sire, A., et al. (2021)	N = 22	Mean Age (years): 51.73 ± 10.73<br>Mean BMI (kg/m²):<br>- Group A: 25.56 ± 5.17<br>- Group B: 27.31 ± 3.84	Cardiorespiratory fitness, Muscular Strength	4 weeks	Exercise intervention (Supervised → Resistance Exercise)	4-week exercise with whole-body vibration (WBV) in breast cancer survivors (BCS) with AIMSS → Physical fitness ↑ (no group difference)
30	Lee, K., et al. (2021)	N = 30	Mean Age (years): 46.9 ± 9.8<br>Mean BMI (kg/m²): 31.0 ± 7.5	Cardiorespiratory fitness, Muscular Strength, Physical function	8 weeks	Exercise intervention (Supervised → High-Intensity Interval Training)	High-intensity interval training (HIIT) for breast cancer survivors (BCS) → Lower limb power ↑, Cardiopulmonary endurance
31	An, K. Y et al. (2020)	N = 301	Mean Age (years): 50.0 ± 8.7<br>Mean BMI (kg/m²): 26.3 ± 5.5	Cardiorespiratory fitness, Muscular Strength	12~18 weeks	Exercise intervention (Supervised → Steady-state Continuous Exercise, Combined Exercise)	Standard Aerobic Exercise (STAN) → VO_2 peak ↑, Strength ↑, Compliance ↑, Difficulty ↓<br>High-Dose Aerobic Exercise (HIGH) → VO_2 peak ↑↑, Strength ↑, Compliance ↑, Difficulty ↓<br>Combined Aerobic + Resistance Exercise (COMB) → VO_2 peak ↑, Strength ↑↑, Compliance ↑↑, Difficulty ↓
31	Vincent, F et al. (2020)	N = 94	Mean Age (years): 56.5 (30–69)<br>Mean BMI (kg/m²):<br>- Group A: 25.2 ± 5.2	Cardiorespiratory fitness, Muscular Strength	48 weeks	Exercise intervention (Home based → Combined Exercise)	There was improvement in peak oxygen uptake (VO_2 peak) and 6-minute walk (6MW) distance in all groups after 12 months, but there was no significant difference.

			- Group B: 25.5 ± 4.5<br>- Group C: 25.9 ± 4.8				
32	Delrie L. et al. (2020)	N = 49	Mean Age (years): 55.00 ± 10.00<br>Mean BMI (kg/m²): 26.10 ± 5.80	Cardiorespiratory fitness, Muscular Strength	24 weeks	Exercise intervention (Home based → Steady-state Continuous Exercise)	ABLE (Advanced stage Breast cancer and Lifestyle Exercise) Trial → Flexible Physical activity intervention for patients with metastatic breast cancer helps maintain Physical function.
33	Feyzioğlu, Ö., et al. (2020)	N = 40	Mean Age (years):<br>- KBRG group: 50.84 ± 8.53<br>- SPTG group: 51.00 ± 7.06<br>Mean BMI (kg/m²):<br>- KBRG group: 30.06 ± 4.73<br>- SPTG group: 28.97 ± 6.14	Muscular Strength, Flexibility	6 weeks	Exercise intervention (Home based → etc.)	Kinect-based VR therapy for breast cancer survivors (BCS) → Upper limb strength ↑, Grip strength ↑<br>→ KBRG ↑, SPTG ↑, Between-group difference: None
35	Song M. J., et al. (2020)	N = 12	Mean Age (years): 53 ± 5.1<br>Mean BMI (kg/m²): 25.6 ± 2.8	Cardiorespiratory fitness, Muscular Strength	6 weeks	Exercise intervention (Supervised → Combined Exercise)	6-week aerobic and resistance exercise for breast cancer survivors (BCS) after mastectomy → Hand grip strength ↑, 6-minute walk test (6MWT) ↑
35	Moller T., et al. (2020)	N = 153	Mean Age (years): 51.7 ± 9.4<br>Mean BMI (kg/m²): 26.1 ± 5.1	Cardiorespiratory fitness, Muscular Strength	12 weeks	Exercise intervention (Supervised, Home based → Combined Exercise, High-Intensity Interval Training, Steady-state Continuous Exercise)	Supervised hospital-based vs. pedometer exercise in Physically inactive breast cancer survivors (BCS) → VO_2 peak ↑ (both), Muscle strength ↑ (supervised only)
36	Tomić, S., et al. (2020)	N=149	Mean Age (years): 58.0 ± 13.0	Flexibility	12.6 weeks	Exercise intervention (Combined→ Stretching)	combined 12 weeks ROM exercise after surgery → Shoulder ROM ↑
38	da Silveira, D. S. P., et al. (2020)	N=32	Mean Age (years): 52.2 ± 8.3<br>Mean BMI (kg/m²): 27.6 ± 4.7	Muscular Strength, Flexibility	4 weeks	Non-exercise intervention (Standard rehabilitation + neuromuscular facilitation)	4-week standard rehabilitation+PNF intervention after surgery → Ugrip strength and shoulder ROM ↑
39	Alizadeh, A. M., et al. (2019)	N = 50	Mean Age (years):<br>- Usual care group: 48.42 ± 7.54<br>- HIT group: 49.2 ± 9.7	Cardiorespiratory fitness	12 weeks	Exercise intervention (Supervised → High-Intensity Interval Training)	12-week high-intensity interval training (HIIT) → maximal oxygen uptake (VO_2 max) ↑, inflammatory cytokines ↓

40	Ariza-Garcia A., et al. (2019)	N = 68	Mean Age (years):<br>- e-CuidateChemo group: 48.82 ± 7.68<br>- Control group: 47.32 ± 9.92	Cardiorespiratory fitness, Muscular Strength	8 weeks	Exercise intervention (Supervised → Combined Exercise)	e-CuidateChemo (8 weeks) → 6-minute walk test (6MWT) ↑, core & lower-body strength ↑ body composition & handgrip ↔
41	Bloomquist, K.,et al. (2019)	N = 153	Mean Age (years): 51.7 ± 9.4<br>Mean BMI (kg/m²): 26.1 ± 5.1	Muscular Strength	12 weeks	Exercise intervention (Supervised, Home based → Combined Exercise, Steady-state Continuous Exercise)	High-load resistance exercise during chemotherapy-→ upper body muscular syrength↑
41	Bolam K. A. et al. (2019)	N=206	Mean Age (years):<br>- RT-HIIT group: 52.7 ± 10.3<br>- AT-HIIT group: 54.4 ± 10.3<br>- Usual care group: 52.6 ± 10.2<br>Mean BMI (kg/m²):<br>- RT-HIIT group: 25.1 ± 4.3<br>- AT-HIIT group: 24.8 ± 4.4<br>- Usual care group: 24.6 ± 4.8	Cardiorespiratory fitness, Muscular Strength	16 weeks	Exercise intervention (Supervised → High-Intensity Interval Training)	Only RT-HIIT → lower limb muscle strength ↑<br>RT-HIIT & AT-HIIT → No significant differences in hand grip strength or cardiorespiratory fitness between groups.
43	Cešeiko R. et al. (2019)	N=55	Mean Age (years):<br>- TG group: 48.2 ± 6.7<br>- CG group: 49.0 ± 8.0	Muscular Strength	12 weeks	Exercise intervention (Supervised → Resistance Exercise)	12-week high-intensity strength training → muscle strength ↑ in breast cancer patients
44	Okumatsu, K., et al. (2019)	N = 32	Mean Age (years):<br>- Intervention group: 51 ± 6<br>- Usual care group: 53 ± 5<br>Mean BMI (kg/m²):<br>- Intervention group: 24.3 ± 4.2<br>- Usual care group: 21.9 ± 3.8	Cardiorespiratory fitness	12 weeks	Exercise intervention (Supervised → Combined Exercise)	After 12 weeks combined exercise + diet intervention → VO_2 peak ↑ (absolute & relative) vs. usual care ($p = 0.002$) Physical fitness improved alongside weight loss
45	de Paulo, T. R., et al. (2019)	N = 42	Mean Age (years): 51.73 ± 10.73<br>Mean BMI (kg/m²):<br>- Group A: 25.56 ± 5.17<br>- Group B: 27.31 ± 3.84	Cardiorespiratory fitness, Muscular Strength	24 weeks	Exercise intervention (Supervised → Combined Exercise)	24-week aerobic + resistance training (BCS vs. healthy postmenopausal women)<br>→ VO_2 peak ↑ (both groups)<br>→ Lower body strength ↑ (both groups)<br>→ Upper body strength ↑ (healthy women at 6 mo > BCS at 9 mo)
46	Santos, W. D. N. D., et al. (2019)	N = 26	Mean Age (years):<br>- Intervention group: 55.0 ± 5.8<br>- Control group: 54.3 ± 5.2<br>Mean BMI (kg/m²):<br>- Intervention group: 27.97 ± 5.01<br>- Control group: 26.78 ± 4.04	Muscular Strength	8 weeks	Exercise intervention (Supervised → Resistance Exercise)	8-week 1:1 supervised resistance exercise intervention → Leg press strength ↑ (34%) Bench press strength ↑ (20%)

46	Foulkes, S. J., et al. (2019)	N = 28	Mean Age (years):<br>- UC group: 58 ± 8<br>- ET group: 46 ± 9<br>Mean BMI (kg/m²):<br>- UC group: 28.1 ± 5.5<br>- ET group: 24.0 ± 3.3	Cardiorespiratory fitness	12 weeks	Exercise intervention (Combined → Combined Exercise)	Exercise during AC chemotherapy for breast cancer survivors (BCS) → VO_2 peak: No change, Cardiac function: No change
47	Hiraoui, M., et al. (2019)	N = 32	Mean Age (years):<br>- Training group: 49.71 ± 5.41<br>- Control group: 48.93 ± 4.76<br>Mean BMI (kg/m²):<br>- Training group: 28.42 ± 2.55<br>- Control group: 27.55 ± 2.62	Cardiorespiratory fitness	6 weeks	Exercise intervention (Combined → Combined Exercise)	Training intervention for breast cancer survivors (BCS) → Walking speed ↑, Walking distance ↑ (compared to control group)
49	Howden, E.J., et al. (2019)	N = 28	Mean Age (years):<br>- Usual care group: 52 ± 12<br>- Exercise training group: 42 ± 9<br>Mean BMI (kg/m²):<br>- Usual care group: 27.8 ± 6.2<br>- Exercise training group: 25.2 ± 7.6	Cardiorespiratory fitness	12 weeks	Exercise intervention (Combined → Steady-state Continuous Exercise)	12-week exercise during anthracycline chemotherapy for breast cancer survivors (BCS) → VO_2 peak ↑ (p group*time = .003), arterio-venous O_2 difference ↓ (p = .035) vs. control
50	Kirkham, A. A., et al. (2019)	N = 73	Mean Age (years): 50.8 ± 10.6	Cardiorespiratory fitness, Muscular Strength	10 weeks	Exercise intervention (Combined → Combined Exercise)	10-week exercise program for breast cancer survivors (BCS) → Maximal oxygen uptake (VO_2 peak) ↑, Heart rate recovery ↑, Upper and lower body strength (1RM) ↑
50	Lee, K., et al. (2019)	N = 30	Mean Age (years): 46.9 ± 9.8<br>Mean BMI (kg/m²): 31.6 ± 7.7	Cardiorespiratory fitness	8 weeks	Exercise intervention (Supervised → High-Intensity Interval Training)	High-intensity interval training (HIIT) for breast cancer survivors (BCS) → VO_2 max, Peak power output (PPO): No change
52	Mijwel S., et al. (2019)	N = 206	Mean Age (years):<br>- RT-HIIT group: 52.7 ± 10.3<br>- AT-HIIT group: 54.4 ± 10.3<br>- UC group: 52.6 ± 10.2	Cardiorespiratory fitness, Muscular Strength	16 weeks	Exercise intervention (Supervised → High-Intensity Interval Training)	Resistance training + high-intensity interval training (RT-HIIT) and aerobic training + high-intensity interval training (AT-HIIT) during chemotherapy for breast cancer survivors (BCS) → VO_2 peak ↑, Lower limb strength ↑, Grip strength ↑
52	Schulz et al. (2018)	N = 26	Mean Age (years):<br>- Intervention group: 51.9 ± 9.8<br>- Control group: 56.9 ± 7.0	Cardiorespiratory fitness, Muscular Strength	6 weeks	Exercise intervention (Supervised → Combined Exercise)	The intervention group → endurance (peak oxygen uptake [VO_2 peak] increased by 12.0 ± 13.0%) and strength performance (cumulative load increased by 25.9 ± 11.2%) ↑
53	Baker, M. K., et al. (2018)	N = 31	Mean Age (years): 61.6 ± 8.3<br>Mean BMI (kg/m²): 28.8 ± 4.7	Cardiorespiratory fitness, Muscular Strength, Physical function	12 weeks	Exercise intervention (Not reported→ etc.)	Whole-Body Vibration Training → Physical function & body composition: no significant change (↔)
55	Conejo, I., et. al. (2018)	N = 40	Median Age (years): 66.30 (Range: 50–82)	Muscular Strength	5 weeks	Exercise intervention (Not reported→ etc.)	Tai Chi → Grip strength ↑ (not significant p > 0.05)

			Median BMI (kg/m²): 28.17 (Range: 16.17–47.79)				
56	Lim, K. H. (2018).	N = 48	Mean Age (years): Unreported (mostly 40s–50s)	Muscular Strength, Flexibility	7 weeks	Exercise intervention (Supervised → Resistance Exercise)	7-week customized upper extremity exercise for breast cancer survivors (BCS) → Grip strength ↑ (exercise group), Control: No change
57	Scott, J. M., et al. (2018)	N = 65	Mean Age (years): 54 ± 11<br>Mean BMI (kg/m²): 28 ± 16	Cardiorespiratory fitness, Muscular Strength, Physical function	12 weeks	Exercise intervention (Supervised → Steady-state Continuous Exercise)	12-week aerobic exercise for metastatic breast cancer (MBC) → VO_2 peak ↑, Functional capacity ↑ (only in patients with high adherence not in full sample)
58	Kneis, S., et al. (2018)	N = 21	Mean Age (years):<br>- IG group: 52 (39–72)<br>- CG group: 64 (48–79)<br>Mean BMI (kg/m²):<br>- IG group: 24.0 (20.9–28.8)<br>- CG group: 26.7 (20.2–35.5)	Cardiorespiratory fitness, Muscular Strength, Flexibility, Physical function	6 weeks	Exercise intervention (Supervised → Combined Exercise)	6-week exercise intervention for breast cancer survivors (BCS) → Lower body muscle power ↑ , Maximal power output ↑ (vs. control)
58	Mijwel, S. et al. (2018)	N=206	Mean Age (years): 53.2 ± 10.3	Cardiorespiratory fitness, Muscular Strength	16 weeks	Exercise intervention (Supervised→ Combined Exercise)	16-week resistance or aerobic with HIIT during chemotherapy → VO2peak: RT-HIIT&AT-HIIT- vs. control, lower-limb strength, grip strength ↑ in RT-HIIT
60	Marize Ibrahim, MSc. PT et al. (2017)	N = 59	Mean Age (years): 51.2 ± 7.9<br>Mean BMI (kg/m²): 24.57 ± 2.60	Muscular Strength, Flexibility	12 weeks	Exercise intervention (Combined → Combined Exercise)	These results did not sustain over time, and by 6 months post-radiation (T4), there were no statistically significant differences between the two study groups.
60	Ammitzbøll G. et al. (2017)	N = 8	Mean Age (years): 57.4 ± 7.5<br>Mean BMI (kg/m²): 24 ± 2.8	Muscular Strength, Flexibility	20 weeks	Exercise intervention (Supervised → Resistance Exercise)	20-week progressive strength training → muscle strength ↑ some decline by 50 weeks, but strength remained above baseline → highlights need for continuous intervention
62	Khan, Q. J., et al.(2017)	N = 160	Mean Age (years):<br>- Placebo group: 62 (54–69)<br>- High-dose $VitD_3$ group: 60.5 (55–71)<br>Mean BMI (kg/m²):<br>- Placebo group: 29.6 (26.8–33.6)<br>- High-dose $VitD_3$ group: 29.9 (25.6–34.4)	Muscular Strength	24 weeks	Non-exercise (Vitamin-D supplementation)	High-dose vitamin D supplementation for breast cancer survivors (BCS) → Hand grip strength: No change (vs. placebo)
63	Mostarda C., et al. (2017)	N = 27	Age Range (years): 30–59	Cardiorespiratory fitness	4 weeks	Exercise intervention (Supervised → Combined Exercise)	1-month combined exercise training for breast cancer survivors (BCS) receiving adjuvant therapy → Maximal oxygen uptake (VO_2 max) ↑

64	Wiskemann, J., et al. (2017)	N=146	Mean Age (years): 55.2 ± 9.2<br>Mean BMI (kg/m²): 27.0 ± 5.1	Muscular Strength, Flexibility	12 weeks	Exercise intervention (Supervised → Resistance Exercise)	12-week resistance training in radiotherapy → Upper body and lower body strength, ↑
65	Arem H., et al. (2016)	N = 121	Mean Age (years): 62.0 ± 7.0<br>Mean BMI (kg/m²): 30.0 ± 6.8	Cardiorespiratory fitness	48 weeks	Exercise intervention (Supervised → Combined Exercise)	Supervised aerobic + resistance training during hormone therapy → VO_2 max ↑ 5.1%
66	Cornette, T., et al. (2015)	N = 44	Median Age (years):<br>- APA group: 52 (Range: 37–73)<br>- Control group: 49 (Range: 37–68)<br><br>Mean BMI (kg/m²):<br>- APA group: 23.8 ± 3.8<br>- Control group: 26.6 ± 3.7	Cardiorespiratory fitness, Muscular Strength	27 weeks	Exercise intervention (Home based → Combined Exercise)	27-week home-based APA (Adapted Physical Activity) during chemo-/radiotherapy→ VO_2 peak (Peak Oxygen Uptake) ↑, 6MWT (6-Minute Walk Test) ↑, Pmax (Peak Power Output) ↑, Quadriceps strength ↔
67	Travier NE., et al. (2015)	N = 204	Mean Age (years):<br>- Intervention group: 49.7 ± 8.2<br>- Usual care group: 49.5 ± 7.9<br>Mean BMI (kg/m²):<br>- Intervention group: 25.8 ± 4.4<br>- Usual care group: 26.6 ± 5.2	Cardiorespiratory fitness, Muscular Strength	18 weeks	Exercise intervention (Supervised → Combined Exercise)	18-week supervised exercise PACT (Physical Activity during Cancer Treatment)<br>→ Short-term: Physical fatigue ↓, Submaximal cardiorespiratory fitness decline attenuated, Muscle strength ↑ (all significant)<br>→ No significant effects at 36week follow-up
68	Schmidt T., et al. (2015)	N = 67	Mean Age (years):<br>- RT group: 53 ± 12.55<br>- ET group: 56 ± 10.15<br>- SC group: 54 ± 11.19	Cardiorespiratory fitness, Muscular Strength	12 weeks	Exercise intervention (Supervised → Resistance Exercise, Combined Exercise)	12-week exercise training during chemotherapy for breast cancer patients → Muscular strength ↑ (resistance and endurance groups), Endurance ↓ (all groups least in resistance group)
69	Moller T., et al. (2015)	N = 45	Mean Age (years): 50.83 ± 10.29<br>Mean BMI (kg/m²): 24.61 ± 4.42	Cardiorespiratory fitness	12 weeks	Exercise intervention (Supervised, Home based → Combined Exercise, Steady-state Continuous Exercise)	Taxane-based chemotherapy with Neulasta in Physically inactive breast cancer survivors (BCS) → VO_2 peak ↓ in all groups (hospital-based supervised exercise, home-based pedometer, control)
69	Benton, M. J. et al. (2014)	N = 20	Mean Age (years):<br>- YRT group: 51.7 ± 1.7<br>- ORT group: 68.3 ± 2.4<br>Mean BMI (kg/m²):<br>- YRT group: 28.2 ± 1.7<br>- ORT group: 27.4 ± 1.0	Muscular Strength, Flexibility	8 weeks	Exercise intervention (Supervised → Resistance Exercise)	After 8 weeks of resistance training → young resistance training (YRT, 40–59 years) and old resistance training (ORT, 60–80 years) groups: muscle strength and functional fitness ↑
71	Husebø, A.M., et al. (2014)	N = 67	Mean Age (years): 52.2 ± 9.3	Cardiorespiratory fitness	17 weeks	Exercise intervention (Home based → Combined Exercise)	6-month post-chemotherapy in breast cancer survivors (BCS) → 6-minute walk test (6MWT) ↑ (compared to baseline), No change between groups

71	Reis, D., et al. (2013)	N = 41	Mean Age (years): 55 ± 11<br>Mean BMI (kg/m²): 27.7 ± 6.3	Cardiorespiratory fitness	12 weeks	Exercise intervention (Home based → etc.)	Aerobic capacity improved in both the intervention and control groups, with no statistically significant differences found between the Nia group and the control group.
72	Vincent F., et al. (2013)	N = 39	Mean Age (years): 49 ± 8.4<br>Mean BMI (kg/m²): 24 ± 7.4	Cardiorespiratory fitness	12 weeks	Exercise intervention (Home based → Steady-state Continuous Exercise)	Adherence rate of exercise program ↑ : Cardiopulmonary fitness↑ and Physical capacity↑ in breast cancer patients (p=0.001 for VO_2 peak, p=0.01 for 6MWT).
73	Courneya, K. S., et al. (2013)	N = 301	Mean Age (years): 50 ± 8.9<br>Mean BMI (kg/m²): 26.5 ± 5.5	Cardiorespiratory fitness, Muscular Strength	16.4 weeks	Exercise intervention (Supervised → Combined Exercise, Steady-state Continuous Exercise)	Combined aerobic and resistance exercise (COMB) group → upper and lower limb strength & endurance ↑↑↑ vs. Standard aerobic exercise (STAN) and High-volume aerobic exercise (HIGH) ($p < 0.05$)
75	Galantino, M. L., et. al. (2013)	N = 12	Median Age (years): 59 (Range: 49–76)	Flexibility, Physical function	8 weeks	Exercise intervention (Supervised → etc.)	8-week Tai Chi intervention for breast cancer survivors (BCS) → Flexibility (Sit and Reach Test) ↑
76	Scott. E., et al. (2013)	N = 90	Mean Age (years): 55.7 ± 9.5<br>Mean BMI (kg/m²):<br>- Intervention group: 29.6 ± 3.5<br>- Control group: 31.1 ± 5.6	Cardiorespiratory fitness	24 weeks	Exercise intervention (Supervised → Combined Exercise)	24-week lifestyle intervention for breast cancer survivors (BCS) → Predicted maximal oxygen uptake (VO_2 max) ↑
101	Singh, C., et al. (2013)	N=71	Mean Age (years): 43.5 ± 5.3<br>Mean BMI (kg/m²): 26.3 ± 1.2	Flexibility	0.03 weeks	Exercise intervention (Combined→ Combined Exercise)	Preoperative education after surgery → Shoulder ROM ↑
78	Galantino M. L., et al. (2012)	N = 10	Mean Age (years): 58 (50–71)	Flexibility, Physical function	8 weeks	Exercise intervention (Combined → etc.)	The yoga program → balance ↑ (functional reach: 24.36 cm → 39.19 cm, p = 0.048) and flexibility ↑ (sit and reach: 22.90 cm → 30.10 cm, p = 0.009), effective for fall risk reduction and functional support in cancer survivors
78	Nuri, R., et al. (2012)	N = 29	Mean Age (years): 58.27 ± 6.31<br>Mean BMI (kg/m²):<br>- Training group: 27.9 ± 3.5<br>- Control group: 27.4 ± 3.4	Cardiorespiratory fitness	15 weeks	Exercise intervention (Supervised → Combined Exercise)	15-week combination training → Physical fitness index ↑ in experimental group
80	Haines, T. P., et al. (2010)	N = 89	Mean Age (years):<br>- Intervention group: 55.9 ± 10.5<br>- Control group: 54.2 ± 11.5	Cardiorespiratory fitness, Muscular Strength, Flexibility, Physical function	24 weeks	Exercise intervention (Home based → Combined Exercise)	no significant difference between groups (intervention vs. control) in Physical capacity.
81	Dolan, L. B., et al. (2010)	N = 242	Mean Age (years): 49.2 (25–78)<br>Mean BMI (kg/m²): 26.6 ± 5.5	Cardiorespiratory fitness	17 weeks	Exercise intervention (Supervised → Steady-state	Exercise during chemotherapy→ Hemoglobin levels ↔ (no prevention of decline) VO_2 peak ↔ (aerobic capacity preserved)

						Continuous Exercise, Resistance Exercise)	
81	Ligibel, J. A., et al. (2010)	N = 41	Mean Age (years): 47 ± 7.3	Cardiorespiratory fitness	12 weeks	Exercise intervention (Home based → Steady-state Continuous Exercise)	12-week exercise intervention for breast cancer survivors (BCS) → Cardiopulmonary fitness ↑
83	Rogers L. Q., et al. (2009)	N=41	Mean Age (years): 53 ± 9	Cardiorespiratory fitness, Muscular Strength	12 weeks	Exercise intervention (Combined → Steady-state Continuous Exercise)	After 12 weeks moderate-intensity aerobic exercise intervention → Effective in maintaining and improving strength in cancer survivors
83	Courneya, K. S., et al. (2009)	N = 242	Mean Age (years): Unknown (<50: 114, ≥50: 87)	Cardiorespiratory fitness, Muscular Strength	17 weeks	Exercise intervention (Supervised → Steady-state Continuous Exercise, Resistance Exercise)	6-month follow-up→ Muscle strength ↑ → Exercise adherence ↑
85	Hwang, I. S., et al. (2009)	N = 40	Not reported	Muscular Strength, Flexibility	12 weeks	Exercise intervention (Not reported→ etc.)	12-week Tai Chi exercise for breast cancer survivors (BCS) → Shoulder flexibility ↑, Muscle strength: No change
85	Rogers L. Q., et al. (2009)	N = 41	Mean Age (years): 53 ± 9<br>Mean BMI (kg/m²):<br>- Intervention group: 30.9 ± 8.6<br>- Usual care group: 30.8 ± 8.6	Cardiorespiratory fitness, Muscular Strength	12 weeks	Exercise intervention (Combined → Combined Exercise)	Behavior change intervention based on social cognitive theory for breast cancer survivors (BCS) → Muscle strength ↑, Cardiopulmonary fitness ↑
87	Courneya, K. S., et al. (2008)	N = 242	Mean Age (years):<br>- Intervention group: 52.2 ± 10.9<br>- Control group: 56.6 ± 10.4<br>Mean BMI (kg/m²):<br>- Intervention group: 25.5 ± 3.6<br>- Control group: 25.9 ± 3.4	Cardiorespiratory fitness, Muscular Strength	17 weeks	Exercise intervention (Supervised → Steady-state Continuous Exercise, Resistance Exercise)	Exercise during chemotherapy (RET/AET vs. UC) → RET: Strength ↑ (nontaxane > taxane), AET: VO_2 peak ↑ (age < 50) ↔ (age ≥ 50)
87	Kilgour, R. D., et al. (2008)	N = 27	Mean Age (years):<br>- UC group: 49.1 ± 5.7<br>- HBC group: 50.6 ± 9.3	Muscular Strength, Flexibility	1.57 weeks	Exercise intervention (Home based → Stretching)	Home-based exercise (HBE) program for breast cancer survivors (BCS) → Shoulder strength: No change, Grip strength: No change
89	Todd, J., et al. (2008)	N=116	Mean Age (years): 56.9 ± 13.2<br>Mean BMI (kg/m²): 28.0 ± 5.7	Muscular Strength, Flexibility	0.2 weeks	Exercise intervention (Supervised→ Stretching)	early mobilization intervention after surgery → preventing decreasing shoulder ROM (early mobilisation group was −11.2° vs. delayed mobilisation (−2.9°), grip strength (-)
90	Courneya K. S., et al. (2007)	N = 242	Mean Age (years): 49.2 (25–78)<br>Mean BMI (kg/m²): 26.6 ± 5.5	Cardiorespiratory fitness, Muscular Strength	12 weeks	Exercise intervention (Supervised → Combined Exercise)	Aerobic exercise (AET) → maintains or improves peak oxygen uptake (VO_2 peak) during chemotherapy (p = 0.004, p = 0.006) Resistance exercise (RET) → increases muscular strength (one-repetition maximum [1RM]) (p = 0.001)

91	Battaglini, C., et al. (2007)	N = 20	Mean Age (years):<br>- Exercise group: 57.5 ± 23.0<br>- Control group: 56.6 ± 16.0	Cardiorespiratory fitness, Muscular Strength	21 weeks	Exercise intervention (Supervised → Combined Exercise)	Exercise group → overall muscle strength ↑ 9.57%
91	Beurskens C. H. et al. (2007)	N = 30	Mean Age (years):<br>- Intervention group: 53.7 ± 13.0<br>- Control group: 55.4 ± 9.3	Muscular Strength	12 weeks	Exercise intervention (Combined → Combined Exercise)	The efficacy of physiotherapy in patients after breast cancer surgery with axillary lymph node dissection → Handgrip strength showed a positive trend, but it was not markedly impaired postoperatively ($p = 0.08$).
93	Schwartz, A. L., et al. (2007)	N = 66	Mean Age (years):<br>- Aerobic group: 48.32 ± 12.6<br>- Resistance group: 50.1 ± 8.7<br>- Usual care group: 46.26 ± 9.8	Cardiorespiratory fitness, Muscular Strength	24 weeks	Exercise intervention (Home based → Combined Exercise, Resistance Exercise)	Aerobic exercise during chemotherapy for breast cancer survivors (BCS) → 12-minute walk ↑, Upper- and lower-body strength ↑<br>Resistance exercise during chemotherapy for BCS → Upper- and lower-body strength ↑, 12-minute walk: No change
94	So H. S., et al. (2006)	N = 32	Mean Age (years): 44	Cardiorespiratory fitness	6 weeks	Exercise intervention (Supervised → Steady-state Continuous Exercise)	Aerobic exercise with resistance bands during radiation therapy after mastectomy for breast cancer survivors (BCS) → Maximal oxygen uptake (VO_2 max) ↑
94	Kim, C. J., et al. (2006)	N = 41	Mean Age (years):<br>- Intervention group: 51.3 ± 6.7<br>- Control group: 48.3 ± 8.8<br>Mean BMI (kg/m²):<br>- Intervention group: 29.9 ± 5.5<br>- Control group: 28.0 ± 5.1	Cardiorespiratory fitness	8 weeks	Exercise intervention (Supervised, Home based → Steady-state Continuous Exercise)	8-week moderate-intensity aerobic exercise during adjuvant therapy for breast cancer survivors (BCS) → Peak oxygen uptake (VO_2 peak) ↑ (no group difference)
95	Cho et al. (2004)	N=55	Mean Age (years): 49.07 ± 7.58	Muscular Strength, Flexibility	10 weeks	Exercise intervention (Combined→ Stretching)	10-week stretching exercise after surgery → Shoulder ROM and grip strength ↑
97	Roberts S., et al. (2003)	N = 55	Mean Age (years):<br>- TPN group: 41.6 ± 6.6<br>- OD group: 45.6 ± 7.3	Muscular Strength	3.6 weeks	Non-exercise intervention (Nutrition)	HGS (Handgrip Strength) → No significant difference changes based on the treatment method.
98	Morimoto, T., et al. (2003)	N=33	Mean Age (years): 50.4 ± 9.8	Muscular Strength, Flexibility	4 weeks	Exercise intervention (Combined→ Combined Exercise)	4-week stretching and resistance training after surgery → Shoulder ROM ↑, grip strength (-)
99	Bendz, I., & Olsen, M. F. (2002)	N = 230	Mean Age (years):<br>- Group A: 58 ± 11<br>- Group B: 58 ± 11	Muscular Strength, Flexibility	2 weeks	Exercise intervention (Supervised → Stretching)	Immediate (A) vs. delayed (B) shoulder exercise after breast cancer surgery → shoulder mobility (flexion, abduction) ↑ faster in A grip strength ↓ slightly in both (n.s.) lymphoedema incidence (↔)
100	Chae, Y. R., & Choe, M. A. (2001).	N = 31	Mean Age (years): 44.03 ± 7.16	Cardiorespiratory fitness, Muscular Strength	8 weeks	Exercise intervention (Supervised →	8-week upper limb stretching + resistance + walking exercise (for BCP during radiotherapy)<br>→ VO_2 max & exercise duration ↑

						Steady-state Continuous Exercise)	→ Shoulder ROM (flexion, extension, abduction) ↑ in affected & unaffected sides
101	Segal R., et al. (2001)	N = 123	Mean Age (years):<br>- Control group: 50.3 ± 8.7<br>- Self-Directed Exercise group: 51.0 ± 8.7<br>- Supervised Exercise group: 51.4 ± 8.7	Cardiorespiratory fitness	26 weeks	Exercise intervention (Supervised, Home based → Steady-state Continuous Exercise)	Supervised exercise for breast cancer survivors (BCS) not receiving chemotherapy → Maximal oxygen uptake (VO_2 max) ↑ (compared to control)

↑ indicates increased or improved; ↓ indicates decreased or deteriorated; ↔ indicates no meaningful or statistically significant change.

Table S6. Studies Categorized by Phase: Post-treatment Phase (N = 199).

No.	Author (year)	Sample size	Participant Characteristics	Type of fitness outcome	Intervention duration	Intervention type	Key Results
1	Koevoets, E. W., et al. (2024)	N = 181	Mean Age (years):<br>- Intervention group: 51.7 ± 9.2<br>- Control group: 53.1 ± 8.9	Cardiorespiratory fitness	24 weeks	Exercise intervention (Combined → Combined Exercise)	Training group → VO_2 peak ↑ (significant) control group → no significant change
2	Zhao F. U., et al. (2024)	N = 60	Mean Age (years):<br>- Intervention group: 47.23 ± 6.03<br>- Control group: 48.37 ± 6.40<br>Mean BMI (kg/m^2):<br>- Intervention group: 29.27 (27.55–30.98)<br>- Control group: 28.70 (27.51–29.90)	Cardiorespiratory fitness, Muscular Strength	12 weeks	Exercise intervention (Home based → Combined Exercise)	12-week exercise-based weight management intervention for overweight/obese breast cancer survivors (BCS)<br>→ Left-hand grip strength ↑<br>→ Right-hand grip strength, Lower-body strength, Aerobic endurance no improvement
3	Ramadan A. M., et al. (2024)	N = 40	Mean Age (years):<br>- Control group: 51.05 ± 4.27<br>- Study group: 48.95 ± 5.05<br>Mean BMI (kg/m^2):<br>- Control group: 27.4 ± 1.34<br>- Study group: 28.22 ± 2.77	Muscular Strength	12 weeks	Exercise intervention (Supervised → Resistance Exercise, Combined Exercise)	12-week Kinesio taping (KT) + resistance training and resistance training→ Strength ↑ (hip flexors, knee extensors, knee flexors, ankle plantar flexors, ankle dorsiflexors)
4	Joaquim, A., et al. (2024)	N = 37	Mean Age (years): 57.9 ± 9.5<br>Mean BMI (kg/m^2): 29.7 ± 5.0	Cardiorespiratory fitness, Muscular Strength	16 weeks	Exercise intervention (Supervised → Combined Exercise)	16-week exercise intervention for breast cancer survivors (BCS) → VO_2 peak ↑ (during control phase only), Muscle strength ↑, Lower limb function ↑ (both phases)
5	Parsowith E. J., et al. (2024)	N = 19	Mean Age (years): 57.63 ± 10.48<br>Mean BMI (kg/m^2): 29.97 ± 5.79	Muscular Strength, Flexibility, Physical function	1 weeks	Non-exercise intervention (Creatine supplementation)	7-day creatine supplementation for breast cancer survivors (BCS) → Sit-to-Stand power, Isometric strength, Isokinetic strength, 10-repetition maximum (10RM): No change (no group difference)

6	Moro T., et al. (2024)	N = 31	Mean Age (years): 57.88 ± 7.88<br>Mean BMI (kg/m²): 27.86 ± 6.38	Cardiorespiratory fitness, Muscular Strength	12 weeks	Exercise intervention (Supervised, Home based → etc, Circuit Training)	Dragon Boat activity for breast cancer survivors (BCS) → 6-minute walk test ↑, 30-second chair stand test ↑, VO_2 peak ↑, Grip strength: No change
7	Liu, L., et al. (2024)	N = 36	Mean Age (years): Not reported<br>Mean BMI (kg/m²): 24.25 ± 3.48	Muscular Strength, Flexibility	12 weeks	Exercise intervention (Supervised → etc.)	12-week Yogalates intervention for breast cancer survivors (BCS) → Grip strength ↑ (both affected and unaffected sides)
8	Andrioti, A., et al. (2023)	N = 13	Mean Age (years): 58.31 ± 3.13<br>Mean BMI (kg/m²): 25.68 ± 0.62	Cardiorespiratory fitness, Muscular Strength	8 weeks	Exercise intervention (Home based → Combined Exercise)	8-week exercise intervention → cardiorespiratory fitness (CRF) ↑, muscle function ↑
9	Calonego C. et al. (2023)	N=19	Mean Age (years): 53.89 ± 11.23<br>BMI categories: <25: 15.8%, 25–29.9: 26.3%, ≥30: 57.9%	Cardiorespiratory fitness, Muscular Strength, Flexibility	8 weeks	Exercise intervention (Supervised → Combined Exercise)	Multiple-set (MS) group → time to exhaustion ↑ (higher training volume → additional aerobic benefit)<br>Single-set (SS) & MS groups → knee extension 1-repetition maximum (1RM) ↑
10	Soriano-Maldonado A,, et al. (2023)	N = 60	Mean Age (years):<br>- Exercise group: 52.6 ± 8.8<br>- Control group: 52.0 ± 9.4<br>Mean BMI (kg/m²):<br>- Exercise group: 27.4 ± 4.2<br>- Control group: 26.3 ± 5.3	Cardiorespiratory fitness, Muscular Strength	12 weeks	Exercise intervention (Combined → Combined Exercise)	After 12weeks supervised resistance + home-based PA → Muscular strength (upper/lower/full body) ↑ Cardiorespiratory fitness not significant
11	Tam R. M., et al. (2023)	N = 20	Mean Age (years): 64.5 ± 9.4<br>Mean BMI (kg/m²): 30.4 ± 4.5	Cardiorespiratory fitness, Muscular Strength, Physical function	12 weeks	Non-exercise intervention (Behavioral counseling and coaching)	An intervention to reduce sitting time in breast cancer survivors → Physical function ↑ (4MWT [4-Meter Walk Test], TUG [Timed Up and Go], Chair Stand)
12	Damato, A., et al. (2023)	N = 35	Mean Age (years): 59.6 ± 6.6	Muscular Strength	6 weeks	Exercise intervention (Home based → Resistance Exercise)	6-week moderate to high-intensity RE (Resistance Exercise) → Muscle strength ↑
13	Farajivafa, V., et al. (2023)	N = 89	Mean Age (years): 45.8 ± 8.2<br>Mean BMI (kg/m²): 28.5 ± 4.9	Cardiorespiratory fitness	12 weeks	Exercise intervention (Home based → Steady-state Continuous Exercise)	12-week home-based exercise intervention for breast cancer survivors → VO_2 peak ↑, Cardiorespiratory fitness ↑
14	França-Lara, É. G., et al. (2023)	N = 107	Mean Age (years):<br>- Adherent group: 43.8 ± 8.8<br>- Non-Adherent group: 45.2 ± 8.0<br>Mean BMI (kg/m²):<br>- Adherent group: 28.3 ± 4.6<br>- Non-Adherent group: 25.1 ± 3.9	Cardiorespiratory fitness, Muscular Strength	36 weeks	Exercise intervention (Home based → Combined Exercise)	Exercise adherence in breast cancer survivors (BCS) → VO_2 max ↑, Muscular endurance (repetition of arm abduction) ↑ (greater than non-adherence group)

15	Guloglu, S., et al. (2023)	N = 66	Mean Age (years):<br>- PNF group: 46.0 ± 7.7<br>- PRT group: 48.8 ± 9.8<br>- Control group: 44.2 ± 7.0<br>Mean BMI (kg/m²):<br>- PNF group: 28.8 ± 4.8<br>- PRT group: 28.3 ± 3.5<br>- Control group: 27.2 ± 4.8	Muscular Strength	8 weeks	Non-exercise intervention (Neuromuscular facilitation)	8week proprioceptive neuromuscular facilitation (PNF) and progressive resistance training (PRT) for breast cancer survivors (BCS) after axillary lymph node dissection (ALND) → Strength ↑, Power ↑, Endurance ↑<br>→ PNF ↑, PRT ↑, Control: No change<br>→ Function ↑ in PNF (greater than PRT)
16	Park Y. J., et al. (2023)	N = 20	Mean Age (years):<br>- PREG group: 58.86 ± 3.28<br>- SREG group: 60.29 ± 5.09	Muscular Strength	6 weeks	Exercise intervention (Supervised, Home based → Resistance Exercise, CDPT)	Progressive resistance exercise (PRE) with complex decongestive Physical therapy (CDPT) for breast cancer survivors (BCS) with lymphedema → Grip strength ↑
17	Natalucci V., et al. (2023)	N = 30	Mean Age (years): 53.6 ± 7.6<br>Mean BMI (kg/m²): 26.17 ± 4.97	Cardiorespiratory fitness	12 weeks	Exercise intervention (Combined → Steady-state Continuous Exercise)	Home-based lifestyle intervention during COVID-19 for breast cancer survivors (BCS) → Maximal oxygen uptake (VO_2 max) ↑ (3 and 24 months), Physical activity level (PAL) ↑ (3 months)
18	Spence et al. (2022)	N = 60	Mean Age (years): 50.1 ± 9.0<br>Mean BMI (kg/m²): 28.9 ± 6.2	Cardiorespiratory fitness, Muscular Strength	12 weeks	Exercise intervention (Combined → Combined Exercise)	Effect of Physical activity (PA), self-efficacy, upper-body strength, and lower-body strength the high-supervision group > low-supervision group
19	Kerri M. Winters-Stone et al. (2022)	N = 117	Mean Age (years): 70.9 ± 5.1<br>Mean BMI (kg/m²): 29.2 ± 5.9	Cardiorespiratory fitness, Muscular Strength, Flexibility, Physical function	48 weeks	Exercise intervention (Combined → Combined Exercise)	The resistance exercise training (RET) group showed significant improvements in upper and lower body strength and aerobic capacity compared to the control group (FLEX)↑, but there was no significant difference in flexibility.
20	Short.T et al. (2022)	N = 59	Mean Age (years): 61 ± 9<br>Mean BMI (kg/m²): 26.8	Cardiorespiratory fitness, Muscular Strength, Flexibility	12 weeks	Exercise intervention (Supervised → Combined Exercise)	The exercise-based cancer rehabilitation (ExCR) program significantly improved various fitness variables in breast cancer survivors ↑
21	Jessica.M et al. (2022)	N = 30	Mean Age (years):<br>- Control group: 60.1 ± 8.5<br>- Higher intensity PA group: 57.7 ± 10.4<br>- Lower intensity PA group: 57.7 ± 9.3<br>Mean BMI (kg/m²):<br>- Control group: 27.7 ± 8.8<br>- Higher intensity PA group: 31.1 ± 8.4	Cardiorespiratory fitness	12 weeks	Exercise intervention (Home based → Steady-state Continuous Exercise)	This suggests that low-intensity Physical activity (PA) may be more sustainable and enjoyable for breast cancer survivors and may lead to increased adherence.<br>Social support and Physical fitness levels may influence the effectiveness of PA interventions.

			- Lower intensity PA group: 28.7 ± 4.9				
22	Jacquinot Q et al. (2022)	N = 89	Median Age (years): 51.0 (Range: 43.4–55.8)	Cardiorespiratory fitness	12 weeks	Exercise intervention (Supervised → Steady-state Continuous Exercise)	12-week exercise program → training group: cardiopulmonary function ↑ (peak oxygen uptake [VO_2 peak], maximal power, VO_2/HR), confirming effectiveness in improving cardiopulmonary fitness
23	Kokkonen, K. et al. (2022)	N = 446	Mean Age (years): 53 ± 8<br>Mean BMI (kg/m²): 25.9 ± 4.1	Cardiorespiratory fitness	48 weeks	Exercise intervention (Combined → Steady-state Continuous Exercise)	Higher leisure-time Physical activity (LTPA) was associated with higher fitness level ($p < 0.001$), and 2 km walk test time showed a negative correlation with LTPA level ($\beta = -0.18$, $p < 0.001$)
24	Martins F. M. et al. (2022)	N = 26	Mean Age (years):<br>- CG group: 57.7 ± 8.8<br>- RT group: 52.1 ± 10.1<br>Mean BMI (kg/m²):<br>- CG group: 26.2 ± 6.4<br>- RT group: 26.2 ± 3.3	Cardiorespiratory fitness, Muscular Strength	12 weeks	Exercise intervention (Supervised → Resistance Exercise)	Resistance training → Physical activity ↑, muscular strength ↑ (1-repetition maximum [1RM]), endurance ↑, fatigue ↓, Physical function ↑ ($p = 0.05$) strength positively correlated with critical torque (CT), work capacity above critical torque (W'), maximal voluntary contraction (MVC), and 6-minute walk test (6MWT)
25	Dahhak A., et al. (2022)	N = 20	Mean Age (years):<br>- Intervention group: 51 ± 5<br>- Control group: 55 ± 9	Cardiorespiratory fitness, Muscular Strength	12 weeks	Non-exercise intervention (Adjunctive inspiratory muscle training)	Adjunctive inspiratory muscle training (IMT) → peak exercise time ↑ handgrip strength and 6-minute walk test (6MWT): no significant change
26	Tami-Maury, I. M., et al. (2022)	N = 188	Mean Age (years):<br>- Not Enrolled group: 56.3 ± 11.6<br>- Enrolled group: 59.6 ± 10.7	Cardiorespiratory fitness, Muscular Strength	12 weeks	Exercise intervention (Combined → Combined Exercise)	After 12 weeks of program → Physical function ↑ (walking distance, lower body strength tests) ($p < 0.01$).
27	Wagoner C. W., et al. (2022)	N = 56	Mean Age (years):<br>- EBCS group: 53.9 ± 11.9<br>- CON group: 53.8 ± 8.4	Cardiorespiratory fitness, Muscular Strength, Physical function	16 weeks	Exercise intervention (Supervised → Combined Exercise)	Community-based exercise in early breast cancer survivors (EBCS)<br>→ Physical function ↑, Fatigue ↓ (6-minute walk distance ↑ significant)<br>→ VO_2 peak, upper & lower body strength: no significant change
28	Song M. J., et al. (2022)	N = 13	Mean Age (years): 52.93 ± 4.97<br>Mean BMI (kg/m²): 25.07 ± 2.75	Cardiorespiratory fitness, Muscular Strength	8 weeks	Exercise intervention (Supervised → Resistance Exercise, Steady-state Continuous Exercise)	Music-based combined exercise for breast cancer survivors (BCS) → Hand grip strength ↑, 6-minute walk test (6MWT) ↑
29	Sagarra-Romero L., et al. (2022)	N = 21	Mean Age (years): 55.53 ± 6.7<br>Mean BMI (kg/m²): 25.5 ± 3.8	Cardiorespiratory fitness, Muscular Strength, Physical function	16 weeks	Exercise intervention (Combined → Combined Exercise)	Online home-based exercise during COVID-19 lockdown for breast cancer survivors (BCS) → Upper limb strength ↑, Lower limb strength ↑, Cardiorespiratory fitness ↑

30	Robbins R. N., et al. (2022)	N = 32	Mean Age (years):<br>- RT group: 61.6 ± 1.5<br>- CBCT group: 67.2 ± 1.2<br>Mean BMI (kg/m²):<br>- RT group: 32.7 ± 1.4<br>- CBCT group: 28.9 ± 4.5	Cardiorespiratory fitness, Muscular Strength, Physical function	12 weeks	Exercise intervention (Supervised → Resistance Exercise)	12-week resistance training (RT) for breast cancer survivors (BCS) → 6-minute walk distance (6MWD) ↑, Chair stand test ↑, Hand grip strength ↑ (no group difference vs. cognitive behavioral training group)
31	Koevoets, E. W., et al. (2022)	N = 181	Mean Age (years):<br>- Intervention group: 52.1 ± 8.6<br>- Control group: 52.5 ± 8.7	Cardiorespiratory fitness	24 weeks	Exercise intervention (Combined → Combined Exercise, High-Intensity Interval Training)	Exercise intervention for breast cancer survivors (BCS) → Maximal oxygen uptake (VO_2 peak) ↑ (vs. control)
32	Kozanoglu, E., et al. (2022)	N = 62	Mean Age (years):<br>- Group I: 50.0 (46–66)<br>- Group II: 56.0 (49–62)	Muscular Strength, Flexibility	4 weeks	Non-exercise intervention (Ipc and low-level laser therapy)	Intermittent pneumatic compression (IPC) + low-level laser therapy (LLLT) vs. IPC alone for breast cancer survivors (BCS) → Grip strength ↑ in both groups (vs. baseline) No group difference
33	Lee, K. J., & An, K. O. (2022)	N = 30	Mean Age (years):<br>- HCREG group: 54.7 ± 5.1<br>- CG group: 55.4 ± 4.3<br>Mean BMI (kg/m²):<br>- HCREG group: 24.2 ± 3.9<br>- CG group: 23.6 ± 3.0	Muscular Strength, Flexibility, Physical function	12 weeks	Exercise intervention (Supervised → Circuit Training)	12-week supervised high-intensity circuit resistance exercise for breast cancer survivors (BCS) → Muscle strength ↑, Agility ↑, Balance ↑
34	Owusu C, et al. (2022)	N = 213	Mean Age (years): 71.85 ± 5.88<br>Mean BMI (kg/m²): 30.13 ± 6.67	Cardiorespiratory fitness, Muscular Strength, Physical function	52 weeks	Exercise intervention (Combined → Combined Exercise)	Combined aerobic and resistance exercise for older breast cancer survivors (BCS) → Physical function ↑ (6-minute walk test, 4-meter gait speed) Greater effect in African American vs. Non-Hispanic White
35	Ochi E., et al. (2022)	N = 50	Age Range (years): 20–59	Cardiorespiratory fitness, Muscular Strength	12 weeks	Exercise intervention (Home based → High-Intensity Interval Training)	Home-based high-intensity interval training (HIIT) for early-stage breast cancer survivors (BCS) → Maximal oxygen uptake (VO_2 peak) ↑, Leg press one-repetition maximum (1RM) ↑
36	Naderi M., et al. (2022)	N = 33	Mean Age (years): 47.90 ± 7.95	Muscular Strength	12 weeks	Exercise intervention (Supervised → etc.)	12-week yoga plus vitamin D supplementation for breast cancer survivors (BCS)<br>→Y+LVD group (yoga + low-dose vitamin D)→ Grip strength ↑<br>→Y+HVD group (yoga + high-dose vitamin D)→ Grip strength ↑<br>→HVD group (high-dose vitamin D only)→ No change
37	Naczk A., et al. (2022)	N = 24	Mean Age (years): 66.2 ± 10.6	Muscular Strength, Flexibility	6 weeks	Exercise intervention (Supervised → Resistance Exercise)	Inertial training after mastectomy for breast cancer survivors (BCS) → Muscle strength ↑ (shoulder flexors, extensors, abductors, adductors)

38	Macdonald, E. R., et al. (2022)	N = 29	Mean Age (years): 53.0 ± 10.6<br>Mean BMI (kg/m²): 24.9 ± 4.5	Cardiorespiratory fitness, Muscular Strength, Flexibility	16 weeks	Exercise intervention (Supervised, Home based → etc.)	16-week classical ballet intervention for breast cancer survivors (BCS) →Shoulder joint range of motion (ROM) ↑, sedentary lifestyle ↓, muscle strength not significant
39	Lippi, L., et al. (2022)	N = 37	Mean Age (years):<br>- PA Intervention group: 53.42 ± 9.08<br>- Control group: 52.86 ± 10.38<br>Mean BMI (kg/m²):<br>- PA Intervention group: 27.51 ± 5.04<br>- Control group: 28.56 ± 5.50	Muscular Strength	1 weeks	Exercise intervention (Supervised → Resistance Exercise)	US-guided corticosteroid injections+rehabilitation program → grip strength ↑ at after 3 month follow up
40	Basha at el. (2021)	N = 60	Mean Age (years):<br>- Xbox Kinect group: 48.83 ± 7.0<br>- Resistance group: 52.07 ± 7.48<br>Mean BMI (kg/m²):<br>- Xbox Kinect group: 24.84 ± 4.81<br>- Resistance group: 30.57 ± 4.99	Muscular Strength	8 weeks	Exercise intervention (Supervised → Combined Exercise, Resistance Exercise)	Xbox Kinect group → ↓ pain, ↑ upper limb function, ↑ shoulder ROM, ↑ health-related quality of life<br>Resistance exercise group → ↑ shoulder strength, ↑ handgrip strength
41	Samhan, A. F., et al. (2021)	N = 63	Mean Age (years):<br>- HIIT group: 49.7 ± 8.9<br>- Control group: 48.9 ± 7.7	Cardiorespiratory fitness	8 weeks	Exercise intervention (Supervised → High-Intensity Interval Training)	High-intensity interval training (HIIT) group → peak oxygen uptake (VO_2 peak) ↑ (+3.77 mL·kg^{-1}·min^{-1}, p = 0.002)
42	Basoglu C., et al. (2021)	N = 36	Mean Age (years):<br>- CDT group: 53.4 ± 8.3<br>- KT group: 53.7 ± 8.6<br>Mean BMI (kg/m²):<br>- CDT group: 26.7 ± 5.1<br>- KT group: 29.7 ± 4.4	Muscular Strength, Flexibility	4 weeks	Non-exercise intervention (Complex decongestive therapy)	CDT → grip strength ↑ (only at the 1st month follow-up)<br>KT → grip strength ↑ (both after treatment and at follow-up with no significant difference between the groups.
43	Bell R. A., et al. (2021)	N = 20	Mean Age (years):<br>- HIIT group: 49 ± 4<br>- MICT group: 51 ± 5<br>Mean BMI (kg/m²):<br>- HIIT group: 25.1 ± 5.6<br>- MICT group: 27.1 ± 6.8	Cardiorespiratory fitness	12 weeks	Exercise intervention (Supervised → Combined Exercise)	High-intensity interval training (HIIT) & moderate-intensity continuous training (MICT) → peak oxygen uptake (VO_2 peak) ↑<br>HIIT → peak workload & minute ventilation ↑ no significant between-group difference in overall cardiovascular fitness (↔)
44	Hasenöhrl, T., et al. (2021)	N=10	Mean Age (years): 52.0 ± 5.4	Cardiorespiratory fitness, Muscular Strength	10 weeks	Exercise intervention (Supervised → Combined Exercise)	Physical performance → strength & mobility ↑, affected arm elbow flexion ↔
45	Lee, J. T., et al. (2021)	N = 56	Mean Age (years): 54 ± 11<br>BMI distribution: 24% Normal 52%	Cardiorespiratory fitness, Physical function	16 weeks	Exercise intervention (Supervised → Combined Exercise)	After 16 weeks → Peak oxygen uptake (VO_2 peak) ↑, 6-minute walk test (6MWT) ↑, Timed Up and Go (TUG) ↑ in both breast cancer survivors (BCS) & controls (CTLs)

			Overweight, 9% Obese, 15% Obese II				
46	Corum, M., et al. (2021)	N = 50	Mean Age (years):<br>- Group 1: 51.9 ± 8.1<br>- Group 2: 54.7 ± 7.8<br>Mean BMI (kg/m²):<br>- Group 1: 28.3 ± 5.5<br>- Group 2: 28.0 ± 4.3	Muscular Strength	4 weeks	Exercise intervention (Supervised → Resistance Exercise)	4-week standard CDT (Complex Decongestive Therapy) + exercise<br>→ Grip strength ↑ (in both pain, no-pain groups)
47	Dieli-Conwright, C. M., et al. (2021)	N = 100	Mean Age (years):<br>- HBCS group: 46.8 ± 10.2<br>- NHBCS group: 55.7 ± 10.5<br>Mean BMI (kg/m²):<br>- HBCS group: 35.1 ± 6.1<br>- NHBCS group: 33.1 ± 5.4	Cardiorespiratory fitness, Muscular Strength	16 weeks	Exercise intervention (Supervised → Combined Exercise)	16-week aerobic + resistance exercise<br>→ All Physical fitness variables ↑ Hispanics ↑ > non-Hispanics<br>→ 28-week follow-up: All variables ↑ vs. baseline
48	Uth J., et al. (2021)	N = 68	Mean Age (years):<br>- Football group: 47.4 ± 9.4<br>- Control group: 50.0 ± 9.3<br>Mean BMI (kg/m²):<br>- Football group: 25.5 ± 4.9<br>- Control group: 26.4 ± 4.6	Muscular Strength, Flexibility, Physical function	48 weeks	Exercise intervention (Supervised → Steady-state Continuous Exercise)	1-year Football Fitness training in women treated for stage I~III breast cancer survivor (BCS)<br>→ Leg muscle strength ↑, Postural balance ↓ (but ↓ less than control)
49	Wang L. F., et al. (2021)	N = 60	Mean Age (years):<br>- Immediate intervention group: 55.6 ± 8.2<br>- Control group: 56.0 ± 11.5<br>Mean BMI (kg/m²):<br>- Immediate intervention group: 31.5 ± 7.0<br>- Control group: 30 ± 6.4	Cardiorespiratory fitness, Muscular Strength, Flexibility	30 weeks	Exercise intervention (Supervised → Combined Exercise)	Customized exercise intervention – 2minute walk test (2MWT), Upper body flexibility and strength – Endurance ↑, Flexibility ↑, Strength ↑
50	Samhan A. F et al. (2021)	N = 63	Mean Age (years):<br>- HIIT group: 49.7 ± 8.9<br>- Control group: 48.9 ± 7.7<br>Mean BMI (kg/m²):<br>- HIIT group: 33.22 ± 1.29<br>- Control group: 33.48 ± 1.45	Cardiorespiratory fitness	8 weeks	Exercise intervention (Supervised → High-Intensity Interval Training)	High-intensity interval training (HIIT) for breast cancer survivors (BCS) → Peak oxygen uptake (VO_2 peak) ↑, No group difference
51	Reis .A. D., et al. (2021)	N = 28	Mean Age (years):<br>- Face-to-face group: 61.91 (56.87–66.95)<br>- Home-based group: 55.0 (50.27–	Cardiorespiratory fitness	24 weeks	Exercise intervention (Supervised, Home based → Steady-state Continuous Exercise)	Aerobic exercise combined with food orientation during breast cancer treatment → Cardiorespiratory fitness (CRF) ↑

			60.23)<br>Mean BMI (kg/m²):<br>- Face-to-face group: 30.98 (27.53–34.42)<br>- Home-based group: 29.17 (26.71–31.63)				
52	Khedmati Zare, V., et al. (2021)	N = 30	Mean Age (years): 47.90 ± 7.95	Cardiorespiratory fitness, Flexibility	12 weeks	Exercise intervention (Supervised → etc.)	12-week yoga plus vitamin D supplementation for breast cancer survivors (BCS) → Aerobic fitness ↑ in Y+LD group (yoga + low-dose vitamin D) and Y+HD group (yoga + high-dose vitamin D) No change in HD group (high-dose vitamin D only)
53	Ortiz A., et al. (2021)	N = 89	Mean Age (years): 55.4 ± 10<br>Mean BMI (kg/m²): 31 ± 6.5	Cardiorespiratory fitness, Muscular Strength, Flexibility, Physical function	16 weeks	Exercise intervention (Home based → Combined Exercise)	16-week home-based intervention for breast cancer survivors (BCS) → Physical outcomes: No change (no group difference)
54	Natalucci V., et al. (2021)	N = 30	Mean Age (years): 52.6 ± 7.6<br>Mean BMI (kg/m²): 26.0 ± 5.0	Cardiorespiratory fitness	12 weeks	Exercise intervention (Combined → Steady-state Continuous Exercise)	Home-based lifestyle intervention (LI) during COVID-19 lockdown for breast cancer survivors (BCS) → Cardiorespiratory fitness ↑, Physical activity level ↑
55	Kang et al. (2021)	N=59	Mean Age (years): 55.1 ± 6.6	Flexibility	8 weeks	Exercise intervention (Supervised → Steady-state Continuous Exercise)	8-week swimming exercise in breast cancer survivors → Shoulder ROM ↑
56	Namadev Jare, S.,et al. (2021)	N=40	Age Distribution (%):<br>- ≥50 years: 57.5% (23/40)<br>- <50 years: 42.5% (17/40)	Flexibility	8 weeks	Exercise intervention (Supervised → Resistance Exercise)	8-week resistance training in breast cancer survivors → shoulder ROM ↑
57	Hooshmand Moghadam, B., et al. (2021)	N=30	Mean Age (years): 56.9 ± 13.2<br>Mean BMI (kg/m²): 28.2 ± 2.2	Cardiorespiratory fitness	12 weeks	Exercise intervention (Supervised→ High-Intensity Interval Training, Steady-state Continuous Exercise)	12-week HIIT or MICT intervention in breast cancer survivors → Vo2peak, (upper/lower) body strength ↑
58	Dong et al. (2020)	N = 60	Mean Age (years): 51.2 ± 7.9<br>Mean BMI (kg/m²): 24.57 ± 2.60	Cardiorespiratory fitness, Muscular Strength	12 weeks	Exercise intervention (Combined → Combined Exercise)	Intervention group → Physical capability ↑
59	Ashley L. Artese et al. (2020)	N = 44	Mean Age (years): 60.3 ± 8.3<br>Mean BMI (kg/m²): 29.6 ± 6.7	Muscular Strength, Flexibility	24 weeks	Exercise intervention (Supervised →	Functional impact training (FIT) group: significant ↑ in upper and lower body strength.

						Combined Exercise, etc.)	Yin yoga group: slight ↓ in upper body strength overall ↔ lower body strength.
60	Pegueros-Pérez A., et al. (2020).	N = 30	Mean Age (years): 52.0<br>Mean BMI (kg/m²): 28.9	Cardiorespiratory fitness	24 weeks	Exercise intervention (Supervised → Combined Exercise)	After 25 weeks → significant improvements in fitness and body composition: METs ↑, maximal oxygen uptake (VO_2 max) ↑, BMI ↓, fat mass ↓, muscle mass ↑, waist circumference ↓
61	Tastaban E. et al. (2020)	N = 76	Mean Age (years):<br>- Group 1 CDT: 55.0 (48.0–58.0)<br>- Group 2 CDT+IPC: 53.0 (43.0–58.0)<br>Mean BMI (kg/m²):<br>- Group 1 CDT: 28.7 (27.9–29.6)<br>- Group 2 CDT+IPC: 28.7 (27.9–29.6)	Muscular Strength	4 weeks	Exercise intervention (Supervised → Stretching)	Baseline to 4 weeks post-treatment → grip strength slight ↑, not statistically significant (p = 0.128)
62	Pagola. I., et al. (2020)	N = 23	Mean Age (years):<br>- Moderate-intensity group: 51 ± 6<br>- High-intensity group: 47 ± 7	Cardiorespiratory fitness, Muscular Strength	16 weeks	Exercise intervention (Supervised, Home based → Combined Exercise)	High- and moderate-intensity exercises → lower limb strength ↑ no effect on handgrip strength or cardiorespiratory fitness
63	Khan, S., et al. (2020)	N = 60	Age Range (years): 35–55	Cardiorespiratory fitness	6 weeks	Exercise intervention (Supervised → Steady-state Continuous Exercise, Resistance Exercise)	Both aerobic and resistance exercise interventions → maximal oxygen uptake (VO_2 max) ↑
64	Mascherini G. et al. (2020)	N=42	Mean Age (years): 52.0 ± 10.1<br>Mean BMI (kg/m²): 27.3 ± 4.2	Cardiorespiratory fitness, Muscular Strength, Flexibility	24 weeks	Exercise intervention (Home based → Combined Exercise)	Unsupervised exercise program → 6MWT ↑, handgrip strength ↑ (both arms), lower limb strength ↑
65	Chang J. S. et al. (2020)	N=46	Mean Age (years):<br>- Control group: 50.0 ± 6.1<br>- Exercise group: 51.4 ± 7.5<br>Mean BMI (kg/m²):<br>- Control group: 24.6 ± 4.4<br>- Exercise group: 22.7 ± 2.6	Cardiorespiratory fitness, Muscular Strength, Flexibility, Physical function	12 weeks	Exercise intervention (Supervised → Combined Exercise)	After 12 weeks aerobic + resistance exercise → muscular strength ↑, endurance ↑, aerobic fitness ↑, flexibility ↑ in breast cancer survivors
66	Toohey K., et al. (2020)	N = 17	Mean Age (years): 62 ± 8<br>Mean BMI (kg/m²): 26.30 ± 4.39	Cardiorespiratory fitness	12 weeks	Exercise intervention (Supervised → High-Intensity Interval Training, Steady-state Continuous Exercise)	After 12 weeks HIIT (High-Intensity Interval Training) → Cardiovascular fitness ↑ in BCS (Breast Cancer Survivors) vs. CMIT (Continuous aerobic training)

67	De Groef, A., et al. (2020)	N = 50	Mean Age (years): 53.13 ± 1.93<br>Mean BMI (kg/m²): 26.43 ± 2.81	Muscular Strength	24 weeks	Exercise intervention (Combined → Combined Exercise)	Single botulinum toxin A (BTX-A) injection → No significant difference was observed in grip strength bewtween groups
68	Uth J., et al. (2020)	N = 68	Mean Age (years): 47.4 ± 9.4<br><br>Mean BMI (kg/m²):<br>- Football group: 25.5 ± 4.9<br>- Control group: 26.4 ± 4.6	Cardiorespiratory fitness	48 weeks	Exercise intervention (Supervised → Steady-state Continuous Exercise)	1-year Football Fitness training in breast cancer survivors (BCS) → No improvement in cardiorespiratory fitness (CRF), blood pressure (BP), blood lipids, fat mass, or resting heart rate (HRrest).
69	Viskochil R., et al. (2020)	N = 15	Mean Age (years): 59.9 ± 9.2<br>Mean BMI (kg/m²): 27.6 ± 5.4	Cardiorespiratory fitness	12 weeks	Exercise intervention (Supervised → Steady-state Continuous Exercise)	12-week supervised aerobic exercise intervention →Estimated peak oxygen uptake (VO_2 peak) ↑
70	Zeng N., et al. (2020)	N = 95	Mean Age (years): 44.81 ± 7.94<br>Mean BMI (kg/m²): 22.18 ± 3.48	Physical function	48 weeks	Exercise intervention (Home based → Steady-state Continuous Exercise)	12-month personalized exercise program for Chinese breast cancer survivors (BCS)<br>→ Aerobic endurance ↑, Lower-body flexibility ↑, Strength and upper-body flexibility no improvement
71	Gavala-González, J., et al. (2020)	N = 30	Mean Age (years): 53.70 ± 7.88<br>Mean BMI (kg/m²): 26.34 ± 3.78	Cardiorespiratory fitness, Muscular Strength, Flexibility	12 weeks	Exercise intervention (Supervised → etc.)	12-week rowing training for breast cancer survivors (BCS) → Strength ↑, Aerobic capacity ↑, Flexibility ↑, Heart rate ↓
72	Hanuszkiewicz, J. M., et al. (2020)	N = 39	Mean Age (years):<br>- CG group: 59.4 ± 7.47<br>- SG group: 58.2 ± 7.26	Muscular Strength	8 weeks	Exercise intervention (Supervised → Steady-state Continuous Exercise)	8-week Nordic walking intervention for breast cancer survivors (BCS) → Trunk muscle total work ↑, Trunk muscle power ↑
73	Invernizzi, M., et al. (2020)	N = 36	Mean Age (years): 55.17 ± 7.76<br>Mean BMI (kg/m²): 25.15 ± 5.52	Cardiorespiratory fitness, Muscular Strength, Physical function	4 weeks	Exercise intervention (Supervised → Combined Exercise)	Four weeks and 3 months later, significant improvements were observed in all fitness variables (HGS, SPPB, 10MWT, 6MWT) ($p < 0.05$).↑
74	Scott, J. M., et al. (2020)	N = 174	Mean Age (years): 58 ± 9<br>Mean BMI (kg/m²): 29.5 ± 5.6	Cardiorespiratory fitness	16 weeks	Exercise intervention (Supervised → Steady-state Continuous Exercise)	Short-term exercise training for breast cancer survivors (BCS) → Cardiorespiratory fitness ↑ (regardless of exercise dosing schedule)
75	Santagnello S. B. et al. (2020)	N = 26	Mean Age (years):<br>- CT group: 59.0 ± 9.2<br>- RE group: 52.1 ± 10.1<br>Mean BMI (kg/m²):<br>- CT group: 25.5 ± 6.5<br>- RE group: 26.2 ± 3.3	Cardiorespiratory fitness, Muscular Strength, Physical function	12 weeks	Exercise intervention (Supervised → Resistance Exercise)	Resistance exercise for breast cancer survivors (BCS) → Strength ↑, Power ↑, Walk time ↓, Sit-to-stand performance ↑ (vs. control), 6-minute walk test (6MWT) ↑, Timed Up and Go (TUG) ↓

76	Jones, L. M., et al. (2020)	N = 51	Mean Age (years):<br>- Exercise group: 55.8 ± 7.2<br>- Control group: 55.9 ± 7.1<br>Mean BMI (kg/m²):<br>- Exercise group: 27.8 ± 5.5<br>- Control group: 27.5 ± 4.8	Cardiorespiratory fitness, Muscular Strength	12 weeks	Exercise intervention (Supervised → Combined Exercise)	12-week circuit resistance training for breast cancer survivors (BCS) → Estimated maximal oxygen uptake (VO_2 max) ↑, Walking distance ↑, Bench one-repetition maximum (1RM) ↑, Knee extensor strength ↑ (vs. control)
77	Lee, J., & Vicil, F. (2020)	N = 38	Mean Age (years):<br>- Exercise group: 52.63 ± 7.75<br>- Control group: 53.53 ± 7.43<br>Mean BMI (kg/m²):<br>- Exercise group: 24.30 ± 2.23<br>- Control group: 24.24 ± 3.36	Cardiorespiratory fitness, Muscular Strength, Flexibility	8 weeks	Exercise intervention (Supervised → Combined Exercise)	The exercise group showed significant improvements in handgrip strength, cardiorespiratory fitness, shoulder flexibility ($p<0.01$).↑
78	Nouri R., et al. (2020)	N = 75	Mean Age (years):<br>- Control group: 46 ± 7.15<br>- Resistance training group: 46 ± 5.82<br>- Combined training group: 48.9 ± 7.06<br>Mean BMI (kg/m²):<br>- Control group: 25.17 ± 3.7<br>- Resistance training group: 25.7 ± 3.9<br>- Combined training group: 24.6 ± 3.5	Physical function	6 weeks	Exercise intervention (Supervised → Combined Exercise)	6-week Thera-Band resistance and combined training for breast cancer survivors (BCS) → Dynamic balance: No change, Static balance: No change
79	Omar, M. T., et al. (2020)	N=60	Mean Age (years): 56.9 ± 13.2<br>Mean BMI (kg/m²): 28.2 ± 2.2	Flexibility	8 weeks	Exercise intervention (Supervised→ Combined Exercise)	8-week resistance training with compression garment in breast cancer survivors → shoulder ROM ↑
80	Cerulli, C., et al. (2019)	N=14	Mean Age (years): 58.3 ± 5.2	Cardiorespiratory fitness, Muscular Strength, Flexibility	16 weeks	Exercise intervention (Supervised → etc.)	4-month Dancing with Health (DWH) intervention → functional ability ↑ (6-minute walk test [6MWT]) Physical strength & health status ↑
81	Coletta, A. M., et al. (2019)	N = 44	Mean Age (years): 63.90 ± 8.8<br>Mean BMI (kg/m²): 30.87 ± 5.7	Cardiorespiratory fitness	12 weeks	Exercise intervention (Supervised → Steady-state Continuous Exercise)	High-intensity interval training (HIIT) → cardiorespiratory fitness (CRF) ↑ vs. moderate-intensity continuous training (MICT) & usual care (UC) in women at high risk for breast cancer
82	Sweeney F. C., et al. (2019)	N = 100	Mean Age (years):<br>- Exercise group: 52.8 ± 10.6<br>- Usual care group: 50.6 ± 10.1<br>Mean BMI (kg/m²):	Muscular Strength, Flexibility	16 weeks	Exercise intervention (Supervised → Combined Exercise)	A supervised 16week aerobic and resistance exercise intervention designed to improve metabolic syndrome → Upper extremity functional mobility & strength ↑

			- Exercise group: 33.5 ± 5.7<br>- Usual care group: 33.7 ± 5.2				
83	Tantawy S. A., et al. (2019)	N = 66	Mean Age (years):<br>- KT group: 54.3 ± 4.16<br>- PG group: 55.15 ± 3.27<br>Mean BMI (kg/m²):<br>- KT group: 28.4 ± 2.7<br>- PG group: 29.1 ± 2.5	Muscular Strength	3 weeks	Non-exercise intervention (Kinesio taping, pressure garment therapy)	After 3weeks Kinesio taping intervention (for BCS [Breast Cancer Survivors] with lymphedema post-mastectomy)→ Handgrip strength ↑ (vs. pressure garment)
84	Dong, X.., et al. (2019)	N = 60	Mean Age (years):<br>- Intervention group: 48.00 ± 5.54<br>- Control group: 51.63 ± 7.49<br>Mean BMI (kg/m²):<br>- Intervention group: 24.07 ± 1.98<br>- Control group: 25.20 ± 2.94	Cardiorespiratory fitness, Muscular Strength	12 weeks	Exercise intervention (Home based → Combined Exercise)	12-week exercise intervention (→ Strength ↑, Endurance ↑, Overall fitness ↑)<br>→ Stand-up and Sit-down Chair Test (SPSDCT) ↑, Arm Lifting Test (ALT) ↑, VO_2 max ↑
85	Yee J., et al. (2019)	N = 14	Mean Age (years): 62.2 ± 10.6<br>Mean BMI (kg/m²): 28.3 ± 5.7	Cardiorespiratory fitness, Muscular Strength	8 weeks	Exercise intervention (Combined → Combined Exercise)	Partially supervised Physical activity program in women with metastatic breast cancer – Physical activity level, Physical fitness – Physical activity ↑, Overall fitness ↑
86	Hagstrom, A. D., et al. (2019)	N = 23	Mean Age (years): 50.39 ± 8.02	Muscular Strength	16 weeks	Exercise intervention (Supervised → Resistance Exercise)	Resistance training for breast cancer survivors (BCS) → Upper limb strength ↑, Electromyographic activity (isometric task): No change, Pectoral muscle median frequency (endurance task): ↓
87	Kim, M., Lee, et al. (2019)	N = 72	Mean Age (years):<br>- CSE group: 48.9 ± 7.2<br>- IBE group: 47.69 ± 8.9<br>Mean BMI (kg/m²):<br>- CSE group: 21.7 ± 21.5<br>- IBE group: 22.9 ± 22.9	Muscular Strength, Flexibility	12 weeks	Exercise intervention (Home based → Stretching, etc.)	12-week therapeutic inflatable ball self-exercise for breast cancer survivors (BCS) → Grip strength: No change
88	Leach, H. J., et al. (2019)	N = 26	Mean Age (years): 52 ± 8.5<br>Mean BMI (kg/m²): 29.3 ± 7.3	Cardiorespiratory fitness, Muscular Strength	8 weeks	Exercise intervention (Supervised → Combined Exercise)	8-week exercise interventions for breast cancer survivors (BCS) → Muscle strength ↑ (chest press, leg press, arm curl, sit-to-stand) in both groups (compared to baseline) No group difference
89	Okumatsu K., et al. (2019)	N = 32	Mean Age (years): 50 ± 6<br>Mean BMI (kg/m²):<br>- Intervention group: 24.3 ± 4.2<br>- Usual care group: 21.9 ± 3.8	Cardiorespiratory fitness	12 weeks	Exercise intervention (Supervised → Combined Exercise)	Combined exercise and diet program for Japanese breast cancer survivors (BCS) undergoing hormone therapy → Cardiorespiratory fitness ↑
90	Northey J. M., et al. (2019)	N = 17	Mean Age (years):<br>- CON group: 61.5 ± 7.8<br>- HIIT group: 60.3 ± 8.1<br>- MOD group: 67.8 ± 7.0	Cardiorespiratory fitness	12 weeks	Exercise intervention (Supervised → High-Intensity Interval Training, Steady-	High-intensity interval training (HIIT) for breast cancer survivors (BCS) → Maximal oxygen uptake (VO_2 peak) ↑

						state Continuous Exercise)	
91	McNeil J., et al. (2019)	N = 45	Mean Age (years):<br>- Control group: 60 ± 9<br>- Lower-intensity PA group: 58 ± 9<br>- Higher-intensity PA group: 58 ± 10<br>Mean BMI (kg/m²):<br>- Control group: 25.7 ± 3.5<br>- Lower-intensity PA group: 28.7 ± 4.9<br>- Higher-intensity PA group: 31.1 ± 8.4	Cardiorespiratory fitness	12 weeks	Exercise intervention (Home based → Steady-state Continuous Exercise)	12-week low- or high-intensity Physical activity for breast cancer survivors (BCS) → VO_2 max ↑ (regardless of intensity, at 12 and 24 weeks vs. baseline)
92	Ying, W., et al. (2019)	N=86	Mean Age (years): 54.1 ± 7.8<br>Mean BMI (kg/m²): 24.9	Flexibility	24 weeks	Exercise intervention (Combined→ etc.)	24-week Baduanjin exercise in breast cancer survivors → Shoulder ROM ↑, step test index (-)
93	Bail et al. (2018)	N = 82	Mean Age (years): 60.5 ± 9.4 Mean BMI (kg/m²): ≥25, 81.7%	Physical function	48 weeks	Non-exercise intervention (Vegetable gardening)	Intervention group: fitness performance ↑
94	Mijwel et al. (2018)	N = 84	Mean Age (years):<br>- SABC group: 55.1 ± 10.5<br>- PAL group: 56.0 ± 8.2<br><br>Mean BMI (kg/m²):<br>- SABC group: 26.4 ± 5.1<br>- PAL group: 29.2 ± 5.9	Muscular Strength	PAL (13weeks) SABC (4–8 weeks)	Exercise intervention (Combined → Resistance Exercise)	Strength After Breast Cancer (SABC) & Physical Activity and Lymphedema (PAL) → Muscular strength ↑, increase of muscular strength : PAL>SABC
95	Abdelhalim, N. M. and Samhan, A.F.(2018)	N = 43	Mean Age (years):<br>- ESWT group: 48.71 ± 3.07<br>- IPCT group: 49.55 ± 2.77<br>Mean BMI (kg/m²):<br>- ESWT group: 28.81 ± 1.78<br>- IPCT group: 29.36 ± 1.92	Muscular Strength	4 weeks	Non-exercise intervention (Extracorporeal shockwave therapy)	Extracorporeal shockwave therapy (ESWT) and intermittent pneumatic compression therapy (IPCT) → handgrip strength ↑
96	Travier, N., et al. (2018)	N = 37	Mean Age (years): 55.1 ± 8.3<br>Mean BMI (kg/m²): 30.5 ± 3.9	Cardiorespiratory fitness	12 weeks	Exercise intervention (Supervised → Combined Exercise)	After 12 weeks diet + exercise intervention (for overweight/obese BCS) → VO_2 peak ↑ 26.1% , Peak power output ↑ 32.4%.
97	Dieli-Conwright, C. M., et al. (2018)	N = 100	Mean Age (years): 53.5 ± 10.4<br>Mean BMI (kg/m²): 33.5 ± 5.5	Cardiorespiratory fitness, Muscular Strength	16 weeks	Exercise intervention (Supervised → Combined Exercise)	16-week aerobic + resistance exercise (for early survivors < 6 months post-treatment)<br>→ Physical fitness (all indicators) ↑ in the exercise group only

98	Dolan, L. B., et al. (2018)	N = 152	Mean Age (years): 54.45 ± 9.98<br>Mean BMI (kg/m²): 27.18 ± 6.59	Cardiorespiratory fitness	22 weeks	Exercise intervention (Combined → Combined Exercise)	22-week cardiac rehabilitation–based HEALTh program (home-based aerobic & resistance exercise)→ VO_2 peak ↑
99	Lahart, I. M., et al. (2018)	N = 32	Mean Age (years): 52.3 ± 9.6<br>Mean BMI (kg/m²): 27.2 ± 4.4	Cardiorespiratory fitness	24 weeks	Exercise intervention (Home based → Steady-state Continuous Exercise)	Exercise intervention for breast cancer survivors (BCS) → Maximal oxygen uptake (VO_2 max) ↑
100	Navarro-San A., et al. (2018)	N = 53	Mean Age (years):<br>- SPMA Pre-Test group: 51.4 ± 5.9<br>- Control Pre-Test group: 47.9 ± 7.8<br>Mean BMI (kg/m²):<br>- SPMA Pre-Test group: 27.9 ± 6.2<br>- Control Pre-Test group: 25.1 ± 4.7	Cardiorespiratory fitness, Muscular Strength, Flexibility	Not reported	Exercise intervention (Supervised → Combined Exercise)	Synchronized pedaling with martial arts program for breast cancer surgery patients (BCS) → Strength ↑, Aerobic capacity ↑
101	Luz, R. P. C., et al. (2018)	N=42	Mean Age (years): 59.5<br>BMI > 30: 55.6% (23/42)	Muscular Strength, Flexibility	8 weeks	Exercise intervention (Supervised → Combined Exercise)	8-week Strength Training with CPT in breast cancer surviviors → Shoulder ROM and strength ↑
102	Arikawa, A. Y. et al. (2018)	N=20	Mean Age (years): 56.6 ± 8.0<br>Mean BMI (kg/m²): 34.2 ± 5.5	Cardiorespiratory fitness	12 weeks	Exercise intervention (Combined→ Combined Exercise)	12-week Calorie restricted diet plus exercise in breast cancer survivors → Mets ↑
103	Desbiens, C., et al. (2017)	N = 26	Median Age (years):<br>- Group: 70.7 (IQR: 63.6–79.0)<br>- Individual: 67.5 (IQR: 60.7–72.7)<br><br>Median BMI (kg/m²):<br>- Group: 28.8 (IQR: 25.5–31.6)<br>- Individual: 25.6 (IQR: 22.8–27.2)	Cardiorespiratory fitness, Muscular Strength	12 weeks	Exercise intervention (Supervised, Home based → Combined Exercise)	After 12 weeks both groups (group and video) ↑ in all fitness measures ↑ / the body mass index (BMI) of group participants significantly decreased
104	Benton et al. (2017)	N = 17	Mean Age (years):<br>- RSM group: 60.6 ± 3.4<br>- LSM group: 55.5 ± 4.3<br>Mean BMI (kg/m²):<br>- RSM group: 27.2 ± 1.5<br>- LSM group: 28.4 ± 1.8	Muscular Strength, Flexibility	8 weeks	Exercise intervention (Supervised → Resistance Exercise)	After the 8week exercise intervention → RSM group: dominant (right) arm strength ↑ 25%, resolving initial imbalance.
105	Zengin Alpozgen, A., et al.(2017)	N = 57	Mean Age (years):<br>- PE group: 46.22 ± 11.19<br>- CE group: 51.94 ± 8.05<br>- HE group: 51.53 ± 13.81<br>Mean BMI (kg/m²):<br>- PE group: 30.68 ± 5.28	Muscular Strength, Flexibility	8 weeks	Exercise intervention (Supervised, Home based → Combined Exercise)	Pilates-based exercises (PE) and combined exercise (CE) group showed significant improvements in almost all the parameters ↑, and some improvements were superior to home exercise (HE) group

			- CE group: 28.73 ± 5.49<br>- HE group: 28.27 ± 3.99				
106	Storz, M. A., et al. (2017)	N = 40	Mean Age (years):<br>- Active Laser group: 61.06 ± 9.66<br>- Placebo Laser group: 59.37 ± 10.16	Muscular Strength	12 weeks	Non-exercise intervention (Photobiomodulation therapy)	12-week photobiomodulation therapy for lymphedema → Grip strength ↑ (both groups over time) ↔ (no significant difference between groups)
107	Sturgeon K. M., et al. (2017)	N = 35	Mean Age (years): 46.1 ± 4.0 (Total group)<br>Mean BMI (kg/m²):<br>- Control group: 29.6 ± 5.1<br>- Intervention group: 30.2 ± 4.6	Cardiorespiratory fitness, Muscular Strength	48 weeks	Exercise intervention (Supervised → Combined Exercise)	12-month web-based lifestyle program (Precision Nutrition Coaching) for BCS (Breast Cancer Survivors) → Cardiovascular health ↑ Lower & upper body muscle strength
108	Galiano-Castillo, N., et al. (2017)	N = 81	Mean Age (years): 48.30 ± 8.80	Cardiorespiratory fitness	8 weeks	Exercise intervention (Home based → Combined Exercise)	Remote rehabilitation for breast cancer survivors (BCS) → 6-minute walk test (6MWT) distance ↑, % predicted 6MWT ↑ (compared to baseline)
109	Schmidt T., et al. (2017)	N = 49	Mean Age (years):<br>- ACE group: 61.67 ± 10.01<br>- UC group: 53 ± 10.7<br>Mean BMI (kg/m²):<br>- ACE group: 30.42 ± 7.55<br>- UC group: 26.92 ± 6.42	Muscular Strength	12 weeks	Exercise intervention (Supervised → Resistance Exercise)	12-week arm ergometer training after axillary lymph node dissection for breast cancer survivors (BCS) → Upper limb strength ↑ (vs. control)
110	Kim, T. H., et al. (2017)	N = 30	Mean Age (years): 52.4 ± 6.5<br>Mean BMI (kg/m²): 24.5 ± 3.9	Cardiorespiratory fitness, Muscular Strength, Flexibility, Physical function	12 weeks	Exercise intervention (Supervised → Combined Exercise)	12-week exercise program for breast cancer survivors (BCS) → Grip strength ↑, Muscle endurance↑, Flexibility ↑
111	Leclerc, A. F., et al. (2017)	N = 209	Mean Age (years):<br>- Experimental group: 53.0 ± 8.9<br>- Control group: 53.7 ± 9.8<br>Mean BMI (kg/m²):<br>- Experimental group: 26.2 ± 4.6<br>- Control group: 25.7 ± 5.1	Cardiorespiratory fitness, Flexibility	12 weeks	Exercise intervention (Supervised → Combined Exercise)	12-week exercise intervention for breast cancer survivors (BCS) → Trunk flexibility ↑, Maximal oxygen uptake (VO_2 max) ↑, Maximal aerobic power ↑, Maximal heart rate ↑, 6-minute walk distance ↑
112	Madzima, T. A., et al. (2017)	N = 33	Mean Age (years):<br>- RT group: 59 ± 9<br>- RT+PRO group: 59 ± 7<br>Mean BMI (kg/m²):<br>- RT group: 27.2 ± 5.6<br>- RT+PRO group: 27.9 ± 6.0	Muscular Strength, Flexibility	12 weeks	Exercise intervention (Supervised → Resistance Exercise)	12-week exercise intervention for breast cancer survivors (BCS) → Grip strength ↑, Chest press 1RM ↑, Leg extension 1RM ↑ (in both groups)

113	De Groef, A., et al. (2017)	N=50	Mean Age (years): 54.2 ± 7.5<br>Mean BMI (kg/m²): 27.0 ± 4.4	Muscular Strength, Flexibility	12 weeks	Exercise intervention (Supervised→ Combined Exercise)	12-week combined therapy in breast cancer survivors → shoulder ROM ↑
114	Lee CE, Warden SJ, Szuck B, Lau YKJ (2016)	N = 56	Mean Age (years): 53.8 ± 9.6	Cardiorespiratory fitness, Muscular Strength, Physical function	6 weeks	Exercise intervention (Combined → Combined Exercise)	Overall improvement: all Physical function outcomes improved significantly over time ($p < 0.05$).
115	Buchan, J., et al. (2016)	N = 41	Mean Age (years): 56 (52.8–59.2)	Cardiorespiratory fitness, Muscular Strength	12 weeks	Exercise intervention (Combined → Combined Exercise)	The resistance-based exercise group → upper-body strength ↑ ($p < 0.05$),<br>Both (resistance, aerobic) groups → lower-body endurance and aerobic fitness ↑ but not statistically significant ($p > 0.05$)
116	Sheppard, V. B., et al. (2016)	N = 31	Mean Age (years):<br>- Intervention group: 54.7 ± 9.8<br>- Control group: not reported<br>Mean BMI (kg/m²):<br>- Intervention group: 35.2 ± 4.8<br>- Control group: 37.4 ± 8.6	Cardiorespiratory fitness	12 weeks	Exercise intervention (Supervised → Combined Exercise)	After 12weeks combined lifestyle intervention → improvement in cardiovascular health intervention group ↑ decrease in control group ↓
117	Dethlefsen, C., et al. (2016)	N = 94	Mean Age (years): 47.1 ± 8.8<br>Mean BMI (kg/m²):<br>- 6-month training group: 24.4 ± 3.7<br>- 2h acute exercise group: 26.0 ± 5.2	Cardiorespiratory fitness, Muscular Strength	24 weeks	Exercise intervention (Supervised → Combined Exercise)	6-month exercise vs. 2-hour acute exercise<br>→ VO_2 peak ↑ (6-month > acute)<br>6-month exercise (for BCS [Breast Cancer Survivors])<br>→ Physical fitness (all measures) ↑ in PA group
118	Dolan, L. B., et al. (2016)	N = 36	Mean Age (years): 57.2 ± 9<br>Mean BMI (kg/m²): 24.9 ± 4.7	Cardiorespiratory fitness, Muscular Strength	6 weeks	Exercise intervention (Supervised → Steady-state Continuous Exercise)	6-week supervised AIT (Aerobic Interval Training) or CMT (Continuous Moderate Training)<br>→ VO_2 peak ↑ (both groups, ~12%)<br>→ AIT group: Lower body strength ↑
119	Rogers L. Q., et al. (2016)	N = 222	Mean Age (years): 54 ± 9<br>Mean BMI (kg/m²):<br>- BEAT Cancer group: 30.8 ± 6.9<br>- Usual care group: 30.5 ± 7.0	Muscular Strength	6 weeks	Exercise intervention (Supervised → Combined Exercise)	Multicomponent Physical activity behavior change intervention for breast cancer survivors (BCS) → Back and leg muscle strength: No change
120	Ligibel, J. A., et al. (2016)	N = 101	Mean Age (years):<br>- Exercise group: 49.3 ± 9.6<br>- Control group: 50.7 ± 9.4	Cardiorespiratory fitness	16 weeks	Exercise intervention (Combined → Steady-state Continuous Exercise)	16-week exercise intervention for breast cancer survivors (BCS) → Cardiorespiratory fitness: No change (no group difference)
121	Taradaj, J., et. al. (2016)	N=70	Mean Age (years): 62.0 ± 4.7	Muscular Strength, Flexibility	4 weeks	Non-exercise intervention (Kinesiology tape application)	4-week kinesiology tapes apply in breast cancer survivors → Shoulder ROM and grip strength ↑, but most significant improve in arm volume, shoulder ROM and grip strengths in control (multilayered bandaging)

122	Brown, J. C., & Schmitz, K. H.(2015)	N = 295	Mean Age (years):<br>- Weight Lifting group: 55.3 ± 8.5<br>- Control group: 56.7 ± 9.1<br>Mean BMI (kg/m²):<br>- Weight Lifting group: 29 ± 5.9<br>- Control group: 28.7 ± 6.4	Muscular Strength	48 weeks	Exercise intervention (Combined → Combined Exercise)	After 12 months, the weight lifting group significantly increased their one-repetition maximum (1-RM) bench press and leg press strength compared to the control group ↑
123	Casla S. et al. (2015)	N = 94	Mean Age (years):<br>- EX group: 45.91 ± 8.21<br>- CON group: 51.87 ± 8.21<br>- Total group: 49.06 ± 8.75<br>Mean BMI (kg/m²):<br>- EX group: 25.97 ± 3.65<br>- CON group: 25.10 ± 5.85<br>- Total group: not reported (paired with Mean Age only)	Cardiorespiratory fitness, Muscular Strength, Flexibility	12 weeks	Exercise intervention (Supervised → Combined Exercise)	12-week intervention → exercise (EX) group: maximal oxygen uptake (VO_2 max ↑) (maintained at 6 months), isometric strength ↑, max strength ↑ (chest press, leg extension), muscular endurance ↑ vs. control (CON) ($p < 0.001$ or = 0.004)
124	Aweto, H. A., et al. (2015)	N = 54	Mean Age (years):<br>- Premenopausal A group: 39.43 ± 5.41<br>- Premenopausal B group: 39.75 ± 2.87<br>- Postmenopausal A group: 55.00 ± 6.29<br>- Postmenopausal B group: 51.00 ± 1.63<br>Mean BMI (kg/m²):<br>- Premenopausal A group: 29.66 ± 6.52<br>- Premenopausal B group: 29.98 ± 9.50<br>- Postmenopausal A group: 26.29 ± 6.10<br>- Postmenopausal B group: 33.28 ± 2.56	Cardiorespiratory fitness	12 weeks	Exercise intervention (Supervised → Combined Exercise)	Combined aerobic + stretching exercise (12 weeks) → maximal oxygen uptake (VO_2 max): premenopausal ↑, postmenopausal ↔
125	Brown, J. C., et al. (2015)	N = 295	Mean Age (years):<br>- Weight lifting group: 55.3 ± 8.5<br>- Control group: 56.7 ± 9.1	Muscular Strength	48 weeks	Exercise intervention (Combined → Resistance Exercise)	Slowly progressive weight lifting (12 months) → upper & lower body strength ↑ (bench press, leg press, handgrip)

126	Simonavice, E., et al. (2015)	N=27	Mean Age (years): 64 ± 7<br>Mean BMI (kg/m²): 27.7 ± 5.5	Muscular Strength, Flexibility	24 weeks	Exercise intervention (Supervised → Resistance Exercise)	6month resistance exercise intervention → Weight lifted (upper/lower body) ↑, Strength (upper/lower body) ↑
127	Swisher, A. K., et al. (2015)	N = 28	Mean Age (years):<br>- Intervention group: 53.8 (43–65)<br>- Control group: 53.6 (36–71)<br>Mean BMI (kg/m²):<br>- Intervention group: 30.9 ± 3.3<br>- Control group: 32.5 ± 7.1	Cardiorespiratory fitness	12 weeks	Exercise intervention (Combined → Steady-state Continuous Exercise)	12-week exercise (for TNBC [Triple-Negative Breast Cancer] survivors)<br>→ Exercise time ↑ VO_2 peak ↔
128	Vardar Yağlı, N., et al. (2015)	N = 52	Mean Age (years):<br>- Yoga + Aerobic group: 49.89 ± 4.65<br>- Aerobic group: 47.38 ± 7.57<br>Mean BMI (kg/m²):<br>- Yoga + Aerobic group: 29.16 ± 5.74<br>- Aerobic group: 29.27 ± 5.92	Cardiorespiratory fitness, Muscular Strength	6 weeks	Exercise intervention (Supervised → Combined Exercise, etc.)	Aerobic exercise and combined yoga + aerobic exercise in breast cancer survivors (BCS)<br>→ Functional capacity ↑, Muscle strength ↑
129	Vieira C. A., et al. (2015)	N = 30	Mean Age (years):<br>- BCS group: 52.0 ± 4.0<br>- CNT group: 53.0 ± 6.0<br>Mean BMI (kg/m²):<br>- BCS group: 30.6 ± 4.97<br>- CNT group: 30.1 ± 2.8	Muscular Strength	Not reported	Exercise intervention (Supervised → Resistance Exercise)	Isokinetic strength training in breast cancer survivors (BCS)<br>→ 2minute rest interval ↓ muscle fatigue more than 1-minute<br>→ BCS showed ↓ peak torque (PT) and total work (TW) vs. control<br>→ 2minute rest still insufficient → longer rest (>2 min) may be needed
130	Do J., et al. (2015)	N = 212	Mean Age (years):<br>- EEG group: 47.1 ± 8.5<br>- DEG group: 48.3 ± 8.2<br>Mean BMI (kg/m²):<br>- EEG group: ≥25: 17 (53.1%), <25: 15 (46.8%)<br>- DEG group: ≥25: 20 (66.6%), <25: 10 (33.3%)	Cardiorespiratory fitness, Muscular Strength, Flexibility	4 weeks	Exercise intervention (Supervised → Combined Exercise)	Supervised multimodal rehabilitation program→ Cardiopulmonary fitness ↑, Muscle strength
131	So H. J., et al. (2015)	N = 17	Mean Age (years):<br>- HR group: 48.1 ± 4.1<br>- LR group: 50.0 ± 4.1<br>Mean BMI (kg/m²):<br>- HR group: 26.2 ± 1.0<br>- LR group: 26.8 ± 1.6	Muscular Strength	8 weeks	Exercise intervention (Supervised → Resistance Exercise)	Elastic band exercise for breast cancer patients → Shoulder strength ↑, Elbow strength ↑, Upper arm lymphedema ↓<br>→ High-resistance group: Shoulder flexion ↑, Shoulder abduction ↑, Elbow flexion ↑, Elbow extension ↑, Upper arm edema ↓<br>→ Low-resistance group: Shoulder abduction ↑, Elbow flexion ↑
132	Hughes, D.C., et al. (2015)	N = 94	Mean Age (years): 56.2 ± 7.9<br>Mean BMI (kg/m²): 28.8 ± 6.7	Cardiorespiratory fitness, Muscular Strength,	24 weeks	Exercise intervention (Supervised →	Yoga exercise (YE) for breast cancer survivors (BCS) → Sit-to-Stand leg strength ↑ (compared to baseline and control)

				Flexibility, Physical function		Combined Exercise, etc.)	
133	Martin E. A., et al. (2015)	N = 72	Mean Age (years): 56.8 ± 9.6<br>Mean BMI (kg/m²):<br>- LIG group: 26.6 ± 4.8<br>- HIG group: 27.9 ± 5.3<br>- C group: 26.3 ± 5.2	Cardiorespiratory fitness	8 weeks	Exercise intervention (Supervised → Combined Exercise)	Low- and high-intensity exercise for breast cancer survivors (BCS) → VO_2 peak ↑ in both (vs. control) maintained only in high-intensity group (at 4 months)
134	Fischer, M. J., et al. (2015)	N=28	Mean Age (years): 53.8 ± 10.0	Flexibility	10 weeks	Exercise intervention (Supervised → Steady-state Continuous Exercise)	10-week Nordic Walking intervention in breast cancer survivors → shoulder ROM ↑
135	Rogers, L. Q., et al. (2015)	N=222	Mean Age (years): 54.4 ± 8.5	Cardiorespiratory fitness	12 weeks	Exercise intervention (combined→ Steady-state Continuous Exercise)	12-week aerobic exercise intervention in breast cancer survivors → aerobic fitness (vo2 max) ↑
136	Atalay, O. T., et al. (2015)	N=58	Mean Age (years): 43.5 ± 5.3<br>Mean BMI (kg/m²): 26.3 ± 1.2	Flexibility	4 weeks	Non-exercise intervention (Complex decongestive physiotherapy)	4-week complex decongestive physiotherapy (CDP) in breast cancer survivors → Shoulder ROM ↑
137	K. Johansson, P. Klernäs, A. Weibull, S. Mattsson (2014)	N = 23	Mean Age (years): 58 ± 8<br>Mean BMI (kg/m²): 25.8 ± 3.2	Muscular Strength	12 weeks	Exercise intervention (Home based → Resistance Exercise)	12-week self-administered weight lifting exercise (EX) → significant increase in muscle strength ↑
138	Cerulli, C., et al. (2014)	N = 20	Mean Age (years):<br>- Intervention group: 45.61 ± 2.71<br>- Control group: not reported<br>Mean BMI (kg/m²):<br>- Intervention group: 23.51 ± 2.89<br>- Control group: 25.81 ± 4.82	Cardiorespiratory fitness, Muscular Strength, Flexibility	16 weeks	Non-exercise intervention (Equine-assisted therapy)	16-week equine-assisted therapy (EAT) protocol significantly improved maximal oxygen uptake (VO_2 max), body composition, and muscle strength in the intervention group ↑
139	Dobek, J., et al. (2014)	N = 67	Mean Age (years):<br>- POWIR group: 64.2 ± 6.0<br>- FLEX group: 63.8 ± 7.3<br>Mean BMI (kg/m²):<br>- POWIR group: 28.9 ± 5.5<br>- FLEX group: 27.9 ± 4.5	Muscular Strength	48 weeks	Exercise intervention (Combined → Stretching, Resistance Exercise)	After 1 year, the POWIR group maintained spine bone mineral density (BMD) (↔) but exhibited a decline in muscle strength (↓), while the FLEX group experienced bone loss (↓) with minimal change in muscle strength (↔).

140	Rogers L. Q., et al. (2014)	N = 46	Mean Age (years): 56.2 ± 7.7 (32–69)	Cardiorespiratory fitness, Muscular Strength	12 weeks	Exercise intervention (Combined → Combined Exercise)	Exercise program → cardiorespiratory fitness ↑ back and leg muscle strength: no significant between-group difference
141	Simonavice E., et al. (2014)	N = 27	Mean Age (years):<br>- RT group: 64 ± 5<br>- RT+DP group: 64 ± 7<br>Mean BMI (kg/m²):<br>- RT group: 26.8 ± 5.0<br>- RT+DP group: 26.9 ± 3.7	Muscular Strength, Flexibility	24 weeks	Exercise intervention (Supervised → Resistance Exercise)	Resistance training (RT) and RT + dried plum (DP) intervention → Upper and lower body strength ↑
142	Travier N., et al. (2014)	N = 42	Mean Age (years): 54.8 ± 8.7<br>Mean BMI (kg/m²): 30.4 ± 3.8	Cardiorespiratory fitness	12 weeks	Exercise intervention (Supervised → Combined Exercise)	Short-term diet + PA intervention→ Cardiofitness ↑ VO_2 peak ↑ 26.7%, Peak power output ↑ 34.3%
143	Fisher, M. I., et, al. (2014)	N = 6	Mean Age (years): 57 ± 7<br>Mean BMI (kg/m²): 27.8 ± 5.6	Muscular Strength	8 weeks	Exercise intervention (Combined → etc.)	8-week yoga intervention for breast cancer survivors (BCS) → Grip strength: No change
144	Hanuszkiewicz, J., et al. (2014).	N = 60	Mean Age (years):<br>- NE group: 57.3 ± 8.05<br>- GE group: 59.4 ± 7.47<br>- WE group: 63.0 ± 7.58	Muscular Strength, Flexibility	8 weeks	Exercise intervention (Supervised → Steady-state Continuous Exercise, Combined Exercise)	Nordic walking and water resistance exercise for breast cancer survivors (BCS) → Trunk muscle strength ↑, Trunk muscle endurance ↑
145	Repka C. P., et al. (2014)	N = 319	Mean Age (years):<br>- Breast group: 55.8 ± 10.0<br>- Prostate group: 68.1 ± 10.6<br>- Hematological group: 55.1 ± 15.6<br>- Colon group: 60.3 ± 13.3<br>- Gynecological group: 55.1 ± 9.9<br>- GEN group: 61.4 ± 13.8<br>- Lung group: 61.3 ± 14.3<br><br>Mean BMI (kg/m²):<br>- Breast group: 28.3 ± 6.3<br>- Prostate group: 29.8 ± 5.6<br>- Hematological group: 30.8 ± 5.5<br>- Colon group: 30.6 ± 5.8<br>- Gynecological group: 30.3 ± 6.0<br>- GEN group: 30.2 ± 6.5<br>- Lung group: 30.0 ± 6.7	Cardiorespiratory fitness	12 weeks	Exercise intervention (Supervised → Combined Exercise)	3-month rehabilitation exercise for breast cancer survivors (BCS) → Peak oxygen uptake (VO_2 peak) ↑
146	Murtezani A., et al. (2014)	N = 73	Mean Age (years): 52 ± 11<br>Mean BMI (kg/m²): 26.0 ± 3.1	Cardiorespiratory fitness	10 weeks	Exercise intervention (Supervised →	10-week moderate-intensity aerobic exercise for breast cancer survivors (BCS) → 12-minute walk distance ↑

						Steady-state Continuous Exercise)	
147	Sato, F., et al. (2014)	N=149	Mean Age (years): 53.3 ± 10.8	Muscular Strength, Flexibility	Not reported	Non-exercise intervention (Coaching based on the UCSF Symptom Management Model)	Before and after preoperative education → grip strength ↑ bit ROM -
148	Cantarero-Villanueva, I., et al. (2013)	N = 68	Mean Age (years):<br>- Aquatic Exercise group: 49 ± 7<br>- Usual care group: 47 ± 8	Muscular Strength	8 weeks	Exercise intervention (Supervised → Combined Exercise)	8-week deep water aqua exercise → lower body & abdominal strength ↑, multiple standing test time ↓ , trunk curl endurance ↑
149	Cormie, P., et al. (2013)	N = 62	Mean Age (years):<br>- High-load resistance group: 56.1 ± 8.1<br>- Low-load resistance group: 57.0 ± 10.0<br>- Control group: 58.6 ± 6.7<br>Mean BMI (kg/m²):<br>- High-load resistance group: 30.8 ± 6.5<br>- Low-load resistance group: 30.4 ± 5.7<br>- Control group: 28.2 ± 6.0	Muscular Strength	12 weeks	Exercise intervention (Supervised → Resistance Exercise)	3-month supervised resistance training (for women with lymphedema)<br>→ Physical function ↑ (very effective)
150	Gallant, N. R., et al. (2013)	N = 139	Mean Age (years): 60.9 (41–86)	Physical function	12 weeks	Exercise intervention (Supervised → Combined Exercise)	Strong Women: New STEPS program for breast cancer survivors (BCS) → Strength ↑, Flexibility ↑, Balance ↑ (STEPS: Strength Through Education, Physical fitness and Support)
151	Kim, Y. S et al. (2013)	N = 35	Mean Age (years): 56.03 ± 7.96	Muscular Strength	12 weeks	Exercise intervention (Supervised → etc.)	Rehabilitation Qigong exercise for breast cancer survivors (BCS) → Grip strength ↑
152	Rogers L. Q., et al. (2013)	N = 28	Mean Age (years): 56 ± 10.5<br>Mean BMI (kg/m²):<br>- Intervention group: 33.9 ± 7.4<br>- Usual care group: 30.3 ± 7.11	Cardiorespiratory fitness, Muscular Strength	12 weeks	Exercise intervention (Combined → Resistance Exercise)	Physical activity behavior change interventions for breast cancer survivors (BCS) → Cardiorespiratory fitness ↑, Muscle strength ↑
153	Martin, E., et al. (2013)	N = 26	Mean Age (years):<br>- MFC group: 44.6<br>- TRT group: 47.8<br>- Control group: 49.5	Muscular Strength	8 weeks	Exercise intervention (Supervised → Resistance Exercise)	8-week Pilates-based MVe Fitness Chair™ program for breast cancer survivors (BCS) → Muscular endurance ↑
154	Loudon, A., Barnett, T., et al. (2013)	N = 28	Mean Age (years): 57.6 ± 10.5<br>Mean BMI (kg/m²): 27.2 ± 4.9	Muscular Strength, Flexibility	8 ~ 12 weeks	Exercise intervention (Combined → etc.)	Yoga intervention for breast cancer survivors (BCS) → Serratus anterior strength ↑, Pectoralis major strength ↓ (8–12 weeks)

155	Winters-Stone, K. M., et al. (2012)	N = 106	Mean Age (years):<br>- POWIR group: 62.3 ± 6.7<br>- FLEX group: 62.2 ± 6.7<br>Mean BMI (kg/m²):<br>- POWIR group: 29.5 ± 5.8<br>- FLEX group: 29.5 ± 5.6	Muscular Strength, Physical function	16 weeks	Exercise intervention (Combined → Resistance Exercise, Stretching)	Resistance + impact training → upper and lower body strength ↑ balance and Physical function: no significant change
156	Nikander, R., et al. (2012)	N = 86	Mean Age (years):<br>- Training group: 53.7 ± 6.8<br>- Control group: 52.6 ± 7.1	Cardiorespiratory fitness, Muscular Strength, Physical function	48 weeks	Exercise intervention (Combined → Steady-state Continuous Exercise)	12-month vigorous aerobic exercise → figure-8 running time (agility) ↑, grip strength ↑
157	Brdareski Z. et al. (2012)	N=18	Mean Age (years):<br>- E1 group: 51.60 ± 7.47<br>- E2 group: 52.75 ± 7.42<br>Mean BMI (kg/m²):<br>- E1 group: 26.73 ± 2.11<br>- E2 group: 26.19 ± 4.87	Cardiorespiratory fitness	3 weeks	Exercise intervention (Supervised → Steady-state Continuous Exercise)	3-week moderate-intensity aerobic exercise → maximal oxygen uptake (VO_2 max) ↑, cardiorespiratory fitness ↑
158	Saarto T., et al. (2012)	N = 573	Mean Age (years):<br>- Exercise group: 52.3 (36–68)<br>- Control group: 52.4 (35–68)	Cardiorespiratory fitness, Physical function	48 weeks	Exercise intervention (Combined → Combined Exercise)	Exercise training for breast cancer survivors (BCS) → Physical function: No change, Physical activity: No change
159	Naumann F., et al. (2012)	N = 46	Mean Age (years):<br>- Counseling group: 55.1 ± 7.5<br>- Exercise group: 49.0 ± 10.0<br>- Exercise & Counseling group: 49.0 ± 8.2<br>- Usual care group: 51.8 ± 11.5<br>Mean BMI (kg/m²):<br>- Counseling group: 24.9 ± 1.68<br>- Exercise group: 27.3 ± 1.50<br>- Exercise & Counseling group: 27.5 ± 1.49<br>- Usual care group: 27.4 ± 1.59	Cardiorespiratory fitness, Muscular Strength	8 weeks	Exercise intervention (Supervised → Combined Exercise)	Combined exercise and psychological counseling program for breast cancer survivors (BCS) → Upper-body strength ↑, Lower-body dynamic strength ↑, Cardiorespiratory endurance ↑
160	Musanti R., et al. (2012)	N = 42	Mean Age (years): 50.5 ± 7.5	Cardiorespiratory fitness, Muscular Strength	12 weeks	Exercise intervention (Home based → Resistance Exercise, Combined Exercise, Steady-state Continuous Exercise)	12-week home-based exercise for breast cancer survivors (BCS) → Resistance group (R): Upper extremity strength ↑, Aerobic group (A): Cardiorespiratory fitness ↑, Aerobic + Resistance group (AR): No change

161	Tunay, V. B., et al. (2012)	N=40	Mean Age (years): older than 65 years	Flexibility	12 weeks	Non-exercise intervention (Manual Therapy)	12-week combined therapy in breast cancer survivors → Shoulder ROM ↑
162	Ahmed Omar et al. (2011)	N = 58	Mean Age (years):<br>- Active laser group: 54.76 ± 3.33<br>- Placebo laser group: 53.36 ± 3.56<br>Mean BMI (kg/m²):<br>- Active laser group: 29.1 ± 6.6<br>- Placebo laser group: 25.6 ± 3.3	Muscular Strength	12 weeks	Exercise intervention (Home based → Stretching)	Active laser therapy → grip strength compared to placebo ($p < 0.01$).<br>Active laser therapy group: 38.85% improvement in grip strength at 12 weeks ($p < 0.01$) ↑.<br>Placebo laser group: 16.59% improvement in grip strength at 12 weeks ↑.
163	Bower, J. E., et al. (2011)	N = 12	Mean Age (years): 53.8 (46–65)	Muscular Strength, Physical function	12 weeks	Exercise intervention (Supervised → etc.)	12-week Iyengar yoga program → chair stand Repetitions increased ↑, 8-foot walk test ↔
164	Van Puymbroeck, M., et al. (2011)	N = 44	Mean Age (years): 56.67 ± 10.47	Muscular Strength, Flexibility, Physical function	8 weeks	Exercise intervention (Supervised → etc.)	Hatha yoga for breast cancer survivors (BCS) → Physical activity constraints ↓, Fitness (strength and flexibility) ↑
165	Kaltsatou, A., et al. (2011)	N = 27	Mean Age (years):<br>- Experimental group: 56.6 ± 4.2<br>- Control group: 57.1 ± 4.1	Cardiorespiratory fitness, Muscular Strength	24 weeks	Exercise intervention (Supervised → Combined Exercise)	Exercise intervention for breast cancer survivors (BCS) → Grip strength ↑, Walking distance ↑ (vs. control)
166	Mehnert A, et al. (2011)	N = 58	Mean Age (years): 51.88 ± 8.46	Cardiorespiratory fitness	10 weeks	Exercise intervention (Supervised → Combined Exercise)	10-week Physical exercise after treatment for breast cancer survivors (BCS) → VO_2 max↑
167	Speck, R. M., et al. (2010)	N = 295	Mean Age (years):<br>- Treatment group (with lymphedema): 56 ± 9<br>- Control group (with lymphedema): 58 ± 9<br>- Treatment group (without lymphedema): 55 ± 7<br>- Control group (without lymphedema): 57 ± 8	Muscular Strength	12 weeks	Exercise intervention (Combined → Combined Exercise)	After 1years strength training → Bench press ↑, Leg press ↑ (treatment vs. control)
168	Sprod L. K., et al. (2010)	N = 114	Mean Age (years):<br>- Sedentary group: 61.2 ± 4.0<br>- 3M group: 60.3 ± 2.4<br>- 6M group: 57.6 ± 1.2	Cardiorespiratory fitness, Muscular Strength	12 weeks vs 24 weeks	Exercise intervention (Supervised → Combined Exercise)	3- & 6-month individualized exercise → Cardiovascular endurance ↑ Muscular endurance ↑ (only in 6-month)
169	Eyigor, S., et al. (2010)	N = 52	Mean Age (years):<br>- Group 1: 48.52 ± 7.62<br>- Group 2: 49.73 ± 8.71	Cardiorespiratory fitness, Flexibility	8 weeks	Exercise intervention (Combined → etc.)	8-week Pilates intervention→ 6MWT (6-Minute Walk Test) ↑ Flexibility ↑ (not statistically significant)

170	YW, Kim , et al. (2010)	N = 21	Mean Age (years):<br>- MRM group: 54.3 ± 7.39<br>- BCS group: 46.82 ± 10.21	Muscular Strength, Flexibility	12 weeks	Exercise intervention (Supervised → Combined Exercise)	12week exercise intervention for modified radical mastectomy (MRM) breast cancer survivors (BCS)<br>→ Grip strength ↑, Sit-and-reach flexibility ↑<br><br>12-week exercise intervention for breast cancer survivors (BCS)<br>→ Sit-and-reach flexibility ↑
171	Lee, S. A., et al. (2010)	N = 44	Mean Age (years):<br>- Scapula-oriented group: 47.5 ± 5.1<br>- General group: 45.6 ± 7.0<br>- Control group: 47.6 ± 9.2	Muscular Strength, Flexibility	8 weeks	Exercise intervention (Supervised → Combined Exercise)	8-week scapula-oriented exercise for breast cancer survivors (BCS) → Shoulder external rotation strength ↑ (vs. general exercise and control group)
172	Ryu and Kim (2010)	N=11	Mean Age (years): 53.6 ± 9.8	Cardiorespiratory fitness, Muscular Strength, Flexibility	15 weeks	Exercise intervention (Supervised → Combined Exercise)	15-week chair exercise in breast cancer survivors → sit and reach ↑
173	YW, Kim et al. (2009)	N = 40	Mean Age (years):<br>- CEG group: 47.68 ± 3.40<br>- SEG group: 51.40 ± 5.04<br>- NEG group: 48.47 ± 5.03	Muscular Strength, Flexibility, Physical function	12 weeks	Exercise intervention (Supervised → Combined Exercise)	12-week yoga, elastic band, and Swiss ball exercise program for breast cancer survivors (BCS)<br>→ Left-hand grip strength ↑, Balance ↑, Sit and Reach ↑
174	Won, H. J., et al. (2009)	N = 34	Age Distribution (%):<br>- Experimental group:<br><49 years: 58%, 50–59 years: 32%, ≥60 years:11%<br><br>- Control group:<br><49 years: 33%, 50–59 years: 53%, ≥60 years: 13%	Muscular Strength	8 weeks	Exercise intervention (Combined → Resistance Exercise)	Breast cancer patients after surgery who participated in the cancer-overcome BeHaS exercise program → No significant difference in muscle strength
175	Hokken, J.W.E., et al. (2009)	N = 75	Mean Age (years):<br>- AC group: 49.0 ± 7.8<br>- FEC group: 49.7 ± 8.3<br>Mean BMI (kg/m²):<br>- AC group: 26.4 ± 5.7<br>- FEC group: 25.7 ± 4.9	Cardiorespiratory fitness, Muscular Strength	18 weeks	Exercise intervention (Supervised → Combined Exercise)	18-week exercise intervention for breast cancer survivors (BCS) → Muscle function ↑, Cardiopulmonary function (VO_2 max) ↑ (in both groups)
176	Schmitz K. H., et al. (2009)	N = 141	Mean Age (years):<br>- Weight lifting group: 56 ± 9<br>- Control group: 58 ± 10<br>Mean BMI (kg/m²):<br>- Weight lifting group: 31.0 ± 6.2<br>- Control group: 29.9 ± 6.6	Muscular Strength	48 weeks	Exercise intervention (Combined → Resistance Exercise)	Slowly progressive weight-lifting for breast cancer survivors (BCS) with lymphedema program→ Bench press ↑, Leg press ↑, Maximal strength ↑

177	Kozanoglu, E., et al. (2009)	N = 47	Mean Age (years): 48.3 ± 10.4	Muscular Strength, Flexibility	4 weeks	Non-exercise intervention (Pneumatic compression therapy)	Exercise intervention for breast cancer survivors (BCS) → Grip strength ↑ (no group difference)
178	City C. Hsieh et al. (2008)	N = 96	Mean Age (years):<br>- Surgery alone group: 55.6 ± 11.3<br>- Surgery + Chemo group: 55.6 ± 11.0<br>- Surgery + Radiation group: 57.2 ± 19.4<br>- Surgery + Chemo + Radiation group: 63.1 ± 19.8	Cardiorespiratory fitness	24 weeks	Exercise intervention (Supervised → Combined Exercise)	The exercise intervention → ↑cardiopulmonary function, ↑ peak oxygen uptake (pVO_2 max), ↑ treadmill time, ↓ resting heart rate, ↑ forced vital capacity (FVC)
179	Hughes, D. C., et al. (2008)	N = 25	Mean Age (years): 50 ± 8.44	Cardiorespiratory fitness, Muscular Strength, Flexibility	10 weeks	Exercise intervention (Home based → Combined Exercise)	10-week home-based exercise program led to improvements in peak oxygen uptake (VO_2 peak) ↑, arm strength ↑↑, and flexibility ↑↑.
180	Portela, A. L. M., et al. (2008)	N = 44	Mean Age (years):<br>- Non-exercise group: 59.6 ± 16.7<br>- Gym-exercise group: 49.8 ± 6.9<br>- Home-exercise group: 51.2 ± 7.3	Cardiorespiratory fitness, Muscular Strength, Flexibility	26 weeks	Exercise intervention (Supervised, Home based → Combined Exercise)	26-week gym or home-based exercise intervention for breast cancer survivors (BCS) → 12-minute walk test ↑ (significant only in home group), Shoulder external rotation ↑ (gym & home vs. control), Grip strength: No change
181	Milne H. M., et al. (2008)	N = 58	Mean Age (years): 55.1 ± 8.2<br>Mean BMI (kg/m^2): 26.3 ± 4.6	Cardiorespiratory fitness, Muscular Strength	12 weeks	Exercise intervention (Supervised → Combined Exercise)	Supervised aerobic + resistance exercise 3×/week for breast cancer survivors (BCS) → Fitness ↑, Muscle strength ↑ in both immediate and delayed groups
182	Nikander R., et al. (2007)	N = 28	Mean Age (years): 52.5 ± 6.4<br>Mean BMI (kg/m^2):<br>- Training group: 27.4 ± 5.4 → 26.9 ± 5.6<br>- Control group: 27.6 ± 4.6 → 27.7 ± 4.9	Cardiorespiratory fitness, Muscular Strength, Physical function	12 weeks	Exercise intervention (Combined → Circuit Training, Steady-state Continuous Exercise)	12-week aerobic exercise → training group: Physical performance ↑
183	Herrero, F., et al. (2007)	N = 11	Mean Age (years): 47 ± 7	Cardiorespiratory fitness, Muscular Strength, Flexibility	8 weeks	Exercise intervention (Supervised → Combined Exercise)	8-week exercise meditation for breast cancer survivors (BCS) → VO_2 peak ↑, Sit-to-Stand Test speed ↑, Cardiopulmonary strain after exercise ↓, Muscular endurance: No change
184	Cheema, B. S. B., & Gaul, C. A. (2006)	N = 31	Mean Age (years): 57.7 ± 7.7	Cardiorespiratory fitness, Muscular Strength, Flexibility	8 weeks	Exercise intervention (Combined → Combined Exercise)	8-week resistance + aerobic exercise → strength and endurance ↑, peak oxygen uptake (VO_2 peak) ↑

185	Damush, T. M., et al. (2006)	N = 34	Mean Age (years):<br>- Intervention group: 53.4 ± 10.0<br>- Control group: 56.6 ± 10.0<br>Mean BMI (kg/m²):<br>- Intervention group: 24.8 ± 3.6<br>- Control group: 28.1 ± 5.0	Flexibility, Physical function	10 weeks	Exercise intervention (Combined → Combined Exercise)	6-month exercise intervention (for older cancer survivors) →health ↑ Strength ↑ Endurance ↑
186	Herrero, F., et al. (2006)	N = 20	Mean Age (years):<br>- Either training group: 50 ± 5<br>- Control group: 51 ± 10<br>Mean BMI (kg/m²):<br>- Either training group: 24.0 ± 3.2<br>- Control group: 25.1 ± 3.5	Cardiorespiratory fitness, Muscular Strength, Flexibility	8 weeks	Exercise intervention (Supervised → Combined Exercise)	8-week training intervention for breast cancer survivors (BCS) → VO_2 peak ↑, Peak power output (PPO) ↑, Peak ventilation ↑, Leg press ↑, Sit-to-Stand Test ↑ (Bench press, ventilatory equivalents, respiratory exchange ratio: No change)
187	Rabin C. S., et al. (2006)	N = 83	Mean Age (years):<br>- MF group: 53.42 ± 9.10<br>- OTM group: 54.60 ± 9.10	Cardiorespiratory fitness	12 weeks	Exercise intervention (Home based → Steady-state Continuous Exercise)	Moving Forward (MF) intervention for breast cancer survivors (BCS) → Walk time ↓
188	Lane, K., Jespersen, D., & McKenzie, D. C.(2005)	N = 16	Mean Age (years): 52.4 ± 6.8<br>Mean BMI (kg/m²): 24.1 ± 2.8	Muscular Strength	20 weeks	Exercise intervention (Supervised, Home based → Combined Exercise)	A significant increase in bench press 1-repetition maximum (1-RM): increased upper body strength ↑
189	Morrell, R. M., et al. (2005)	N = 25	Mean Age (years):<br>- ALT group: 56.4 ± 9.76<br>- Control group: 53.4 ± 9.35<br>Mean BMI (kg/m²):<br>- ALT group: 25.7 ± 3.2<br>- Control group: 26 ± 4	Muscular Strength	12 weeks	Exercise intervention (Supervised, Combined → etc.)	Aqua Lymphatic Therapy (ALT) intervention → Compared with baseline both groups: handgrip strength ↑ in both hands
190	Pinto, B. M., et al. (2005)	N = 86	Mean Age (years):<br>- PA Intervention group: 53.42 ± 9.08<br>- Control group: 52.86 ± 10.38<br>Mean BMI (kg/m²):<br>- PA Intervention group: 27.51 ± 5.04<br>- Control group: 28.56 ± 5.50	Cardiorespiratory fitness	12 weeks	Exercise intervention (Home based → Steady-state Continuous Exercise)	12-week Physical activity (PA) motivate intervention → Aerobic fitness ↑
191	Joo, Ok-Hee. (2004)	N = 55	Mean Age (years): 49.07 ± 7.58	Muscular Strength	10 weeks	Exercise intervention (Combined → etc.)	10-week comprehensive rehabilitation program for breast cancer survivors → Hand grip strength ↑
192	Courneya, K. S., et al. (2003)	N = 53	Mean Age (years): 59 ± 6<br>Mean BMI (kg/m²): 29.2 ± 6.6	Cardiorespiratory fitness	15 weeks	Exercise intervention (Supervised → Steady-state Continuous Exercise)	After 15 weeks → exercise group: ↑ maximal oxygen uptake (VO_2 max), ↑ peak power control group: ↓ or ↔

193	Waltman N. L., et al. (2003)	N = 21	Mean Age (years): 54.4 ± 7.2	Muscular Strength, Physical function	48 weeks	Exercise intervention (Combined → Resistance Exercise)	Personal trainer-led exercise in breast cancer survivors (BCS) → Endurance ↑ (2-minute step test), Muscle strength ↑ (bicep curls)<br>→ Flexibility (back scratch test) ↔
194	Tiina, T. (1996)	N = 15	Median Age (years): 56 (Range: 39–72)	Muscular Strength	4 weeks	Non-exercise intervention (Pharmacological intervention (neuropathic pain relief))	Grip strength in the ipsilateral hand ↓ compared to the contralateral hand before treatment neither amitriptyline nor placebo affected grip strength.
195	Lee, S. H., et al (2020)	N = 24	Mean Age (years):<br>- EXE group: 52.5 ± 4.55<br>- CON group: 51.7 ± 4.03<br>Mean BMI (kg/m²):<br>- EXE group: 23.98 ± 2.32<br>- CON group: 24.15 ± 2.69	Cardiorespiratory fitness, Muscular Strength, Flexibility	12 weeks	Exercise intervention (Supervised → etc.)	12-week Taekwondo training for breast cancer survivors (BCS) → Grip strength ↑, Flexibility ↑
196	Park S. Y., et al (2013)	N = 61	Mean Age (years):<br>- Experimental group: 50<br>- Control group: 49	Muscular Strength, Flexibility	10 weeks	Exercise intervention (Combined → Combined Exercise)	10-week BEHAS exercise program for breast cancer survivors (BCS) → Grip strength ↑ (both arms, vs. control), Flexibility ↓ (unaffected arm, vs. control)
197	Kim, M. S.(2010)	N = 35	Mean Age (years):<br>- 3WEG group: 47.7 ± 3.50<br>- 1WEG group: 52.5 ± 8.96<br>- NEG group: 48.2 ± 5.78<br>Mean BMI (kg/m²):<br>- 3WEG group: 23.41 ± 2.25<br>- 1WEG group: 21.98 ± 2.24<br>- NEG group: 24.36 ± 2.23	Muscular Strength, Flexibility	12 weeks	Exercise intervention (Supervised → Combined Exercise)	12-week strap exercise for breast cancer survivors (BCS):<br>→ Flexibility (sit and reach) ↑ in 3WEG (3 times/week) and 1WEG (1 time/week) (3WEG > 1WEG)<br>→ Grip strength: No change in all groups
198	Lee, C. M., et al. (2008)	N = 20	Mean Age (years):<br>- EG group: 47.90 ± 4.51<br>- CG group: 52.90 ± 7.50	Cardiorespiratory fitness, Muscular Strength, Flexibility	12 weeks	Exercise intervention (Supervised → Combined Exercise)	12-week combined exercise program for breast cancer survivors (BCS) → Physical efficacy index ↑, Flexibility ↑, Muscular endurance ↑
199	Kim, M. S. (2004)	N = 10	Mean Age (years): 48.00 ± 3.65	Muscular Strength, Flexibility, Physical function	10 weeks	Exercise intervention (Supervised → etc.)	10-week swimming program for breast cancer survivors (BCS) → Grip strength ↑, Flexibility ↑, Agility ↑, Muscle endurance↑ (compared to baseline)

↑ (increased or improved); ↓ (decreased or deteriorated).

Table S7. Studies Categorized by Phase: Unknown (N = 2).

No.	Author (year)	Sample size	Participant Characteristics	Type of fitness outcome	Intervention duration	Intervention type	Key Results
1	Bilek, F., Deniz, G., & Gulkesen, A (2023)	N = 21	Mean Age (years): 52.21 ± 7.20<br>Mean BMI (kg/m²): 30.04 ± 2.72	Physical function	6 weeks	Non-exercise intervention (Complex decongestive therapy)	Complex decongestive therapy (CDT) → gait & balance ↑, Timed Up and Go test & plantar pressure ↓ in breast cancer-related lymphedema
2	Pedro. A et al. (2019)	N = 19	Mean Age (years):<br>- Group A: 52.3 ± 10.9<br>- Group B: 51.5 ± 2.9<br>Mean BMI (kg/m²):<br>- Group A: 28.1 ± 2.2<br>- Group B: 26.3 ± 2.3	Cardiorespiratory fitness, Muscular Strength	16 weeks	Exercise intervention (Supervised → Combined Exercise)	After 16 weeks of exercise cessation the group A (GA, patients 14–30 months post breast cancer diagnosis) showed improvements in all physical fitness measures compared to the group B (GB, patients 74–92 months post breast cancer diagnosis) ↑

↑ indicates increased or improved; ↓ indicates decreased or deteriorated; ↔ indicates no meaningful or statistically significant change.

Table S8. Studies Categorized by Phase: Mixed Phases (N = 9).

No.	Author (year)	Sample size	Treatment Phases	Participant Characteristics	Type of fitness outcome	Intervention duration	Intervention type	Key Results
1	Carpenter, D. J., et al. (2024)	N = 43	during treatment phase. post-treatment phase	Median Age (years): 56.5	Muscular Strength, Physical function	12 weeks	Exercise intervention (Supervised → Resistance Exercise)	3-month exercise program → muscle mass & grip strength ↑, balance & functional movement
2	Kendall, S. J., et, al. (2023)	N = 43	pre-treatment phase, during treatment phase, post-treatment phase	Mean Age (years): 57.3 ± 10.1<br>Mean BMI (kg/m²): 27.4 ± 5.1	Cardiorespiratory fitness, Muscular Strength, Flexibility, Physical function	12 weeks	Exercise intervention (Supervised → Combined Exercise)	12-week exercise intervention for breast cancer survivors (BCS) → 6-minute walk test (6MWT) ↑, 30-second chair sit-to-stand test ↑ (compared to baseline)
3	Schulz S. V. W., et al. (2022)	N = 26	post-treatment phase, unknown	Mean Age (years):<br>- Intervention group: 58.7 ± 8.4<br>- Control group: 58.8 ± 6.6	Cardiorespiratory fitness, Muscular Strength	6 weeks	Exercise intervention (Supervised → Resistance Exercise, High-Intensity Interval Training)	6-week high-intensity interval training (HIIT) and high-intensity resistance training (HIRT) for breast cancer survivors (BCS) → Maximal oxygen uptake (VO_2 max) ↑, One-repetition maximum (1RM) ↑
4	Bell K. E, et al. (2021)	N = 52	during treatment phase, post-treatment phase	Mean Age (years): 53 ± 10<br>Mean BMI (kg/m²): 27.5 ± 5.4	Cardiorespiratory fitness, Muscular Strength	12 weeks	Exercise intervention (Supervised → Combined Exercise)	Low-frequency exercise training (1, 2 sessions/week) → Physical fitness ↑

5	Karkou, V. et al. (2021)	N = 70	pre-treatment phase, during treatment phase, post-treatment phase	Mean Age (years): 53.51 ± 7.99	Cardiorespiratory fitness, Muscular Strength, Flexibility, Physical function	16 weeks	Exercise intervention (Supervised → etc.)	16week "Dancing with Health" program -→ Physical fitness ↑ (statistically significant)
6	Leach, H. J., et al. (2016)	N = 150	during treatment phase, post-treatment phase	Mean Age (years): 50.5 ± 8.7 Mean BMI (kg/m²): 26.3 ± 5.3	Cardiorespiratory fitness, Muscular Strength, Flexibility	12 weeks	Exercise intervention (Combined → Combined Exercise)	Over 24 weeks → flexibility ↑, aerobic fitness ↑ (VO_2 max, treadmill duration) strength ↔ (grip strength), core endurance ↔ (sit-up test)
7	Foley, M. P., et al. (2016)	N = 60	pre-treatment phase, during treatment phase, post-treatment phase	Mean Age (years): 59.7 ± 10.4 Mean BMI (kg/m²): 30.11 ± 0.93	Cardiorespiratory fitness, Muscular Strength, Flexibility, Physical function	12 weeks	Exercise intervention (Supervised → Combined Exercise)	12-week multimodal exercise program for breast cancer survivors (BCS) → Strength ↑, Flexibility ↑, Balance ↑
8	Leach, H. J., et al. (2015)	N = 96	during treatment phase, post-treatment phase	Mean Age (years): 50.3 ± 9.0 Mean BMI (kg/m²): 25.61 ± 4.66	Cardiorespiratory fitness, Muscular Strength, Flexibility	12 weeks	Exercise intervention (Combined → Combined Exercise)	12-week community-based exercise program (BEAUTY) for breast cancer survivors (BCS) during or within 3 months of treatment → VO_2 max, Grip strength, Flexibility: No change
9	Casla, S., et al. (2014)	N = 50	during treatment phase, post-treatment phase	Mean Age (years): 49.02 ± 7.83	Cardiorespiratory fitness, Muscular Strength	12 weeks	Exercise intervention (Supervised → Combined Exercise)	Exercise + education program → Physical capacity ↑, muscle strength ↑ in breast cancer patients

↑ indicates increased or improved; ↓ indicates decreased or deteriorated; ↔ indicates no meaningful or statistically significant change.

Table S9. Detailed breakdown of the 231 full-text articles excluded due to ineligible study design.

Exclusion category	No. of studies excluded
Review articles or meta-analysis	5
Study protocol or trial registrations	53
Conference or poster	144
Qualitative studies	16
Case-reports or case series	5
Editorials, letters, and commentaries	8
Total	231

Table S10. Study-level prevalence and record-level frequency of physical fitness domains.

Physical fitness domain	Unique studies assessing the domain (n)	Study-level prevalence, % (=316)	Record-level frequency, % (n=557)
Muscular strength	222	70.3	39.9
Cardiorespiratory fitness	190	60.1	34.1
Flexibility	100	31.6	18.0
Physical function	45	14.2	8.0

Study-level prevalence was derived from a study-by-domain binary matrix, in which each unique study was coded as 1 if a given physical fitness domain was assessed at least once and 0 otherwise. A single study could contribute to more than one domain. In this dataset, the number of unique studies assessing each domain was numerically identical to the corresponding PF domain record count because the extraction sheet contained one row per study-domain pair and no duplicate entries within the same domain for a given study. However, the percentages differed because study level prevalence used 316 unique studies as the denominator, whereas record-level frequency used 557 PF domain records as the denominator.

Table S11. Comparison of study-level phase distribution and record-level phase/context distribution.

Treatment phase/category	Unique studies assigned to the category (n)	Study-level distribution, % (=316)	Phase/context records, (n)	Record-level distribution, % (n=369)
Pre-treatment phase	5	1.6	8	2.2
During treatment phase	101	32.0	150	40.7
Post treatment phase	199	63.0	208	56.4
Unknown	2	0.6	3	0.8
Mixed phase	9	2.8	-	-
**Total**	**316**	**100.0**	**369**	**100.0**

Study-level distribution was calculated using 316 unique studies as the denominator, with each study assigned to one mutually exclusive category (pre-treatment, during treatment, post-treatment, unknown, or mixed phases). Record-level distribution was calculated using 369 treatment phase/context records as the denominator because a single study could contribute more than one phase/context record when participants were represented in multiple treatment phases or treatment contexts. Mixed-phase studies (n=9) are shown only in the study-level distribution; in the record-level analysis, these studies contributed to each applicable phase/context category rather than forming a separate record-level category.
